# Ultrafast underwater self-healing piezo-ionic elastomer via dynamic hydrophobic-hydrolytic domains

**DOI:** 10.1038/s41467-024-46334-4

**Published:** 2024-03-08

**Authors:** Zhengyang Kong, Elvis K. Boahen, Dong Jun Kim, Fenglong Li, Joo Sung Kim, Hyukmin Kweon, So Young Kim, Hanbin Choi, Jin Zhu, Wu Bin Ying, Do Hwan Kim

**Affiliations:** 1 Department of Chemical Engineering, Hanyang University, Seoul, 04763 Republic of Korea; 2 Ningbo Institute of Materials Technology and Engineering, Chinese Academy of Sciences, Ningbo, 315201 China; 3 School of Electrical Engineering (EE), Korea Advanced Institute of Science and Technology (KAIST), Daejeon, 34141 Republic of Korea; 4 Institute of Nano Science and Technology, Hanyang University, Seoul, 04763 Republic of Korea; 5 Clean-Energy Research Institute, Hanyang University, Seoul, 04763 Republic of Korea; 6 Present Address: Thin-Film Device Laboratory, RIKEN, 2-1 Hirosawa, Wako, Saitama, 351-0198 Japan

**Keywords:** Organic molecules in materials science, Polymer synthesis, Polymers

## Abstract

The development of advanced materials capable of autonomous self-healing and mechanical stimulus sensing in aquatic environments holds great promise for applications in underwater soft electronics, underwater robotics, and water-resistant human-machine interfaces. However, achieving superior autonomous self-healing properties and effective sensing simultaneously in an aquatic environment is rarely feasible. Here, we present an ultrafast underwater molecularly engineered self-healing piezo-ionic elastomer inspired by the cephalopod’s suckers, which possess self-healing properties and mechanosensitive ion channels. Through strategic engineering of hydrophobic C–F groups, hydrolytic boronate ester bonds, and ions, the material achieves outstanding self-healing efficiencies, with speeds of 94.5% (9.1 µm/min) in air and 89.6% (13.3 µm/min) underwater, coupled with remarkable pressure sensitivity (18.1 kPa^–1^) for sensing performance. Furthermore, integration of this mechanosensitive device into an underwater submarine for signal transmission and light emitting diode modulation demonstrates its potential for underwater robotics and smarter human-machine interactions.

## Abstract (web summary)

Elastomers capable of autonomous self-healing and mechanical stimulus sensing in aquatic environments are interesting for applications in underwater soft electronics. Here, the authors present a hydrophobic-hydrolytic molecularly engineered self-healing piezo-ionic elastomer with underwater self-healing properties and mechanosensitive ion channels.

## Introduction

Bionic skins, developed from iontronic materials, exhibit immense potential for diverse aquatic applications, including wearable electronics for divers^1^, underwater soft robotics^2^, and underwater tactile sensing^3,4^. These materials offer notable advantages, encompassing low energy consumption, exceptional sensitivity, and high spatial resolution^5–7^. Furthermore, their remarkable deformability facilitates efficient ion pumping and seamless integration. Nevertheless, a significant challenge arises owing to their susceptibility to unpredictable mechanical damage, resulting in performance degradation and device failure. Consequently, the incorporation of self-healing properties becomes crucial to restore functionality, extend device lifespan, and ensure long-term stability. Traditional self-healing iontronic materials, based on noncovalent bonds such as hydrogen bonds, ionic interactions, and metal-ligand coordination, are susceptible to water molecule absorption, which disrupts the dynamic bonds, compromising the self-healing properties^3,8,9^. Moreover, water molecule ingress can introduce electrical interferences, short circuits, and electrochemical corrosion, thereby disrupting the iontronic material’s conduction mechanism^4^.

To mitigate water ingress, previous studies have employed robust hydrophobic groups as a protective measure to shield the dynamic bonds and ionic interactions from water molecules. For instance, Cao et al.^3^ and Xu et al.^10^. employed C–F groups, which not only facilitated dynamic ion-dipole interactions as the driving force for self-healing but also imparted hydrophobicity, owing to the high electronegativity of fluorine and the robust electrostatic characteristics of C–F bonds. This design concept enabled the material to exhibit self-healing and sensing capabilities even in underwater conditions. However, these materials exhibited relatively low self-healing efficiencies and speeds owing to the limited synergistic effect between the hydrophobic groups and dynamic bonds^10–14^. Therefore, the development of iontronic materials capable of autonomous self-healing and superb sensing performance in aquatic environments, with high self-healing efficiency and speed, remains a substantial challenge.

Marine invertebrates, particularly cephalopods^15^, possess remarkable abilities to self-heal and perceive external stimuli, facilitating autonomous injury repair and interaction with their natural environment. An exemplar in this context is the squid, distinguished by its resilient biological materials comprising semicrystalline proteins forming ring teeth (RT) structures within its suckers. These RT proteins exhibit distinctive characteristics characterized by hydrogen bonds and abundant hydrophobic interactions, which significantly contribute to their self-healing and stabilization properties^16–18^. Additionally, another cephalopod species, the octopus, possesses specialized mechanosensory cells in its sucker epithelium^19^. These cells incorporate tethered ion channels, enabling the octopus to sense and respond to external mechanical stimuli within its surroundings.

Herein, we present the development of molecularly engineered self-healing piezo-ionic elastomer (MESHPIE) characterized by dynamic hydrophobic-hydrolytic domains, exhibiting exceptional self-healing properties in both aquatic and ambient environments, coupled with mechanosensitive piezo-ionic dynamics. Our inspiration stems from the observed self-healing capabilities within cephalopod’s RT structures and the presence of mechanosensitive ion channels in their sucker epithelium. To achieve this, we incorporated hydrophobic C–F side groups and hydrolytic boronate ester groups into a polyurethane (PU) matrix. When immersed in water, this composite could repel water molecules owing to the presence of dense hydrophobic barrier. Particularly noteworthy is the reversible hydrolysis exhibited by the dynamic boronate ester bonds^20,21^ when exposed to small quantities of water molecules, further accelerating the self-healing process. Optimization of the hydrophobic domain allows the ingress of small quantities of water while effectively repelling the majority of interfacial water molecules. This strategy prevents the complete hydrolysis of boronate ester bonds, ensuring efficient underwater self-healing capabilities. Through meticulous molecular engineering and systematic composition, we demonstrate that the coupling of hydrophobic and hydrolytic groups within a polymer can synergistically achieve high self-healing efficiency and speed at room temperature. Additionally, the introduction of 1-butyl-3-methylimidazolium bis(trifluoromethylsulfonyl)imide ([BMIM]^+^[TFSI]^–^) ionic liquid (IL) initiates a piezo-ionic mechanism, showing mechanosensitive ion trap and release characteristics. This behavior stems from the ion-dipole interactions between the C–F groups and ion pairs. To showcase the potential applications that harness the capabilities of our device, we integrated the mechanosensitive MESHPIE-based device into a toy submarine connected to a light-emitting diode (LED). This allowed us to visually represent pressure changes when the submarine collided with an underwater object, both before and after unexpected damage occurred. In addition, we demonstrated the device’s proficiency as a pressure-induced tactile sensor, efficiently modulating LED brightness.

## Results

### Mimicking the cephalopod

**Fig. 1:**
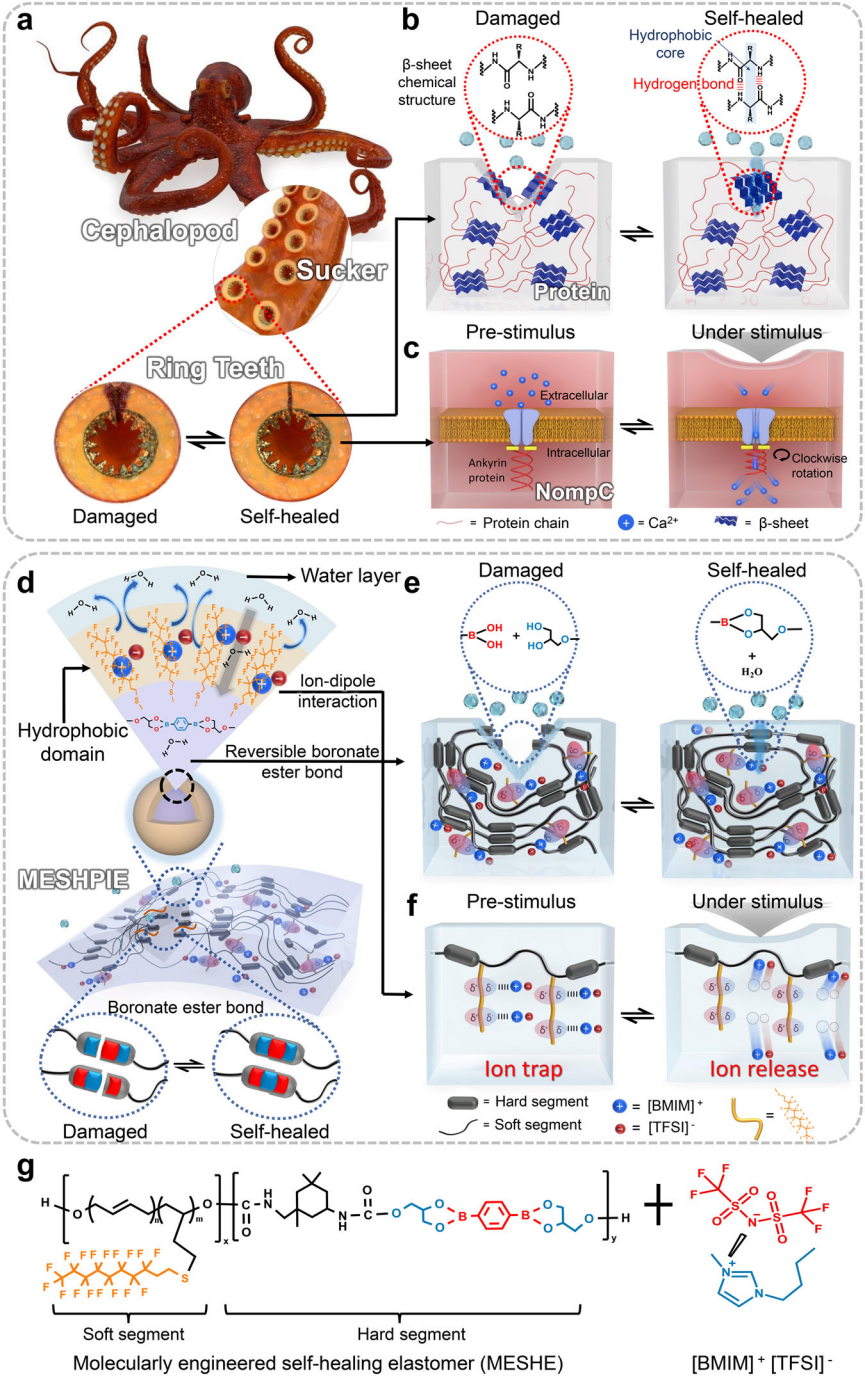
Molecular chemistry of the cephalopod and conceptual design principle of MESHPIE. **a** Schematics of cephalopod and ring teeth (RT) structures. **b** The RT proteins within the cephalopod’s suckers consist of numerous hydrogen bonds and hydrophobic cores, which enable self-healing in both ambient and aquatic conditions. The hydrogen bonds self-assemble into segmented semicrystalline morphology (amorphous and β-sheets). **c** Unique mechanoreceptor NompC composed of tethered ion channels observed in cephalopod’s suckers. **d** Chemical structure representation of MESHPIE consisting of hydrophobic domain and reversible boronate ester bonds for self-healing in both ambient and aquatic environments. **e** Schematic illustration depicting the structure of MESHPIE, consisting of hard and soft segments, emulating the RT protein structure of the cephalopod. **f** Piezo-ionic dynamics (trap and release mechanism) emulating the ion dynamics of the cephalopod. **g** Representation of the design chemistry of MESHE and ionic liquid.

The RT proteins found within the suckers of cephalopods (Fig. 1a) possess segmented semicrystalline architecture consisting of amorphous and crystal-forming domains (β-sheets)^16,17^ (Fig. 1b). These structures exhibit unique self-healing properties both in ambient and aquatic conditions, owing to their semicrystalline morphology, numerous hydrogen bonds, and hydrophobic groups^17,18,22^. Additionally, the suckers of the cephalopod contain mechanoreceptor NompC (no mechanoreceptor potential C), which is functionally equivalent to the NompC found in Drosophila^19,23^. These mechanoreceptor cells are composed of tethered ion channels that transduce external mechanical stimuli. The NompC ion channels consist of transient receptor potential (TRP) domains with ankyrin repeats. In their resting state, the TRP domains act as a gate, closing the transduction channel and preventing the entry of Ca^2+^ ions. However, under external stimulus, compression of the ankyrin repeats leads to a clockwise rotation of the TRP domains, resulting in the opening of the ion channel, allowing Ca^2+^ ions to enter (Fig. 1c)^19,23–27^.

Inspired by the RT proteins and the ion dynamics of mechanoreceptors in cephalopod suckers, the conceptual design of MESHPIE (Fig. 1d) aims to replicate the exceptional self-healing properties and mechanotransduction mechanism observed in cephalopods. The MESHPIE is developed from a C–F-functionalized PU matrix and reversible boronate ester bonds, which enable self-healing capabilities in both ambient and underwater conditions without the need for external stimuli. The C–F groups, depicted in Fig. 1d, exhibit dipole-dipole interactions that enhance self-healing properties in an ambient condition^28,29^. In addition, the low hydrogen acceptor and donor characteristics, as well as low surface energy of C–F groups, prevent interference with water molecules, allowing effective self-healing and underwater operation^3,10,30^. Compared to previous studies, the introduction of reversible boronate ester bonds (Fig. 1d, e), and the regulation of its hydrolysis reaction^20,21^, is the key principle to realize high underwater self-healing efficiency and speed. Additionally, the introduction of IL triggers a trap and release phenomenon through ion-dipole interactions between the [BMIM]^+^[TFSI]^–^ ion pairs and the C–F groups (Fig. 1f). The trapping of the ion pairs is facilitated by the high electronegativity and large dipole moment of the C–F groups^30^. Therefore, increasing the C–F groups is crucial to enhance self-healing ability in air, enabling effective underwater operation, and improving the ion trapping phenomenon. However, it is important to note that excessive C–F groups may decrease the underwater self-healing speed and efficiency owing to increased hydrophobicity, which limits the amount of water ingress required for effective self-healing via boronate ester bond hydrolysis. Figure 1g shows the chemical structure of MESHPIE consisting of the combination of molecularly engineered self-healing elastomer (MESHE) and IL.

### Molecular engineering principle and synthesis

**Fig. 2:**
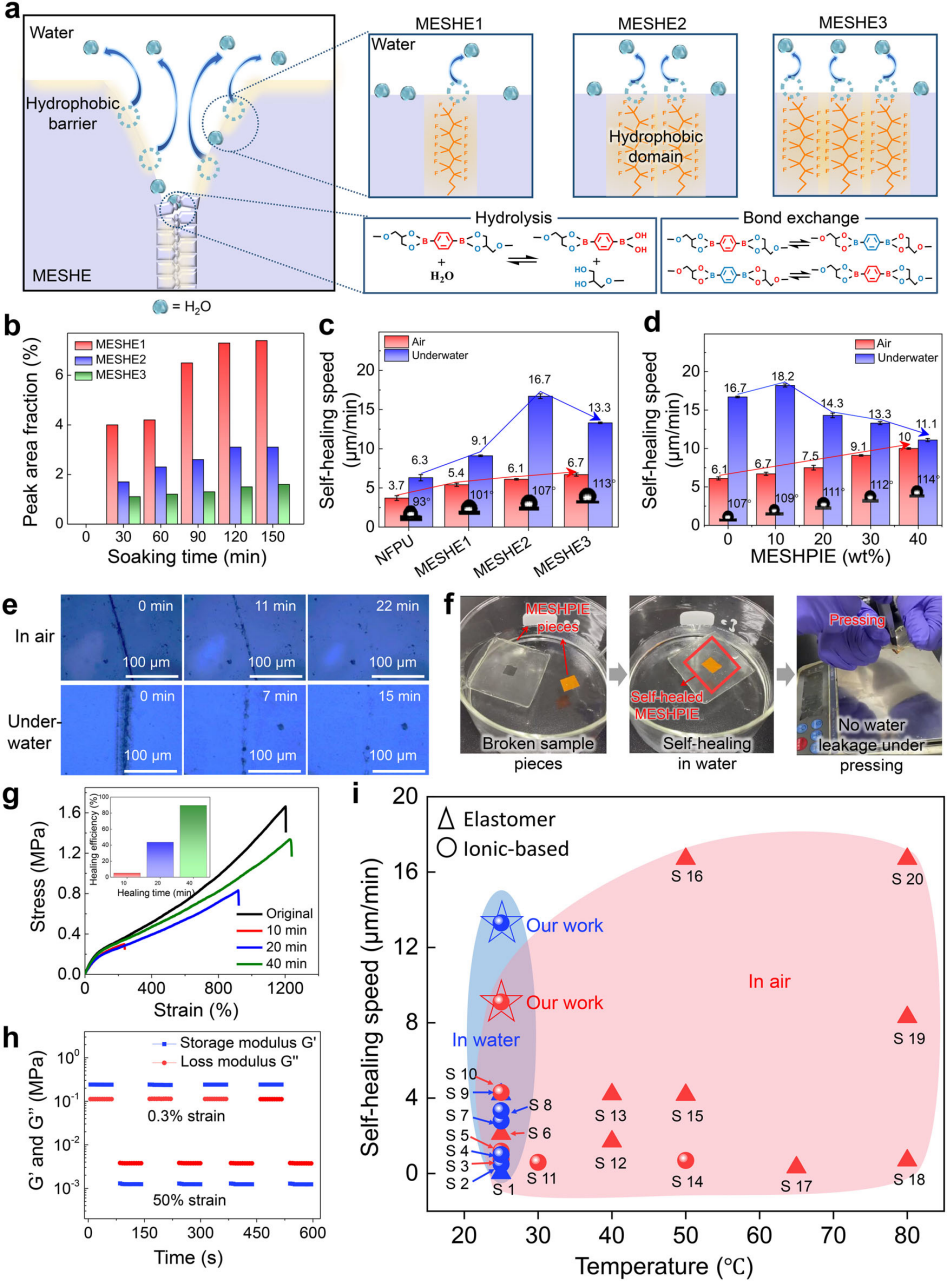
Hydrophobic properties and ultrafast, autonomous self-healing. **a** Schematic illustration depicting the regulation of water ingress via hydrophobic C–F domain in the various MESHEs. Additionally, the right-side bottom shows the chemistry of the reversible boronate ester bonds self-healing mechanisms in aqueous and ambient environments. **b** Analysis of the hydrolytic behavior of the various MESHEs at different immersion times. This graph represents the calculated peak area fraction of boronic acid group as a function of the soaking times. **c** Self-healing speeds of the various MESHEs in both ambient and underwater conditions. **d** Self-healing speeds of the various MESHPIEs with different IL concentrations in both ambient and underwater conditions. Error bars in c and d correspond to the standard deviations, obtained from more than five times healing tests of each sample. **e** Autonomous self-healing of a scar in air (RH 20–40%) and underwater at room temperature, observed under an optical microscope (scale bar 100 µm)**. f** Cut and spliced scenario of two different MESHPIE pieces, healed together underwater to withstand breaking under pressure. **g** Stress-strain curves of underwater self-healed films at various healing times. **h** Oscillatory time sweeps of MESHPIE with water at 25 °C. **i** Comparing the self-healing speeds of MESHPIE in both air (red) and underwater (blue) environments to other room temperature self-healing dielectric elastomers (squares) and ionic-based materials (circles). References are listed in the Supplementary information.

To construct the optimum hydrophobicity to ensure effective hydrophobic-hydrolytic synergy for rapid self-healing in both atmospheric and underwater environments, as well as effective ion trapping effect, a series of MESHEs with varied C–F groups were designed and synthesized. These included MESHE1, MESHE2, and MESHE3, with varying atomic concentrations of fluorine (16. 9%, 21.8%, and 28.8%, respectively). Detailed information on the design and synthesis methods can be found in the method section, Supplementary Note 1, and Supplementary Figs. 1–5. Although the various MESHEs share identical polymer structures, differences in morphology occur in their soft segments owing to varying hydrophobic chain densities based on the concentrations of C–F groups (see Supplementary Tables 1, 2). Figure 2a depicts the schematic illustrations of the various MESHEs designed to explore the influence of hydrophobic C–F groups density on regulating hydrolysis of boronate ester bonds. The variation tendency represented here stems from the transformation of the various hydrophobic C–F groups when in contact with water. Additionally, PU without any C–F groups termed NFPU (i.e., no C–F polyurethane, Supplementary Fig. 3b) was synthesized and used as a control. The water contact angle (WCA) revealed that MESHE3, with the highly packed hydrophobic C–F groups density exhibited the highest contact angle (113°), whereas NFPU exhibited the lowest contact angle (95°) as presented in Supplementary Fig. 6. The WCAs of the MESHEs remained stable even after immersion in deionized water for 5 days, indicating excellent hydrophobicity. Furthermore, the optimal boronate ester bond hydrolysis necessary for effective underwater self-healing was also verified by the content of the different C–F groups. Attenuated total reflection-Fourier transform infrared (ATR-FTIR) characterizations were performed to quantitatively analyze the degree of hydrolysis that occurs within the MESHEs. As shown in Supplementary Fig. 7, the characteristic peak at 1360 cm^–1^ represents boron oxygen groups (B–O) of the boronate ester bonds before immersion in water^31^. However, after immersion in deionized water for 30 min or more, a new characteristic peak attributed to boronic acid groups (-B(OH)_2_) appeared at 1341 cm^–1^, indicating hydrolysis of the boronate ester bonds^31^. The characteristic peaks at various immersion times were deconvoluted using PeakFit software, and the representative calculations are shown in Fig. 2b (see Supplementary Note 1, Supplementary Fig. 8, and Supplementary Tables 3, 5 for details). Moreover, Fig. 2b reflects the increasing concentration of boronic acid in the MESHEs with the increase in immersion time, revealing the highest water ingress inside MESHE1 (with the lowest hydrophobic chain density) and the lowest water ingress inside MESHE3 (with the highest hydrophobic chain density). This trend is consistent with the hydrophobicity analysis of the MESHEs, confirming that the higher the content of C–F groups, the lower the surface energy, resulting in higher hydrophobic chain density^10,32^.

### Role of hydrolytic boronate ester bonds and hydrophobic groups on ultrafast underwater self-healing

As explained earlier, boronate ester bonds are the main representative reversible covalent bond groups for self-healing in the MESHE system. The self-healing process of boronate ester bonds involves different mechanisms in air and underwater, as presented in Fig. 2a, right side bottom. In air, adjacent boronate esters undergo metathesis bond exchange reactions to repair themselves without the addition of water or excess free diol^33^. In an aqueous environment, boronate esters undergo hydrolysis/re-esterification reactions, wherein the ingress of water to the cut surfaces shifts the equilibrium of surface-exposed boronate esters toward hydrolysis, resulting in dissociated boronic acids and diols. Subsequently, these dissociated monomers participate in re-esterification reactions to form boronate ester bonds again^8,20,33,34^. Nevertheless, in the presence of excessive water ingress, the boronate ester groups undergo complete hydrolysis or dissociation reaction, yielding irreversible boronic acids and diols, a condition that hinders the potential for re-esterification during the self-healing process^9^. Thus, a combined effect of relative hydrophobicity to regulate the water ingress is necessary to establish reversible boronate ester bond formation for superb underwater self-healing properties^8^. Owing to the incompatibility between hydrophobic functional groups and water, water molecules induce the formation of orderly C–F groups, creating dense hydrophobicity facilitated by hydrophobic interactions^35,36^. The denser the hydrophobic interactions, the less water ingresses into the polymer system. Thus, relative hydrophobicity is beneficial to achieve efficient hydrophobic-hydrolytic synergy for underwater self-healing. As shown in Fig. 2c, the underwater self-healing speed of the MESHEs increased with higher C–F groups content. However, MESHE3, with the highest C–F groups content exhibited a drastic decrease in underwater self-healing speed, owing to denser hydrophobicity which limited the amount of water ingress necessary for effective self-healing through boronate ester bond hydrolysis. In addition, the self-healing speed in air also increased with the introduction of higher C–F groups owing to enhanced dipole-dipole interactions^28,29^

To construct ionically conductive MESHE (MESHPIE) for sensing application, various IL concentrations (10-40 wt%) were added to MESHE2 (with the excellent hydrophobic-hydrolytic synergy), to explore the optimum concentration for maximum device output performance and higher sensitivity. Figure 2d presents the water contact angles of MESHPIEs with different IL concentrations and their self-healing speeds in air and underwater. In air, we observed a stepwise increase in self-healing speed as the IL content increased, owing to the plasticization effect of ions on the polymer matrix, which enhanced polymer chain mobility^7,37^. In contrast, underwater self-healing speed increased slightly only in MESHPIE@10 wt% but drastically decreased with higher IL concentration. This phenomenon can be attributed to the increase in hydrophobic chain density imparted by the IL. Notably, [BMIM]^+^[TFSI]^–^ exhibits hydrophobic properties owing to the delocalization of negative charge and steric hindrance of [TFSI]^–^. Consequently, with an increase in the concentration of the hydrophobic IL, the hydrophobic nature of MESHPIEs was further intensified. This constrained the necessary water ingress for efficient hydrolysis, leading to decrease in self-healing speeds. To achieve a combined effect of excellent hydrophobic-hydrolytic synergy for ultrafast underwater self-healing properties and higher device sensitivity, the optimum condition was realized with MESHPIE@30 wt%. The details of the optimization process, including the determination of the optimum IL concentration and the preparation methods for MESHPIE, are discussed in the Method section, Supplementary Note 2, Supplementary Figs. 9–12, and Supplementary Table 6.

The capability of MESHPIE to heal damage and restore mechanical strength was studied in both ambient and aquatic environments. As shown in Fig. 2e, scratch tests were conducted to evaluate the autonomous self-healing capabilities of MESHPIE. The scars on the MESHPIE disappeared completely within 22 min in air (relative humidity, RH 20–40%), and within 15 min underwater at room temperature. Additionally, we performed a complete cut and healed scenario with two individual MESHPIE pieces to demonstrate the effective underwater self-healing properties (Fig. 2f and Supplementary Movie 1). To investigate the self-healing efficiency of MESHPIE, we analyzed the mechanical properties after self-healing in both air and underwater at different time intervals (Supplementary Fig. 14, Supplementary Tables 7–8, and Fig. 2g). MESHPIE exhibited self-healing efficiencies of 94.5% after 50 min in air and 89.6% after 40 min underwater. Additionally, the self-healing performance was characterized by rheological oscillation strain tests in air and with water (Supplementary Note 2, Supplementary Figs. 15a-c, and Fig. 2h**)**. MESHPIE in air and with water displayed excellent recovery of storage modulus (Gʹ) and loss modulus (Gʺ) after alternatively reducing the strain from 50% to 0.3%, repeatedly. The repeated damage-recovery tests demonstrated the robust recovery of MESHPIE to its original state without compromising its mechanical properties. Most importantly, MESHPIE exhibited superior self-healing speeds of 9.1 µm/min and 13.3 µm/min in air and underwater, respectively, as compared to other reported room temperature self-healing dielectric elastomers and ionic-based materials with various autonomous healing mechanisms (Fig. 2i and Supplementary Table 9). The C–F and boronate ester groups established synergistic effect between hydrophobic interactions and hydrolysis/re-esterification, by which the MESHPIE achieved high self-healing speeds and efficiencies.

### Molecular characterization

**Fig. 3:**
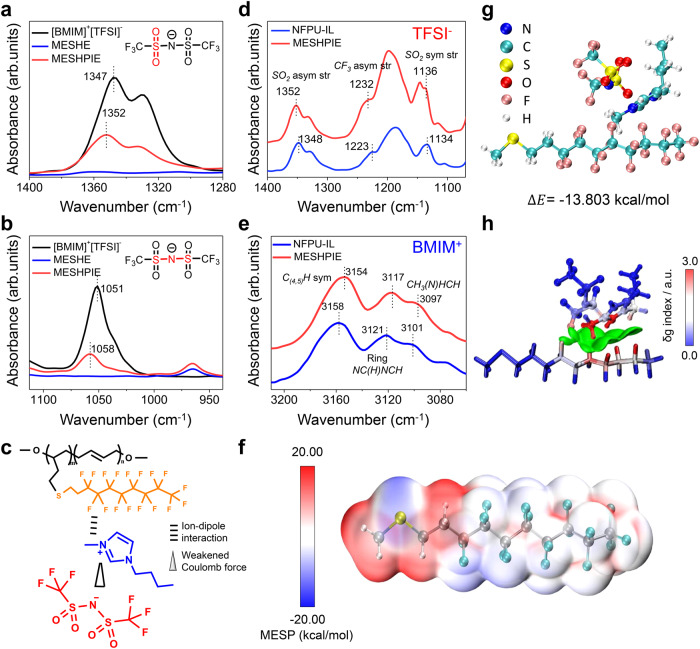
Molecular characterization of MESHPIE. ATR-FTIR characterization of [BMIM]^+^[TFSI]^-^, MESHE, and MESHPIE to verify the ion-dipole interaction. **a** The spectral range of 1400–1280 cm^−1^ and **b** 1120–920 cm^−1^ corresponds to the stretching of TFSI^–^ vibrational bands in MESHPIE owing to the weakened Coulomb forces generated by the ion-dipole interaction effect on the cations. **c** Schematic representation of ion–dipole interaction between C–F groups and the [BMIM]^+^ cation, and the Coulomb force between the ion pairs. ATR-FTIR spectra in the spectral regions of **d** 1400–1000 cm^−1^ (pertaining to TFSI^−^ stretching) and **e** 3210-3060 cm^−1^ (pertaining to BMIM^+^ stretching) of MESHPIE and NFPU-IL (used as reference). **f** Molecular electrostatic potential (MESP) of MESHPIE molecule and ion pair to predict the possible interaction site. **g** Complexation energy via quantum chemical computing of imidazolium cation [BMIM]^+^ in the presence of counter anion [TFSI]^–^ with C–F group to investigate the relative stability of their interactions. **h** The atoms of the complexes according to their contributions to the intermolecular interactions. Dark blue indicates no contribution to the complexation, and dark red indicates the largest relative contribution.

The C–F bonds are highly polarized groups owing to their high electronegativity and large dipole moment^3,10,30^. Hence, the IL interacts with the MESHPIE polymer chains via ion-dipole interactions, improving the compatibility of the IL with the PU matrix. The generated dipoles of C–F groups attract the [BMIM]^+^ cation in the IL leading to the trapping effect on [BMIM]^+^[TFSI]^–^ ion pairs, which initiates the trap and release phenomenon. To confirm the presence of ion-dipole interactions, the internal molecular structures of IL, MESHE, and MESHPIE were characterized via ATR-FTIR. As depicted in Fig. 3a and b, the characteristic spectra bands at 1347 cm^–1^ and 1051 cm^–1^ attributed to *SO*_*2*_ asymmetric and *S–N–S* asymmetric stretching in the [TFSI]^–^ shifted to 1352 cm^–1^ and 1058 cm^–1^, respectively^12,38^. These shifts towards higher wavenumber indicate the weakening of the Coulomb interactions between the IL pairs owing to the dragging away of the cations by the C–F dipoles^7,12^ as schematically illustrated in Fig. 3c. Moreover, we further analyzed the trapping effect of ions to the C–F group via molecular characterization of MESHPIE and NFPU-IL (NFPU with a 30 wt% IL, used as reference). The shifting of characteristic FTIR bands attributed to *SO*_*2*_, and *CF*_*3*_ bonds^39^ of [TFSI]^–^ in MESHPIE (Fig. 3d) towards higher wavenumbers confirms the additional freedom of the [TFSI]^–^ ions owing to the dragging away of the cations by the C–F dipoles via ion-dipole interactions^7,38^. Likewise, the corresponding shifts of *C*_*(4,5)*_*H, NC(H)NCH*, and *CH*_*3*_*(N)HCN* band peaks^40^ of [BMIM]^+^ (Fig. 3e) toward lower wavenumbers strongly support the trapping effect of C–F groups on the cations. In addition, Visual Molecular Dynamics (VMD) program (Fig. 3f), quantum chemical computing (Fig. 3g), and independent gradient model (IGM) (Fig. 3h) analyses were also conducted to predict the possible interaction site, attractive binding energies, and conformal stability of the interactions, respectively (see Supplementary Note 3 and Supplementary Fig. 16 for details). The optimized geometries showed different attractive binding energies with the most conformal stability at −13.803 kcal/mol.

### Characterization of piezo-ionic dynamics

**Fig. 4:**
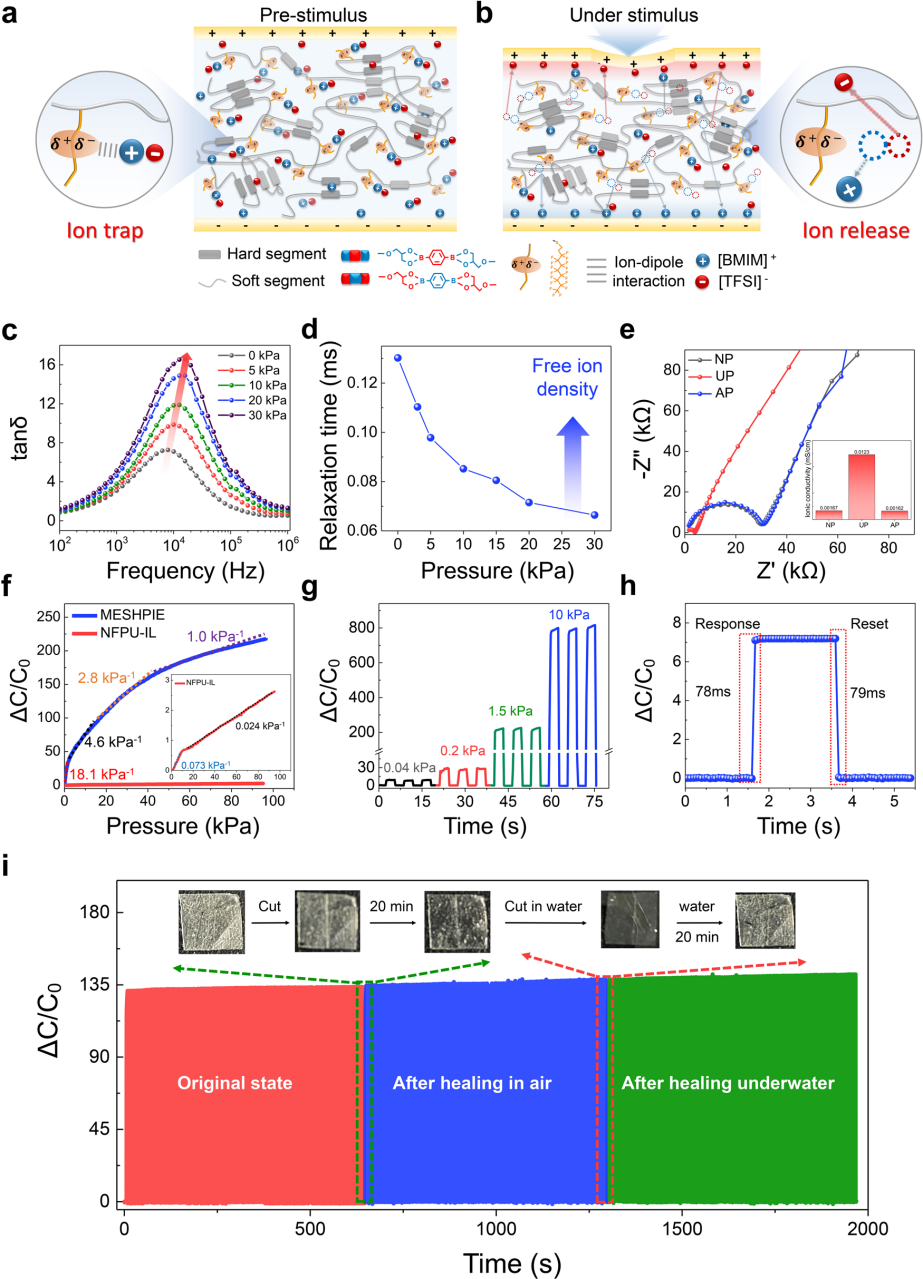
Conceptual design, sensing mechanism (piezo-ionic dynamics), and sensing performance of MESHPIE-based device. The design of MESHPIE-based device consists of MESHPIE sandwiched between two deformable AgNW/MESHE2 electrodes with voltages of 1 mV to 1 V. **a** At a pre-stimulus condition, ionic species are trapped to C–F groups (trapped state). **b** Under stimulus, MESHPIE exhibits ion pumping phenomenon owing to pressure-induced breaking of ion-dipole interactions. **c** Ion dynamics and free ion number concentration under increasing pressures. **d** Variation tendencies of charge relaxation time as a function of increasing applied pressures. **e** Impedance Nyquist plots of MESHPIE-based device for no pressure (NP), under pressure (UP), and after removing pressure (AP) conditions, strongly affirming the reversible ion movement. **f** Comparison of pressure sensitivities (100 mV applied bias at 20 Hz) of MESHPIE and NFPU-IL (insert, red) (used as a reference). **g** Pressure-dependent capacitance changes of MESHPIE as a function of applied pressures (0.04 kPa, 0.2 kPa, 1.5 kPa, and 10 kPa) with respect to time (100 mV applied bias at 100 Hz). **h** Transient response time at a loading pressure of 7.2 kPa (1 mV applied bias at 1000 Hz). **i** Mechanical durability test of the MESHPIE-based sensor device after self-healing in air and underwater (200 cycles each, 1 mV applied bias at 100 Hz).

The MESHPIE-based device consists of MESHPIE sandwiched between two deformable Ag nanowire (AgNW)/MESHE2 electrodes. The device architecture as illustrated in Fig. 4a describes the piezocapacitive pressure-sensing mechanism (known as mechanosensitive piezo-ionic dynamics), exhibiting electrical double layer (EDL) at the MESHPIE/electrode interface. At pre-stimulus, most of the [BMIM]^+^[TFSI]^–^ ion pairs are trapped to the C–F groups with limited freedom of motion. Nonetheless, few of the ion pairs exist independently through intercalation between the PU hard segments^37^ as explained in Supplementary Note 2 and Supplementary Fig. 10. Under external stimuli such as pressure, the MESHPIE-based device gradually deforms, reducing the distance between the top and bottom electrodes, which induces a strong electric field (Fig. 4b). The deformation and pressure-mediated breaking of ion-dipole interactions result in the dissociation of the ion pairs from the C–F groups, creating the mechanosensitive piezo-ionic dynamics. The phenomenon of ion trap and release before and after inducing pressure, generates low initial capacitance and high final capacitance, respectively, owing to the efficient pumping of ions. This ensures effective control of piezo-ion dynamics, providing high device sensitivity, as well as high signal-to-noise level.

To support the concept of ion trap and release phenomenon, we investigated the relationship between piezo-ionic dynamics and the changes in complex impedance behavior^41^. This was accomplished by conducting stress relaxation tests on both MESHPIE and NFPU-IL (without C–F groups, used as reference), under applied pressure (Supplementary Note 4). As external pressure is applied to MESHPIE-based device, the impedance gradually decreased with increasing pressure (Supplementary Fig. 17a), owing to the increase in the free ion concentration (ionic strength) under applied pressure^41–43^. Meanwhile, the impedance of NFPU-IL-based device exhibited no substantial change under applied pressure (Supplementary Fig. 17b), as no trapping of ions occurred at the initial state, thus pressure-mediated ion pumping effect was not expected under external pressure. Additionally, increasing pressure results in the shift of charge relaxation frequency (τ^–1^) towards higher frequencies, owing to faster ionic atmosphere relaxation caused by increased free mobile ion concentration^42,44^ in MESHPIE as depicted in Fig. 4c, which was not an observed tendency in NFPU-IL (Supplementary Fig. 17c). Thus, with increasing pressure in MESHPIE-based device, the charge relaxation time gradually decreased (Fig. 4d) confirming the release of ions under applied pressure (see Supplementary Note 4 for further details). Furthermore, electrochemical impedance spectroscopy (EIS) Nyquist plots (Fig. 4e) under no pressure (NP), under pressure (UP), and after removing pressure (AP) were also obtained to confirm the reversible ion movement in MESHPIE after removal of external stimuli. Meanwhile, there was no noticeable reversible ion movement in NFPU-IL (Supplementary Fig. 18), mainly because most of the ion pairs are already located within the free volume of the polymer matrix.

### Pressure-induced sensing performance

Next, the pressure-sensing performance of various MESHPIE-based devices were explored under various applied bias voltages as a function of different frequencies (Supplementary Note 5, Supplementary Figs. 19, and 20). As discussed earlier, an increase in pressure leads to the release of more free mobile ions that were initially trapped, thereby strengthening the EDL formation. In addition, the C–F groups also enhanced the overall capacitance of the MESHPIE-based device owing to improved dielectric constant as presented in Supplementary Fig. 21. Notably, the introduction of fluorinated groups in the soft segment chain enhanced the polymer polarity via induced dipole moment, creating higher dielectric constant which subsequently contributed to the higher capacitance value^45,46^. Thus, the MESHPIE-based device exhibited a higher initial capacitance value than the NFPU-IL-based device. Furthermore, the pressure sensitivity, defined as *S* = *δ(ΔC/C*_*0*_*)/δP*, (where *C*_*0*_ denotes the initial capacitance without applied pressure, *ΔC* denotes capacitance change, and *P* denotes applied pressure) of the MESHPIE (*S* = 18.1 kPa^−1^ – 1.0 kPa^−1^) is significantly higher compared to that for the NFPU-IL (*S* = 0.073 kPa^−1^– 0.024 kPa^−1^) over a wide pressure range (0 ~ 95 kPa) (Fig. 4f). The high-pressure sensitivity of MESHPIE device can be attributed to C–F groups-initiated piezo-ionic dynamics (generating high *C*_*p*_*/C*_*0*_ values), and enhanced dielectric constant. The recovery and time-dependent behaviors were observed under dynamically increasing pressures (Fig. 4g). This confirms the reproducibility of the capacitance signals, demonstrating a highly durable MESHPIE-based device. An excellent response and reset time were also obtained for the pressure sensitive device (Fig. 4h). To demonstrate the pressure-sensing response of self-healed MESHPIE, durability tests were performed after self-healing in both air and underwater. As depicted in Fig. 4i, loading–unloading cyclic tests confirmed not only the phenomenal durability, but also the excellent retention of sensing properties even after self-healing in air and underwater.

**Fig. 5:**
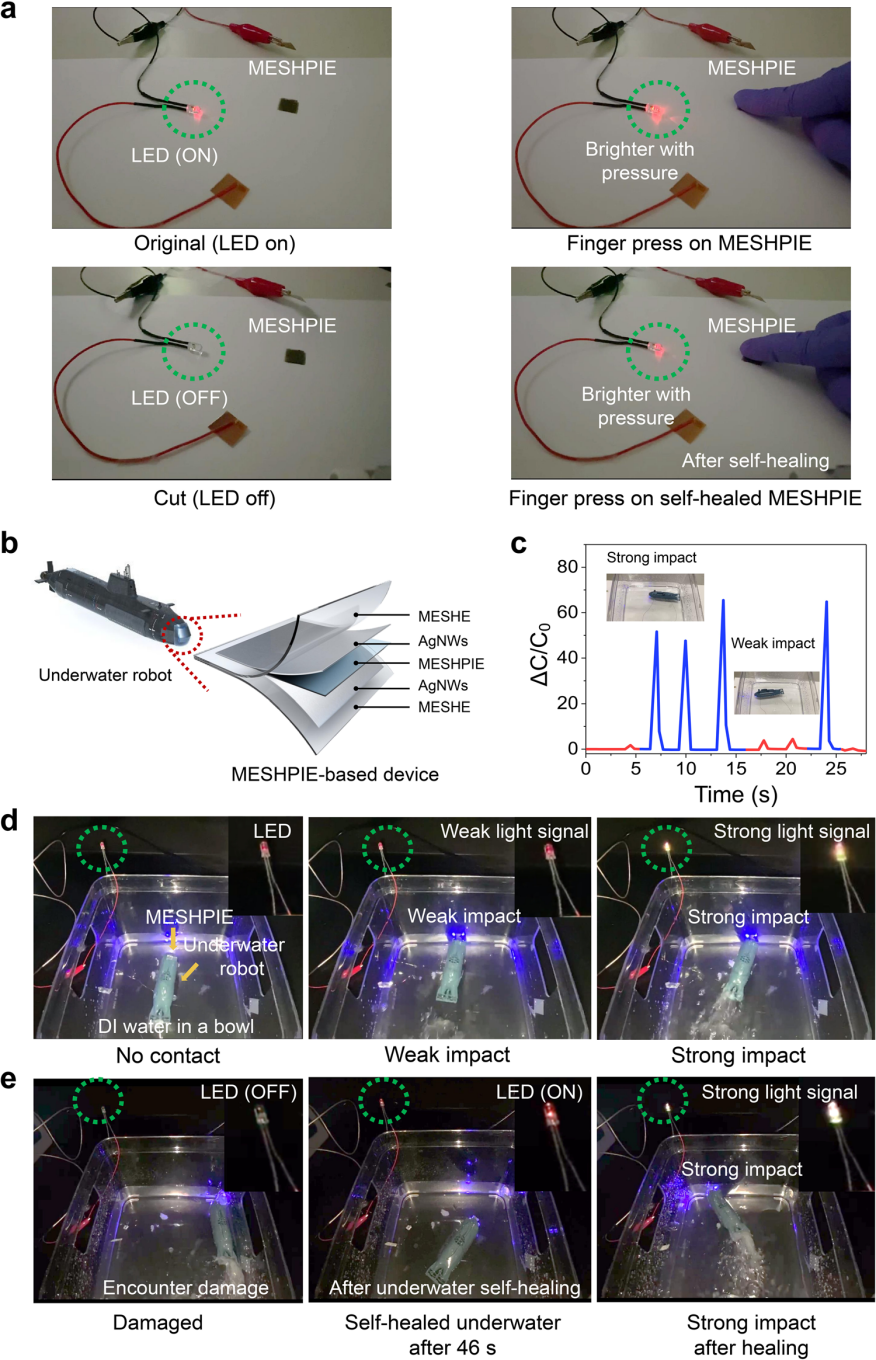
Demonstration of practical device performance in ambient and aquatic environments. **a** Changes in LED intensities under touch-induced pressures, before and after self-healing situations. **b** Illustration of MESHPIE-based device architecture, which is incorporated into an underwater toy submarine for practical demonstration. **c** Sensing responses upon impact with underwater object (wall of the container). **d** Photographs showing the visualization of changes in LED intensity upon impact with underwater object. **e** Pressure response of self-healed device after encountering severe damage underwater.

To demonstrate the potential of our device for practical applications in human-machine interfaces and underwater robotics, we performed various representative illustrations. First, the device was used as a pressure-induced tactile sensor to regulate LED intensity (Fig. 5a and Supplementary Movie 2). When a finger touched the MESHPIE-based device before and after self-healing, the applied pressure led to an increase in the intensity of the LED. These phenomena were attributed to the capacitive coupling of AC power supply^7,47^ and pressure-mediated ion-pumping effect in MESHPIE, decreasing the device’s bulk resistance and subsequent increase in LED intensity (Supplementary Note 6). Next, we demonstrated underwater sensing performance by incorporating the device into an underwater toy submarine (Fig. 5b). The device was initially connected to an onboard precision LCR meter (see methods section for details) to record the sensing signal upon direct impact with an underwater object (wall of the container). As presented in Fig. 5c and Supplementary Movie 3, weak and strong sensing signals were obtained when the underwater robot made a weak or strong impact with the wall of the container, respectively. The signal producing mechanism is equivalent to the application of low pressure (weak impact) and high pressure (strong impact) on the device, causing a piezo-ionic phenomenon for the respective changes in capacitance. Additionally, the device was connected in series to a roof-mounted LED to visualize changes in pressure upon collision with an underwater object. As depicted in Fig. 5d and Supplementary Movie 4, a weak and strong head-on collision with the wall of the container produced a slight and strong increase in LED intensity, respectively. Moreover, a sharp blade was submerged into the container to inflict damage to the device upon direct collision. Figure 5e and Supplementary Movie 5 show that the LED intensity dimmed completely, signifying severe damage inflicted upon the device. However, after 46 s, not only was the LED intensity restored but also the device exhibited superb impact response. This strongly confirms not only the underwater sensing performance, but also the autonomous underwater electrical self-healing capabilities (evidenced in Supplementary Movie 6). These demonstrations offer compelling evidence of the device’s potential for effective underwater sensing and its capability to detect human interactive activities through touch, owing to its remarkable mechanosensitivity.

## Discussion

We have molecularly engineered a mechanosensitive piezo-ionic elastomer with dynamic hydrophobic-hydrolytic domains, demonstrating remarkable self-healing properties and pressure sensing capabilities in both ambient and aquatic environments. The material exhibits autonomous, ultrafast self-healing speeds of 9.1 µm/min in air and 13.3 µm/min underwater. Notably, the synergy between hydrophobic chain density and boronate ester hydrolysis is pivotal in achieving high underwater self-healing efficiency (89.6%) and speed. The boronate ester bonds undergo metathesis bond exchange reactions in air and reversible hydrolysis reactions with minimal water exposure, facilitating enhanced self-healing speed in aquatic environments.

The hydrophobic C–F side groups establish dense hydrophobic barrier, repelling the majority of water molecules, thereby shielding the boronate ester bonds and ionic interactions from complete hydrolytic reactions, enabling effective self-healing and sensing in underwater condition. Furthermore, the C–F groups play a crucial role in facilitating ion-pumping phenomenon established via ion-dipole interactions between ions and C–F dipoles, enabling superb mechanosensitivity. We integrated our MESHPIE-based device into an underwater toy submarine for signal transmission and LED illumination to visually indicate collisions with an underwater object. Additionally, we successfully demonstrated the device’s capacity to function as a tactile sensor, modulating LED intensity in response to applied pressure. These demonstrations emphasize the potential applications of our self-healing piezo-ionic device in soft electronics, underwater robotics, smarter human-machine interfaces, and innovative wearable technologies.

## Methods

### Preparation of molecularly engineered self-healing elastomer (MESHE)

Materials: Anhydrous tetrahydrofuran (THF, 99.5%), isophorone Diisocyanate (IPDI, 99%), hydroxyl-terminated polybutadiene (HTPB, Mn ≈ 3000 g mol−1), Glycerol (99.5%), 1,4-Benzenediboronic acid (BA), magnesium sulfate, dibutyltin dilaurate (DBTDL, 98%) were purchased from Aladdin (China) and used without further purification. 1-Butyl-3-methylimidazolium bis(trifluoromethylsulfonyl)imide ([BMIM]^+^[TFSI]^–^, 98%), *N, N*-dimethylformamide (DMF, 99.8%), 1H,1H,2H,2H-perfluorodecanethiol (PFDT, 97%), 2,2’-Azobis(2-methylpropionitrile) (AIBN) solution (0.2 M in toluene) were obtained from TCI (South Korea). Tetramethysilane (TMS) was utilized as an internal standard. The preparation of MESHE involved three main processes.

(i) The synthesis of chain extender [glyceryl benzenediborate (GBDB)]. As illustrated in Supplementary Fig. 1a, the synthesis route of GBDB involves dissolving 1,4-Benzenediboronic acid (3.0 g, 18.1 mmol) and glycerol (3.41 g, 37.1 mmol) in dry tetrahydrofuran (50 ml) with the addition of anhydrous magnesium sulfate (5.0 g). After stirring at room temperature for 24 h, solid magnesium sulfate and a transparent solution of the target product were obtained after filtration. The product solution was rotary-evaporated to obtain white solid, which was purified by repeated washing and suction filtration with a large amount of n-butanol. The synthesized chain extender GBDB was confirmed by ^1^H-Nuclear magnetic resonance spectroscopy (^1^H NMR) (Supplementary Fig. 2a).

(ii) The functionalization of soft segment mHTPB. In a glove box filled with 99.999% Ar, HTPB was dissolved in a toluene solution containing AIBN, and PFDT was added after complete dissolution. The solution was placed in an oil bath at 65 °C equipped with magnetic stirring and allowed to react for 24 h. Finally, the sample was placed in a vacuum oven at 60 °C for 48 h. Through these reactions, PFDT was incorporated into the double bond of the HTPB via click chemistry to form mHTPB (the functionalized HTBP) (Supplementary Fig. 1b). The synthesized soft segment mHTPB was confirmed by ^1^H NMR (Supplementary Fig. 2b).

(iii) The synthesis of MESHE. In a glove box filled with 99.999% Ar, the reactants mHTPB, chain extender GPDB in the ratio of 10:2, catalyst DBTDL, and organic solvent anhydrous THF were added into a three-neck reactor equipped with a mechanical stirrer. After the reactants were completely dissolved in the solvent, isophorone diisocyanate (IPDI) was added using a vacuum syringe. The reaction was heated at 65 °C, for 6 h. The mass concentration of all reactants was 45 wt%, and the mass concentration of DBTDL was 1 wt%. The target product was washed several times with methanol, and then dried in a vacuum oven at 60 °C for 24 h to obtain MESHE (Supplementary Fig. 3a). By varying the atomic concentrations of F (16.9%, 21.8%, and 28.8%) in mHTPB (Supplementary Table 2), we obtained MESHE1, MESHE2, and MESHE3, respectively. Similarly, NFPU (Supplementary Fig. 3b) was synthesized under identical conditions as described above except that mHTPB was substituted with HTPB (without C–F group functionalization).

Then MESHPIE was prepared by dissolving MESHE2 and 30 wt%[BMIM]^+^[TFSI]^–^ (1-butyl-3-methylimidazolium bis(trifluoromethylsulfonyl)imide, Sigma-Aldrich) ionic liquid (IL) in deuterated chloroform and deuterated tetrahydrofuran (TCI Japan), while stirring at room temperature for 8 h. To achieve the required thickness, a specific amount of the solution was poured into a square Teflon mold and annealed for 48–72 h at 80 °C under optimized conditions (initially starting at 40 °C with temperature intervals of 10 °C/h). Likewise, NFPU-IL (used as reference) was prepared using the same procedure as described above. Notably, in this study, the weight percentage of IL represents the weight ratio of IL to IL + PU. Hence, NFPU-IL consisted of 30 wt% IL + NFPU.

### Fabrication of self-healing electrodes

To design the self-healing electrodes, Ag nanowire (AgNW) solution (4 g ml^–1^) was prepared by diluting an AgNW suspension (Nanopyxis Corp., diameter: 32 ± 5 nm length: 25 ± 5 µm) in IPA. The AgNW solution was spray coated (SRC-200 VT, E-FLEX Korea, nozzle; 0.05 mm, pressure; 200 mbar) onto a pre-designed demarcated Teflon mold and subjected to heat treatment of 90 °C for 30 min to evaporate excessive IPA solution, reducing inter-nanowire resistance. Subsequently, a solution of MESHE2 was drop-cast onto the AgNW-coated Teflon mold and then annealed under the optimized conditions discussed earlier. As a result, the AgNW percolation network was transferred onto the MESHE2 film. The film was then peeled off from the Teflon mold to form the AgNW/MESHE2 electrode, and its conductivity was measured using a digital multimeter before proceeding with further experiments. To establish connection with measuring instrument, Ag wires (Nilaco Corp., diameter: 50 µm) were attached to the electrodes.

### Material characterization and self-healing tests

The chemical compositions of GPDB, mHTPB and MESHEs were confirmed by ^1^H NMR recorded on a Bruker AVIII400 NMR spectrometer at 25°C. Weight-average molecular weights (Mw) and the polydispersity index (PDI) were measured using gel permeation chromatography (GPC, Waters-2690) with THF as the mobile phase at 40 °C. Universal testing machine (UTM, Instron 5567) equipment was utilized to investigate the mechanical properties of the MESHEs at room temperature. The strain rate was controlled at 50 mm min^–1^ using dumbbell–shaped splines with dimensions of 35 mm (length), 2 mm (width), and ~ 0.5 mm (thickness). The Young’s modulus was determined based on the slope of the stress–strain curves (0–5% strain values). Water contact angle (WCA) analyses were performed with a contact angle goniometer (OCA25, DataPhysics, Germany). The water droplet was deposited using a syringe pointed downward toward the sample film surface. X-ray diffraction analysis was performed using a Bruker D8 Advance diffractometer with Cu-Kα radiation (wavelength = 1.54060 Å). Diffraction scans were performed at an angle of 5–50^o^ 2θ, with a scan speed time of 0.32 s. The chemical surface analyses were carried out using an X-ray photoelectron spectroscopy (XPS, PHI 5000 Versa Probe II, ULVAC-PHI, Inc.). Attenuated total reflection-Fourier transform infrared (ATR-FTIR) spectroscopy spectra were obtained using a Bruker Optics GmbH (ZnSe instrument, Germany) spectrometer in the absorption mode. Each spectrum was obtained as the average of 64 scans (4 cm^–1^ resolution) recorded from 500 to 4000 cm^–1^. For the self-healing experiments, a scratch recovery tests were performed under a real-time optical microscope (Olympus/BX 51TF Instec H601, Japan) for various time periods, at both ambient (20–40% relative humidity) and underwater conditions (DI water). In addition, 0.2 mm film was cut into two individual pieces and re-joined underwater for self-healing.

### Electrical characterization

Electrochemical impedance spectroscopy (EIS) is a powerful technique to investigate the complex impedance behavior of mobile ions and ion transport characteristics in polymer electrolytes, as well as their electrode/electrolyte interfaces. In this study, EIS data were collected at room temperature using an electrochemical analyzer PGSTAT302N (Metrohm Autolab) in a 0.1 Hz–1 MHz frequency range with a 10 mV alternating current (AC) signal. To evaluate the ionic conductivity, a coin cell (Hohsen Corp., Japan) was utilized to perform EIS measurements on various films. The obtained impedance spectra were fitted using appropriate equivalent circuit models built in NOVA software (Metrohm Autolab), enabling the evaluation of the bulk resistance (*R*_*b*_) of the devices. The ionic conductivity was subsequently calculated based on the obtained bulk resistance values as *σ* = *(l/R*_*b*_ *x* *A)*, where *σ* denotes ionic conductivity, *l* denotes thickness of film, and *A* denotes the electrode area. Capacitance measurements were conducted by sandwiching the MESHPIE (thickness ~250 µm, area 1 cm^2^) between two AgNW/MESHE2 electrodes (1.2 cm×1.2 cm, thickness of 250 µm) in a Metal-Insulator-Metal (MIM) piezocapacitive device configuration (MESHPIE-based device). Ag wires (Nilaco Corp., diameter: 50 µm) were attached to the electrodes to establish a connection with a precise LCR meter (Agilent Keysight Technologies, E4980A).

### Computational details

The theoretical calculations were performed via the Gaussian 16 suite of programs. The structures of the studied systems (denoted by PFDT, [BMIM]^–^[TFSI]^+^) were fully optimized at the B3LYP-D3BJ/def2-SVP level of theory. The Visual Molecular Dynamics (VMD) program was used to plot the color-filled iso-surface graphs to visualize the molecular electrostatic potential (MESP) of PFDT to predict the possible interaction sites. Four different structures of the complexes between PFDT and BMIM-TFSI were built and optimized at the B3LYP-D3BJ/def2-SVP level. The vibrational frequencies of the optimized structures were carried out at the same level. The structures were characterized as a local energy minimum on the potential energy surface by verifying that all the vibrational frequencies were real. The interaction energies (ΔE) of the four optimized complexes were calculated to compare their relative stability. Δ*E* of the optimized complexes was calculated at the B3LYP-D3BJ/def2-SVP level of theory. Δ*E* is defined as the difference between the complex and the sum of energies of monomers, which can be obtained by the following formulas Δ*E* = E(AB)-E(A)-E(B). Independent gradient model (IGM) analysis was carried out for the most stable structure of the complex by using the Multiwfn software to deeply understand the nature of the intermolecular interaction. The VMD program was used to plot the color-filled iso-surface graphs of IGM analytical results.

### Pressure response and sensitivity analysis

To investigate the MESHPIE-based piezo-ionic sensors response to applied pressure, a custom-made sensor probe station equipped with a programmable *xy-* and *z-*axis stage (0.1 µm resolution) and a force gauge (Mark-10, 0.005 N resolution) was employed. The applied pressures were determined by dividing the force load by the dimensions of the unit film being pressed. Furthermore, the sensitivities of the piezocapacitive pressure sensor device to various pressure ranges were evaluated based on the slope of the relative capacitance changes versus applied pressure in real-time. The instrument was connected to a customized LabView-based program for the in situ simultaneous recording of capacitance changes and applied force load.

### Device demonstration system

For the demonstration, the MESHPIE-based device in a MIM (top electrode/MESHPIE/bottom electrode) structure configuration described in the previous section, was prepared as depicted in Fig. 5b. The device was then integrated into a toy submarine (12 cm × 3 cm x 4.2 cm), connected to a precise LCR meter or LED (voltage: 1.9 V ~ 2.2 V; current: 5 mA~20 mA; diameter: 5 mm) for the subsequent experiment. For the LED-based experiments, the device demonstration was set-up in a circuit configuration where a pulse generator (Keysight Technologies, 33500B series waveform generator) was employed to supply AC power to activate the circuit.

### Supplementary information

Supplementary Information

Peer review file

Description of additional supplementary files

Supplementary Movie 1

Supplementary Movie 2

Supplementary Movie 3

Supplementary Movie 4

Supplementary Movie 5

Supplementary Movie 6

## Data Availability

All relevant data that support the results of this study are available within the article and its supplementary information files. Further data is available from the corresponding authors upon request.
