# Supplementary material for: Ultrafast underwater self-healing piezo-ionic elastomer via dynamic hydrophobic-hydrolytic domains
Source: Nat Commun. 2024 Mar 8;15:2129. doi: 10.1038/s41467-024-46334-4 (PMC10923942; doi:10.1038/s41467-024-46334-4)
Supplement: Supplementary file 1 — Supplementary Information [file 41467_2024_46334_MOESM1_ESM.pdf]

Supplementary Information

# Ultrafast underwater self-healing piezo-ionic elastomer via dynamic hydrophobic-hydrolytic domains

Zhengyang Kong[1]†, Elvis K. Boahen[1]†, Dong Jun Kim[1], Fenglong Li[2], Joo Sung Kim[1‡], Hyukmin Kweon[1], So Young Kim[1], Hanbin Choi[1], Jin Zhu[2], Wu Bin Ying[1,3*], and Do Hwan Kim[1,4,5*]

[1]*Department of Chemical Engineering, Hanyang University, Seoul 04763, Republic of Korea.*

[2]*Ningbo Institute of Materials Technology and Engineering, Chinese Academy of Sciences, Ningbo 315201, People's Republic of China.*

[3]*School of Electrical Engineering (EE), Korea Advanced Institute of Science and Technology (KAIST), Daejeon 34141, Republic of Korea*

[4]*Institute of Nano Science and Technology, Hanyang University, Seoul 04763, Republic of Korea.*

[5]*Clean-Energy Research Institute, Hanyang University, Seoul 04763, Republic of Korea.*

†These authors contributed equally to this work.

‡Current address: Thin-Film Device Laboratory, RIKEN, 2-1 Hirosawa, Wako, Saitama 351-0198, Japan.

*Corresponding authors' email: yingwubin@kaist.ac.kr (W.B.Y.); dhkim76@hanyang.ac.kr (D.H.K.)

**This PDF file includes:**

Supplementary Notes 1 to 6

Supplementary Figures 1 to 23

Supplementary Tables 1 to 9

Supplementary References 1 to 17

# Supplementary Notes

**Supplementary Note 1. Synthesis and design principle of molecularly engineered self-healing elastomer (MESHE).**

The design and preparation of MESHE consists of three main steps: (i) the synthesis of chain extender, (ii) the functionalization of soft segment mHTPB, and (iii) the synthesis of MESHE. As illustrated in Supplementary Fig. 1a, glyceryl benzenediborate (GBDB) chain extender was synthesized from benzenediboronic acid and glycerol under optimized conditions as explained in the Method section (Main Manuscript). Next, hydroxyl-terminated polybutadiene (HTPB) was functionalized via click chemistry using 1H,1H,2H,2H-perfluorodecanethiol (PFDT) and 2,2'-Azobis(2-methylpropionitrile) (AIBN) as an initiator to create mHTPB (Supplementary Fig. 1b). Detail synthesis procedure is discussed in the Method section (Main Manuscript). The synthesized chain extender and mHTPB were confirmed by $^{1}$H-Nuclear magnetic resonance spectroscopy ($^{1}$H NMR) (Supplementary Fig. 2).

MESHE was synthesized through a one-step polymerization approach using GBDB, mHTPB, and isophorone diisocyanate (IPDI) in the ratio of GBDB:mHTPB:IPDI = 2.5g:10g:3.1g and dibutylin dilaurate as catalyst (Supplementary Fig. 3a). Likewise, no C–F polyurethane (NFPU) (Supplementary Fig. 3b) was also synthesized under identical conditions as mentioned above except that mHTPB was substituted with HTPB (without C–F group) and used as control. The GBDB and IPDI form the hard segment, and the mHTPB forms the soft segment of the MESHE. Various MESHEs were synthesized by optimizing the molar ratios of double bonds in HTPB to PFDT in the soft segment in the ratio of MESHE1 (HTPB 1:0.05 PFDT), MESHE2 (HTPB 1:0.10 PFDT), and MESHE3 (HTPB 1:0.15 PFDT) (Supplementary Tables 1 and 2), to explore the optimum concentration of C–F groups for optimum hydrophobicity, fast self-healing speed in both air and underwater, as well as maximum ion trapping effect. The synthesized MESHEs were confirmed by $^{1}$H NMR (Supplementary Fig. 4) and XPS spectra (Supplementary Fig. 5). We hypothesized that increasing the composition of hydrophobic C–F groups would result in reduced underwater self-healing speed owing to the increased hydrophobic chain density which may limit the amount of water ingress necessary for effective self-healing via boronate ester bond hydrolysis. However, a higher composition of C–F groups is essential to enhance the ion trapping effects,

enabling efficient piezo-ionic dynamics. As hypothesized, the underwater self-healing speed of MESHE3 significantly decreased owing to its high C–F groups composition (Fig. 2c, Main Manuscript). Nevertheless, we also note that NFPU with no C–F group composition exhibited the lowest underwater self-healing speed. This is because NFPU possesses limited hydrophobic chain density, hence, there is excess water ingress that causes excessive hydrolytic dissociation (Supplementary Fig. 6–8 and Supplementary Table 3–5). Thus, MESHE2 was perceived to exhibit the optimum condition for fast underwater self-healing speed and enhanced ion trapping effects, simultaneously. Here, the self-healing speed (μm/min) can be defined as the rate at which the damaged sample achieves maximum self-healing efficiency with or without external stimuli. The self-healing speed is often determined from the ratio of [notch size] (μm) / self-healing time (min)[1,2]. However, in this study, the sample was cut entirely with the knife penetrating through to the lower part, hence, the notch size was determined by the thickness of the sample. Furthermore, the self-healing efficiency was calculated from the ratio of (the toughness of healed sample / the toughness of original sample)[3] x 100%. The formulae are as follows:”

$$\text{Self}-\text{healing speed} = \frac{\text{Thickness}\ (\mu\text{m})}{\text{Self}-\text{healing time}\ (\text{min})} \qquad (1)$$

$$\text{Self}-\text{healing efficiency} = \frac{\text{Toughness}_{\text{healed}}}{\text{Toughness}_{\text{Original}}} \times 100\% \qquad (2)$$

**Supplementary Note 2. Preparation and morphological characterization of molecularly engineered self-healing piezo-ionic elastomer (MESHPIE)**

To explore the optimum concentration of $[BMIM]^+[TFSI]^-$ ionic liquid (IL) for the maximum output sensing performance and higher device sensitivity, various IL concentrations (10–40 wt%) were added to MESHE2 to construct MESHPIE. Firstly, the effect of IL on the hydrophobicity and self-healing speed of the MESHPIE were investigated. As presented in Fig. 2d (Main Manuscript), we observed an increasing hydrophobic tendency as the concentration of IL increased. This is because the IL exhibits hydrophobic properties owing to the delocalization of the negative charge and steric hindrance[4] of $[TFSI]^-$. Meanwhile, the underwater self-healing speed increased slightly only in the MESHPIE@10 wt% but drastically decreased as the IL content increased. This phenomenon can be attributed to the increase in hydrophobic chain density imparted on the MESHPIE by the IL (Fig. 2d, Main Manuscript), with MESHPIE@10 wt% exhibiting the

maximum hydrophobicity for effective underwater self-healing. Furthermore, the ion conductivities of the MESHPIE as a function of various IL contents were investigated (Supplementary Fig. 9). As more IL contents were introduced, the conductivities increased owing to the highly available free mobile ions. The increased presence of mobile ions was verified via X-ray powder diffraction (XRD) curves. As depicted in Supplementary Fig. 10, the XRD peaks revealed the amorphous nature of the MESHPIEs with a hike in the intensity of low angle ($2\theta$ = 12.4°), more prominent in MESHPIE 20–40 wt%, owing to the intercalated ions between the PU hard segments[5]. In addition, the plasticization effects of ions yielded a lower Young's modulus and higher elongation at break in the MESHPIE as the IL content increased (Supplementary Fig. 11 and Supplementary Table 6). Next, the pressure dependent capacitance changes of the MESHPIE were also explored by analyzing the initial and final capacitance values as a function of the IL content under external pressure (0~95 kPa). As illustrated in Supplementary Fig. 12, MESHPIE@30 wt% exhibited the highest $C_p/C_0$ for the maximum output sensitivity. Hence, MESHPIE@30 wt% was preferred as the optimum condition for further device analysis (hereof treated as MESHPIE).

To examine the reversible compressibility of MESHPIE for effective device performance, compressive stress-strain tests were performed with cyclic loading/unloading strain (Supplementary Fig. 13). The compressive test exhibited similar loading/unloading curves emphasizing the stability and outstanding fatigue resistance of MESHPIE, indicative of its remarkable elastic recovery capabilities (Supplementary Fig. 13a). Additionally, we subjected the material to 100 consecutive compression cycles to further evaluate its fatigue resistance (Supplementary Fig. 13b). Following 100 cycles of compression tests, the stress attenuation registered a mere 1.93%, attesting to the material's outstanding fatigue resistance. Furthermore, the mechanical self-healing properties of MESHPIE in air and underwater were analyzed at different time intervals via stress-strain curves (Fig. 2g Main Manuscript, Supplementary Fig. 14, and Supplementary Tables 7–8). MESHPIE exhibited self-healing efficiencies of 94.5% after 50 minutes and 89.6% after 40 minutes in air and underwater, respectively. Furthermore, the self-healing performance was characterized by rheological oscillation strain tests. Supplementary Figs. 15a and 15b present the rheological oscillation strain sweep tests of MESHPIE in air and with water, respectively. As the strain amplitude increased, the storage modulus (G′) decreased more significantly even below the loss modulus (G″), indicating a disruption of the internal structures

beyond 15.9% strain in air and 12.5% strain with water. To verify the self-healing of the internal structures, rheological time-sweeping experiments were also performed by alternatively applying repeated strains of 0.3% and 50%. MESHPIE in both air (Supplementary Fig. 15c) and with water (Fig. 2h) displayed excellent recovery of G′ and G″ after reducing the strain from 50% to 0.3%, indicating the reconstruction of the internal network structures. The repeated damage-recovery tests demonstrated the robust recovery of MESHPIE to its original state without compromising its mechanical properties. Most importantly MESHPIE exhibited superior self-healing speed in both air and underwater, as compared with other reported room temperature self-healing dielectric elastomers and ionic-based materials with various healing mechanisms, such as hydrogen bonds, disulfide bonds, electrostatic (ion-dipole) interactions, imine bonds, Diels-alder bond, and their combinations (references are in Supplementary Table 9).

**Supplementary Note 3. Characterization of ion-dipole interaction.**

The IL interacts with the MESHPIE polymer chains via ion-dipole interactions. The C–F-induced dipoles of MESHPIE polymer chains attract the $[BMIM]^+$ cations in the IL leading to the trapping of $[BMIM]^+[TFSI]^-$ ion pairs, which initiates the piezo-ionic dynamics (trap and release mechanism). To confirm the presence of ion-dipole interactions, firstly, the internal molecular structures of IL, MESHE, and MESHPIE were characterized via FTIR (Fig. 3a–e, Main Manuscript). The shifting of spectra bands toward higher wavenumber indicates the weakening of the Coulomb interactions between the IL pairs owing to the dragging away of the cations by the C–F dipoles[6-9]. In addition, Visual Molecular Dynamics (VMD) program was used to plot the color-filled iso-surface graphs (Fig. 3f) to visualize the molecular electrostatic potential (MESP) of C–F and ions to predict the possible interaction sites. The MESP values on the MESHPIE molecular surfaces can be visualized by the red and blue colors surrounding the molecules, where the red indicates positive MESP value and blue indicates negative value. Notably, the C–F groups exhibit negative electrostatic potential on the surface; hence, the blue regions are more likely to interact with the ions to form ion-dipole interactions.

Furthermore, four different structures of the complexes between the C–F groups (i.e., PFDT) and $[BMIM]^+[TFSI]^-$ ion pairs were built and fully optimized at the B3LYP-D3BJ/def2-SVP level of theory to compare their relative stability (Fig. 3g and Supplementary Fig. 16a–c). A full geometry optimization revealed the most conformal stability at an attractive binding energy of

-13.803 kcal/mol. Additionally, independent gradient model (IGM) analysis was carried out for the most stable structure among the complexes by using Multiwfn software to deeply understand the nature of intermolecular interactions (Fig. 3h, Main Manuscript). IGM strongly confirms the non-covalent interactions (green area) between the C–F groups and $[BMIM]^+[TFSI]^-$ ions caused by the ion-dipole interactions. The extent to which the C–F groups contribute to this non-covalent interaction can be indicated by color bands, where dark red indicates higher participation in the interaction, and darker blue indicates lower participation. These experimental results also strongly reaffirm the existence of ion-dipole interactions between the C–F groups and $[BMIM]^+$ cations.

**Supplementary Note 4. Characterization of piezo-ionic dynamics**

To provide evidence to support the concept that the ion pairs are trapped to the C–F groups, we investigated the relationship between piezo-ionic dynamics and their changes in complex impedance behavior. This was accomplished by conducting stress relaxation tests on both MESHPIE and NFPU-IL (no C–F groups, used as reference), under applied pressure. As external pressure is applied to MESHPIE-based device, the free ion concentration ($c_i$) is expected to increase with the release of trapped ions. For a capacitive process, ion migration to the electrolyte/electrode interfaces occurs under the application of alternating current (AC) bias, generating interfacial electrode polarization[10,11]. Analysis of electrode polarization can be used to explore the $c_i$ in the electrical double layer (EDL) formation. This phenomenon impacts the ion dynamics associated with $c_i$, specifically, the ionic conductivity ($\sigma = {q_i}^2 c_i D_i / kT$, where $q_i$, $D_i$ and $kT$ are the ion charge, ion diffusivity, and temperature, respectively) and Debye screening length ($\kappa^{-1} = \sqrt{\varepsilon_r \varepsilon_0 kT / {q_i}^2 c_i}$, where $\varepsilon_r$ is the dielectric constant and $\varepsilon_0$ is space-charge permittivity)[10,12]. The accumulated total $c_i$ near the electrode characterizes the range to which ionic clouds screen the charged surfaces in the diffusion layer, which influences the Debye screening length[10,11]. Thus, the impedance of MESHPIE-based device is expected to gradually decrease with increasing pressure (Supplementary Fig. 17a), owing to release of more free ions that were initially trapped, leading to an increase in the $c_i$ (also known as ionic strength). Meanwhile, the impedance of NFPU-IL (without C–F groups)-based device exhibited no substantial changes under applied pressure (Supplementary Fig. 17b), as no ion trapping occurred at the initial state, thus pressure-mediated ion pumping effect was not expected under external pressure. Moreover, to confirm that

the decrease in impedance is attributed to the $c_i$ increase, we explored the changes in $k^{-1}$ under applied pressure. In the frequency-dependent real impedance (Z′) and imaginary impedance (Z″) spectra, the frequency at cross-section positions of Z′ and Z″ corresponds to the charge relaxation frequency ($\tau^{-1}$) in the Bode plot[11,13]. Notably, as the applied pressure increased, the defined $\tau^{-1}$ exhibited a shift towards higher frequencies (Fig. 4c, Main Manuscript) owing to faster ionic atmosphere relaxation indicating increased $c_i$ in MESHPIE, which was not an observed tendency in NFPU-IL (Supplementary Fig. 17c).

## Supplementary Note 5. Pressure response under different voltages and various frequencies

Supplementary Fig. 19 and Supplementary Fig. 20 present the pressure responses of various MESHPIE (10–40 wt%)-based devices under different bias voltages (1 mV, 100 mV, and 1 V) and various bias frequencies (1 kHz, 100 Hz, 20 Hz), exhibiting similar pattern of changes in capacitance. This pattern can be attributed to the corresponding increase in device capacitance with increasing pressure, owing to the pressure-induced breaking of ion-dipole interactions, which subsequently results in the release of more free mobile ions. This enhances EDL formation at the MESHPIE/electrode interface. In addition, the influence of the applied bias voltage on the observed increasing tendencies of both the initial capacitance ($C_0$) and final capacitance ($C_p$, ~95 kPa), suggests that the intensified electric field across the MESHPIE plays a role in the device capacitance. This phenomenon can be explained by the weakening of ion-dipole interactions as the applied voltage bias increases[14]. Consequently, there is a voltage-induced migration of ions toward the respective electrodes, thereby enhancing EDL formation. The observed pressure response under various bias voltages provides compelling evidence to support our claim that the pressure sensing mechanism in MESHPIE can be attributed to the piezo-ionic dynamics initiated by the C–F groups.

## Supplementary Note 6. The concept behind the increase in LED intensity upon impact or under pressure

The increase in LED intensity upon impact or under applied pressure can be explained based on two concepts. Firstly, in an alternating current (AC) circuit, the current flow is characterized by the capacitive reactance $/Z/ = 1/\omega C$, where $\omega$ denotes the angular frequency of the AC power supply and C denotes the capacitance of the device. Thus, the capacitive coupling of the AC

voltage drives the flow of charges across the LED[6,15]. Secondly, MESHPIE functions as an ionic conductive medium, generating electrode polarization at the MESHPIE/electrode interface under an applied AC voltage. This polarization enables the flow of charges through the device. Initially, only a few mobile ions were available for charge to flow. However, upon impact or under pressure, the ion pumping phenomenon in MESHPIE induces the release of additional free mobile ions. This phenomenon reduces the overall bulk resistance, facilitating the flow of more charges to the LED, thereby increasing its intensity.

Furthermore, to support the underwater electrical performance of MESHPIE, capacitance (Supplementary Fig. 22a) and impedance (Supplementary Fig. 22b) tests were conducted in both ambient and underwater conditions. The results showcase the exceptional underwater electrical performance and stability of MESHPIE, ensuring the stable underwater application of MESHPIE-based devices. Additionally, the MESHPIE-based device was submerged in water for a period of 10 days to support its stability in underwater condition over a longer period. As illustrated in Supplementary Fig. 23a, the device exhibited a slight increase in mass after 1 day of immersion owing to the ingress of small quantities of water. The presence of excellent hydrophobic interactions effectively repelled the majority of interfacial water molecules, resulting in minimal water absorption. Subsequently, over the remaining 9 days, the device's mass remained virtually unchanged, indicating outstanding stability. Moreover, the nearly overlapping impedance curves of the device immersed in water for the different days (Supplementary Fig. 23b), also emphasize the stable underwater electrical performance of our device, reaffirming its significant potential for underwater applications.

The electrical self-healing is achieved in the device owing to the tractive force generated by the self-healing driving force of both MESHPIE and the underlying PU substrate (MESHE2; refer to the methods section for the detailed information on electrode fabrication), which facilitates the reestablishment of the percolation network of Ag nanowires (AgNWs)[6,16,17]. When the device was cut through with a sharp blade, the AgNWs became electrically insulated from each other, resulting in a complete loss of LED brightness. The brightness was restored within 1.26 min in air (Supplementary Movie 2) and 0.33 min underwater (Supplementary Movie 6), indicating effective self-healing properties. The healing process is attributed to both the self-healing of the PU films and the fusion of AgNWs, which reconnect the percolation network across the cracked surfaces[17]. It should be noted that if the cut device pieces were completely separated, the efficiency of

electrical self-healing would significantly decrease, because achieving a perfect alignment of the AgNWs networks is nearly impossible, and this alignment strongly affects the restoration of the conductive surfaces.

Supplementary Figures

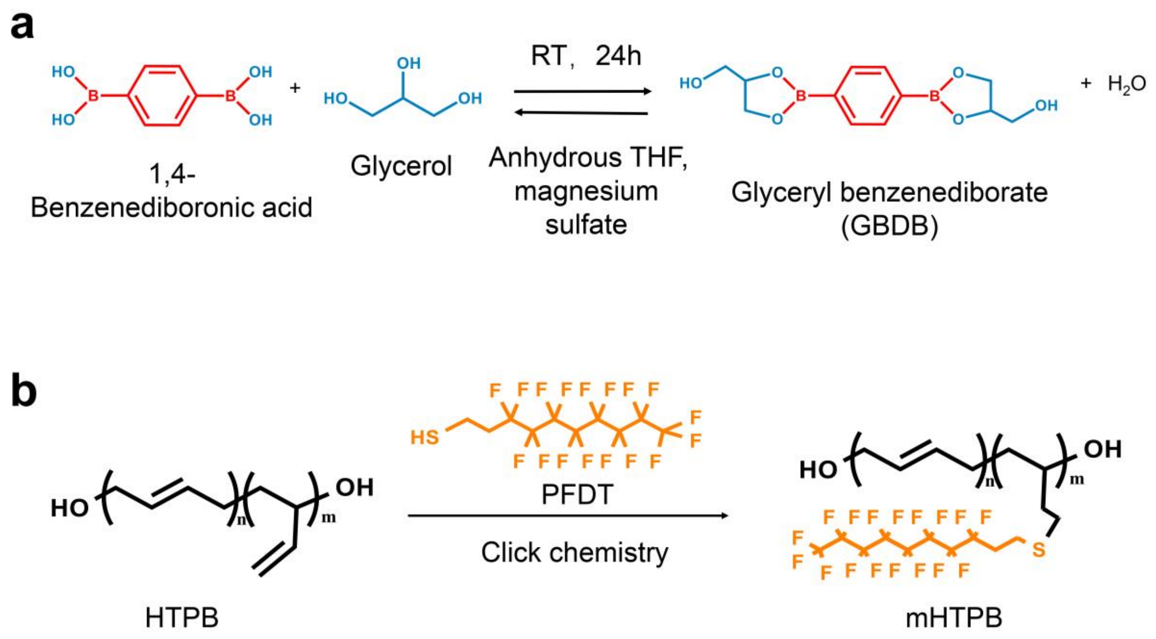


**Supplementary Fig. 1 | Synthesis of chain extender and soft segment.** Schematics illustrating the synthesis of **a,** glyceryl benzenediborate (GBDB) chain extender and **b,** C–F groups-functionalized mHTPB.

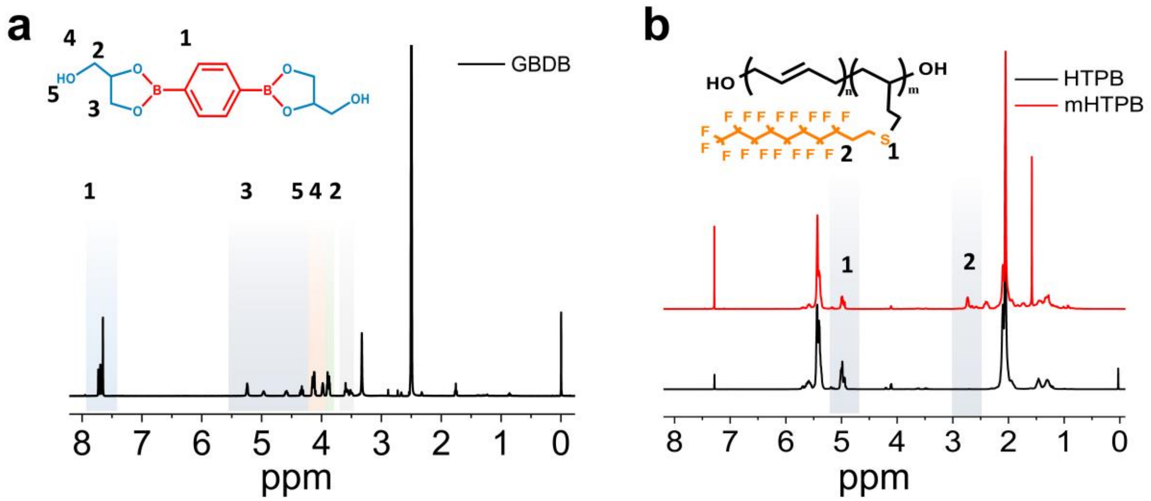


**Supplementary Fig. 2 | Verification of synthesized Materials.** $^{1}$H-Nuclear magnetic resonance spectroscopy ($^{1}$H NMR) images of **a,** GBDB chain extender and **b,** soft segments

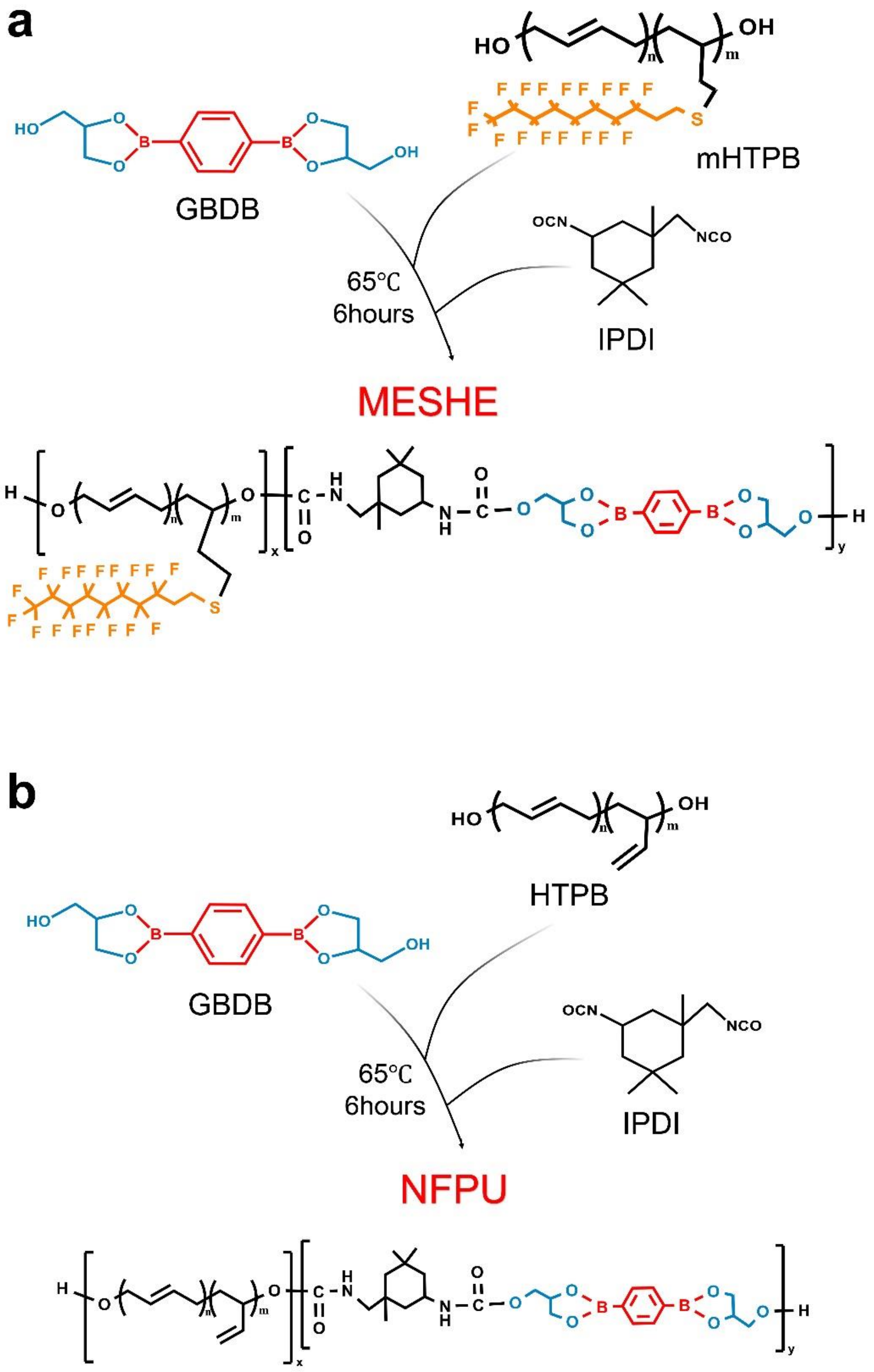


**Supplementary Fig. 3 | Synthetic route of our polyurethanes.** Schematics illustrating the one-step polymerization synthesis of **a,** molecularly engineered self-healing elastomer (MESHE) and **b,** no C–F groups polyurethane (NFPU) (used as reference).

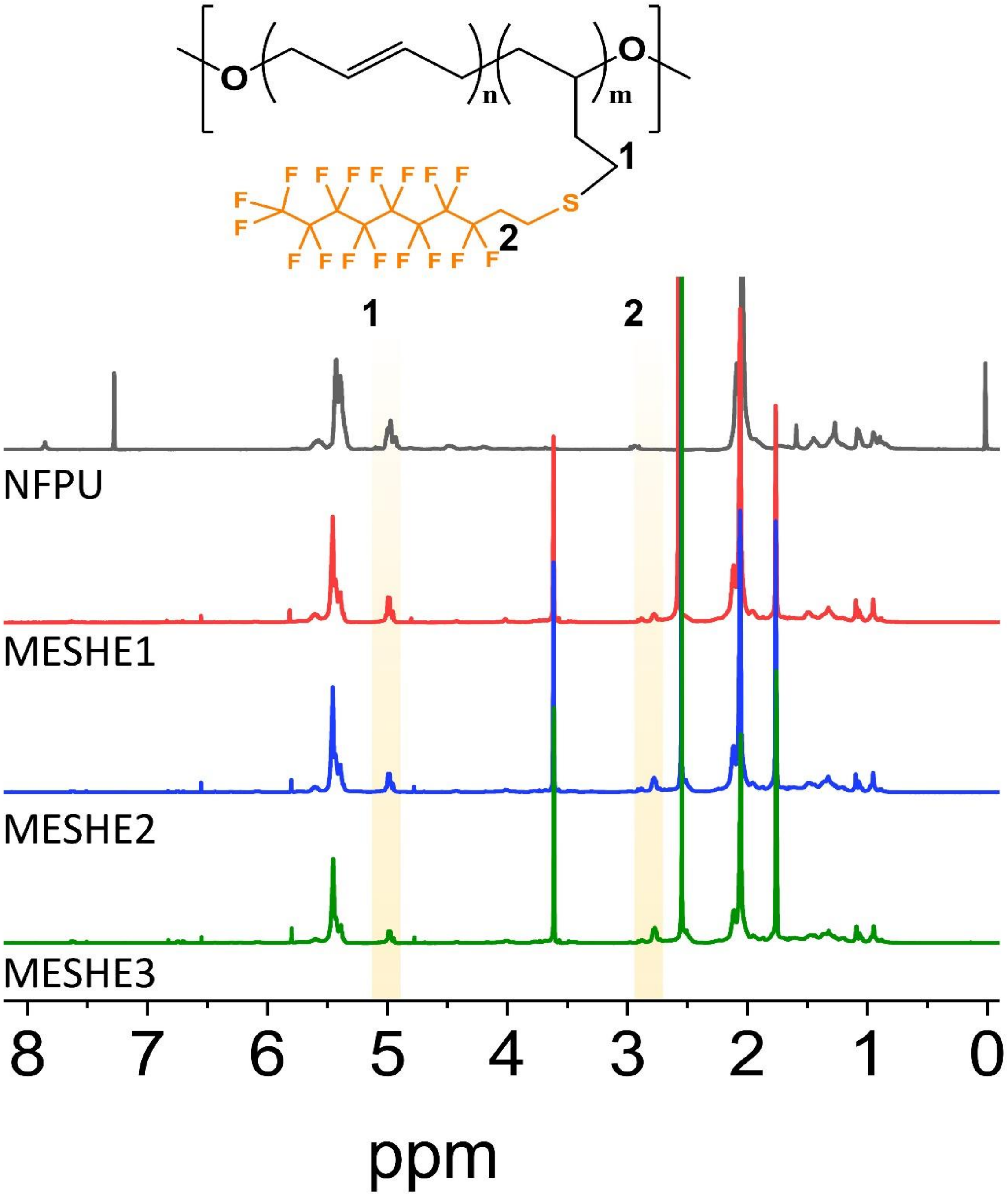


**Supplementary Fig. 4 | $^{1}$H NMR results**. $^{1}$H NMR characterization of the various PUs.

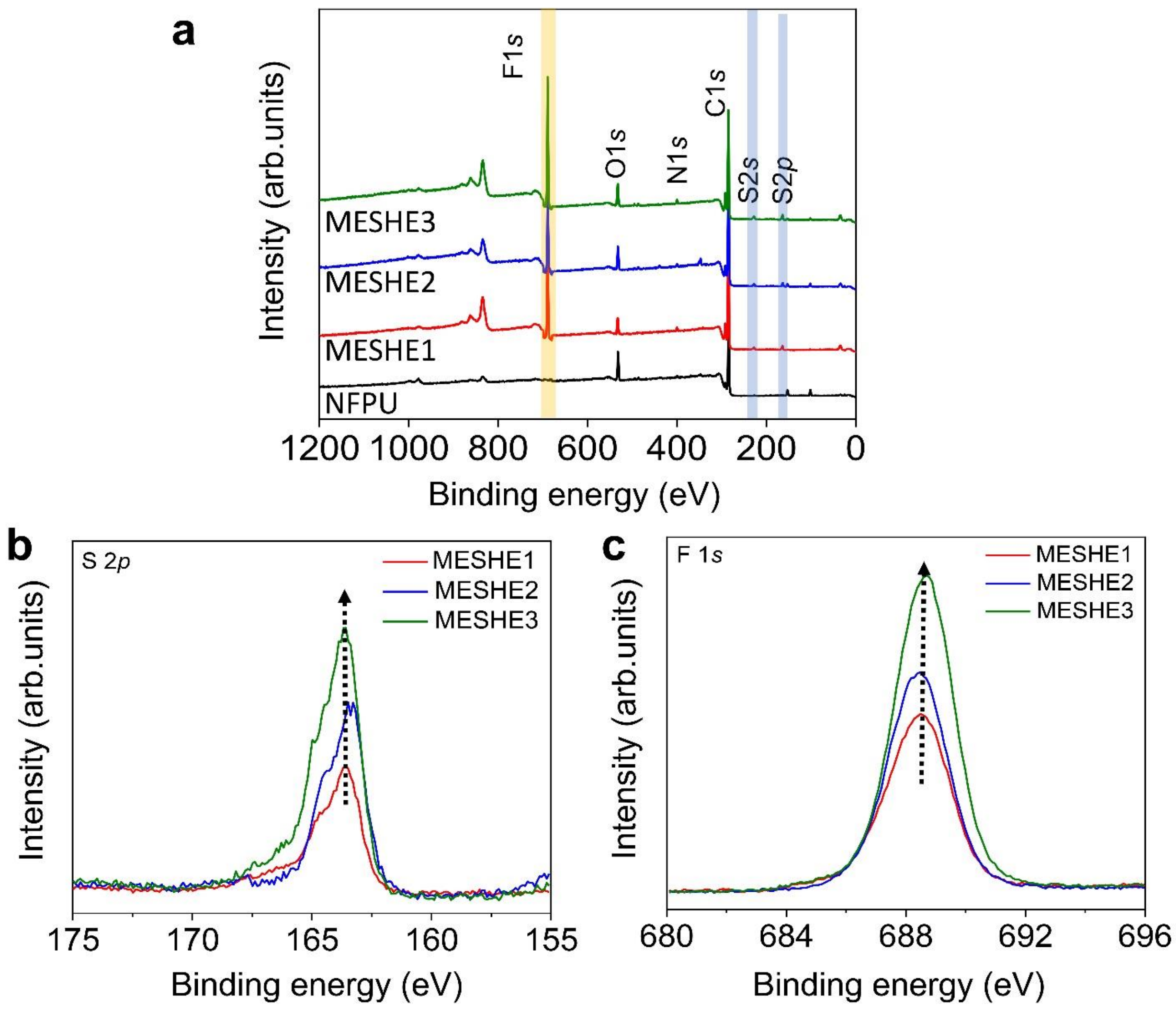


**Supplementary Fig. 5 | XPS analysis.** XPS characterization of PUs **a,** full range spectra of the MESHEs. **b,** S 2*p* spectra and **c,** F 1*s* spectra of the MESHEs.

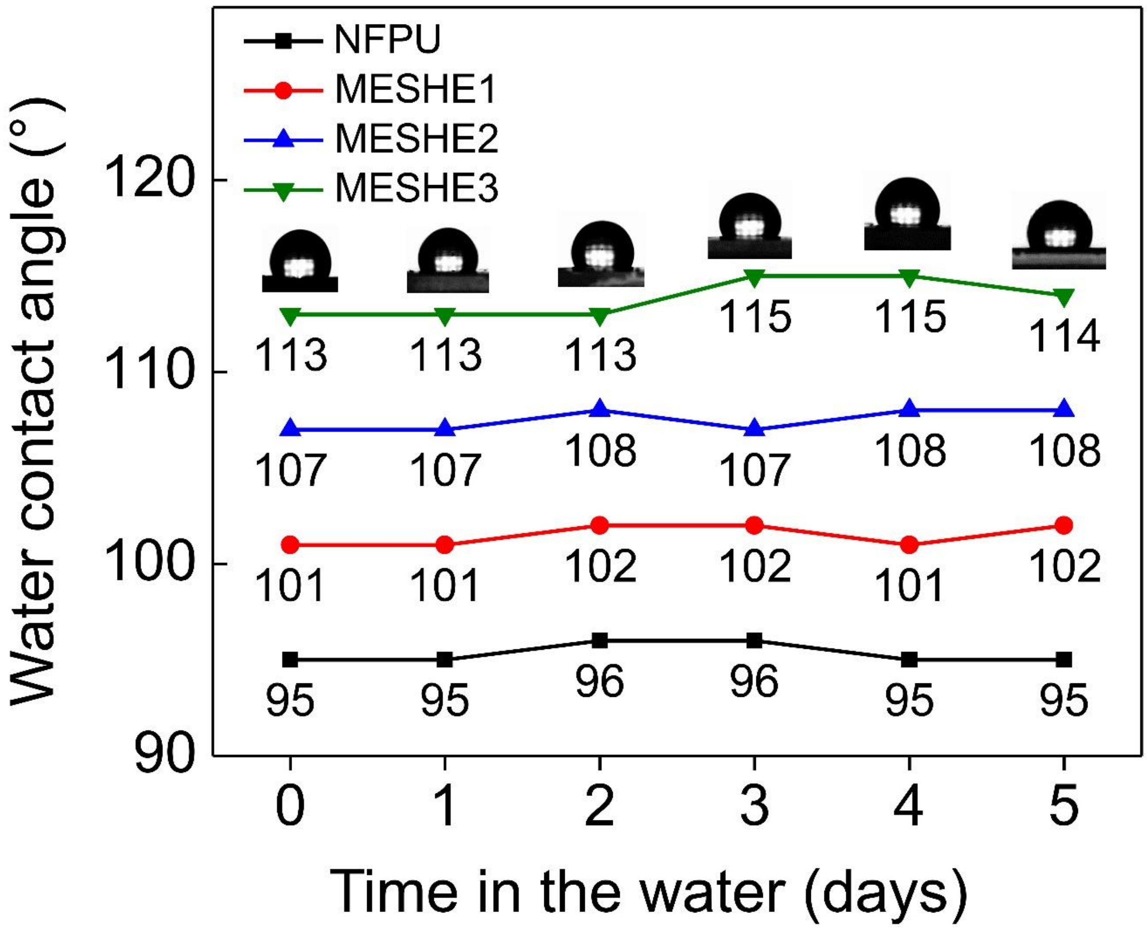


**Supplementary Fig. 6 | Changes of water contact angles.** Images of the water contact angles of the various PUs and calculated angles representing the changes in their hydrophobicity after being immersed in water for five consecutive days.

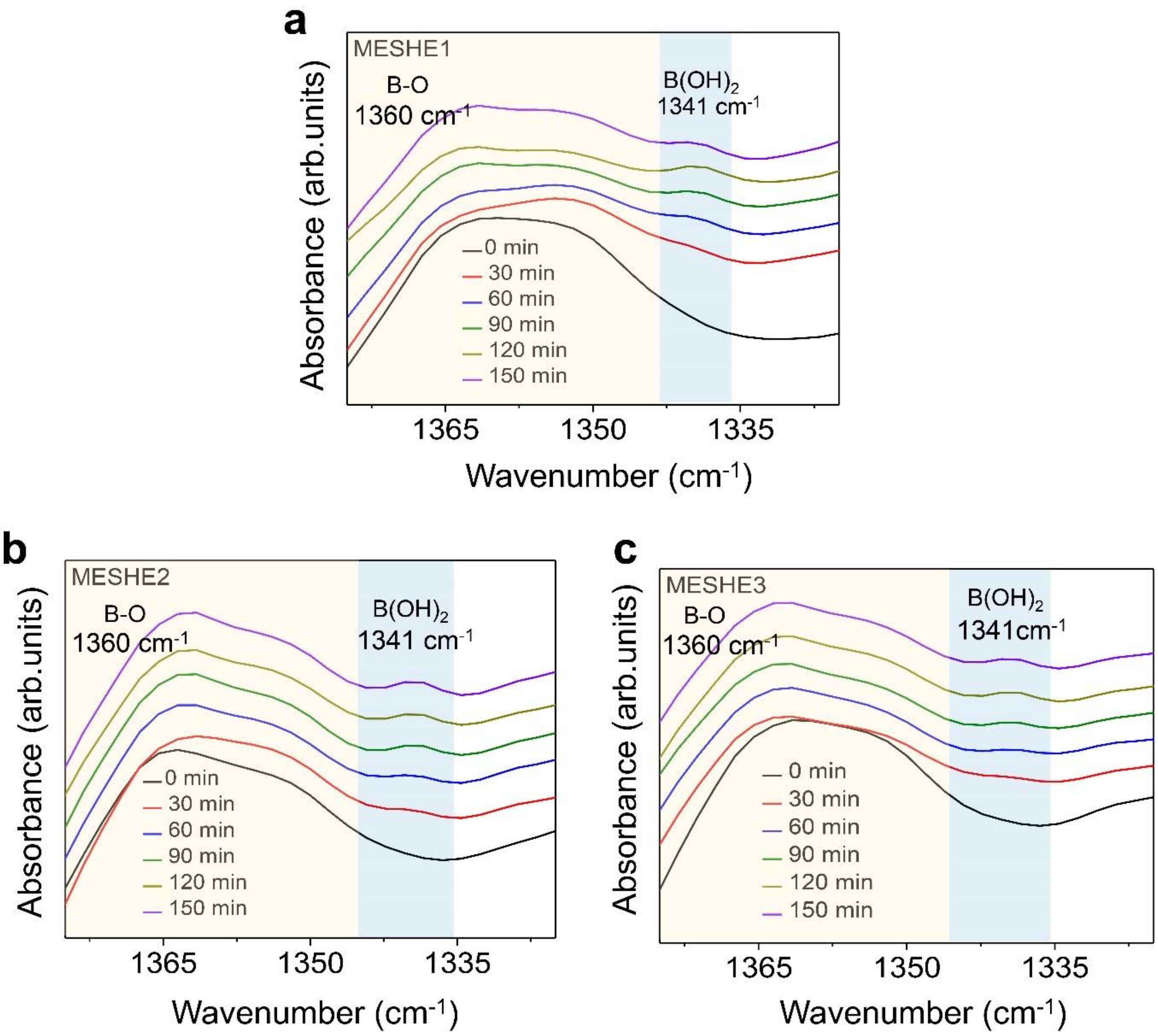


**Supplementary Fig. 7 | ATR–FTIR spectra of a,** MESHE1 **b,** MESHE2 and **c,** MESHE3 in the spectral regions of 1380–1335 $cm^{-1}$ attributed to B-O stretching at different water immersion times.

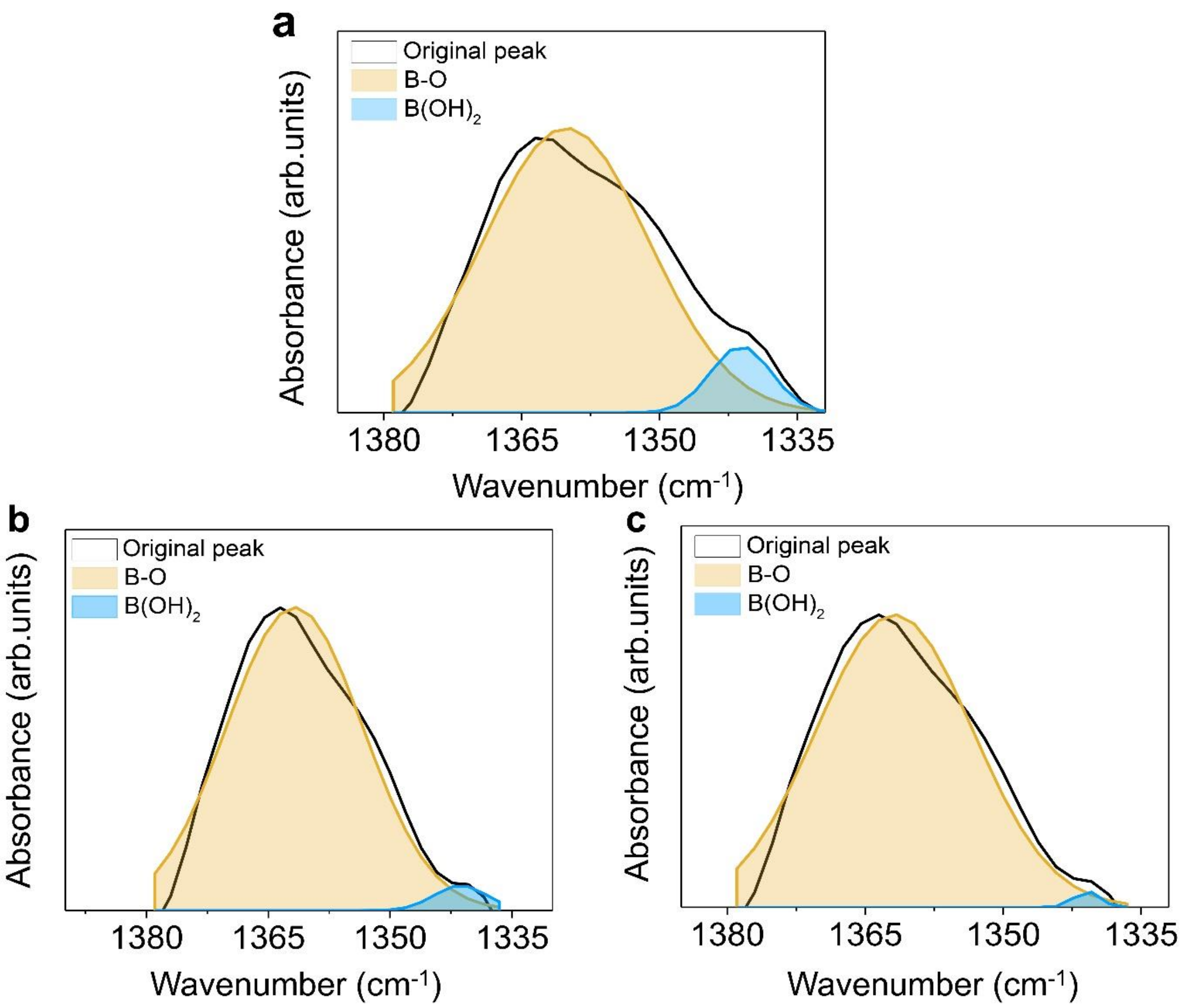


**Supplementary Fig. 8 | Deconvoluted ATR–FTIR spectra peaks** in the spectral regions 1380–1335 cm$^{-1}$ (B-O stretching) of **a,** MESHE1, **b,** MESHE2 and **c,** MESHE3 showing the water intake of the MESHEs after 150 min.

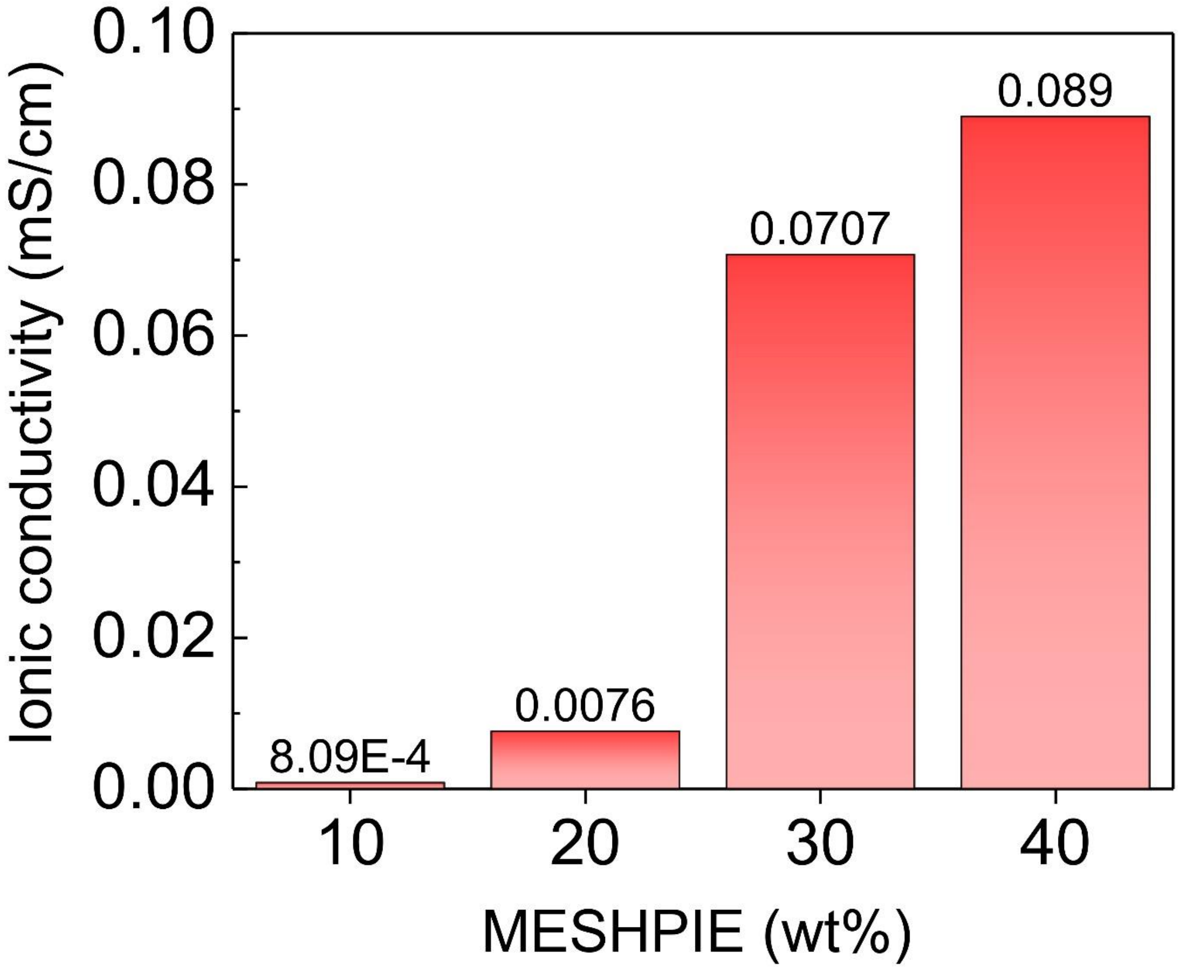


**Supplementary Fig. 9 | Ionic conductivity vs ion concentration.** Ionic conductivity plots of various MESHPIEs under applied external pressure obtained from electrochemical impedance spectroscopy (EIS) data.

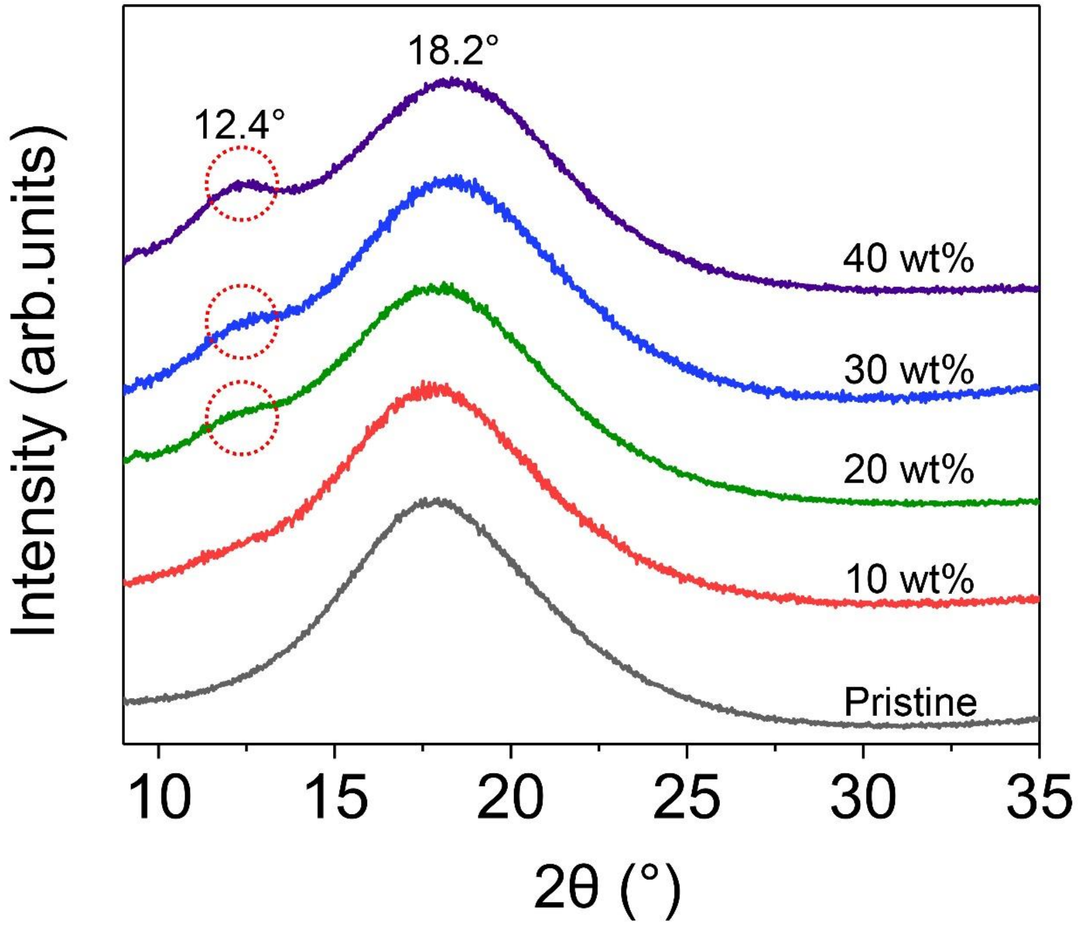


**Supplementary Fig. 10 | XRD plots of MESHPIEs** displaying the pattern of varying IL concentration. A hike in intensity at a low angle indicates intercalation of ions in the hard segment.

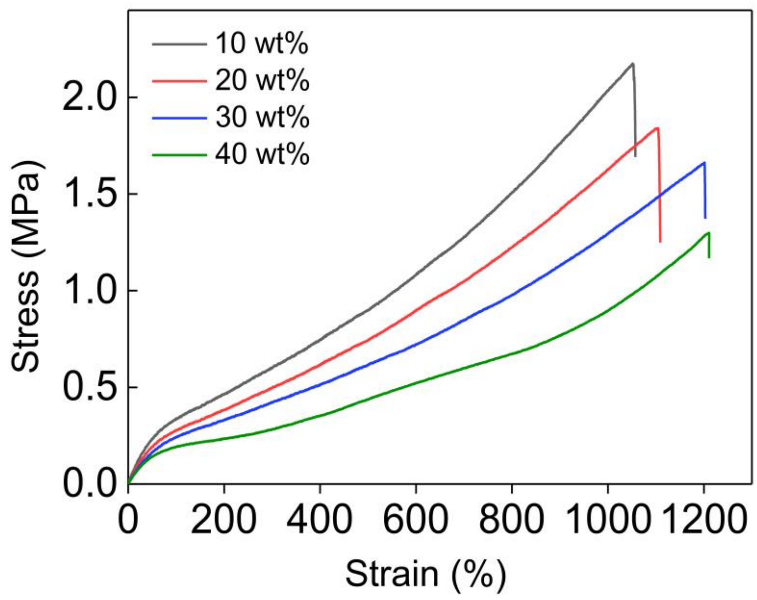


**Supplementary Fig. 11 | Stress–strain curves** of various MESHPIEs (10-40 wt%) exhibiting decreased modulus and increased elongation at break as IL concentration increases owing to plasticization effects of ions.

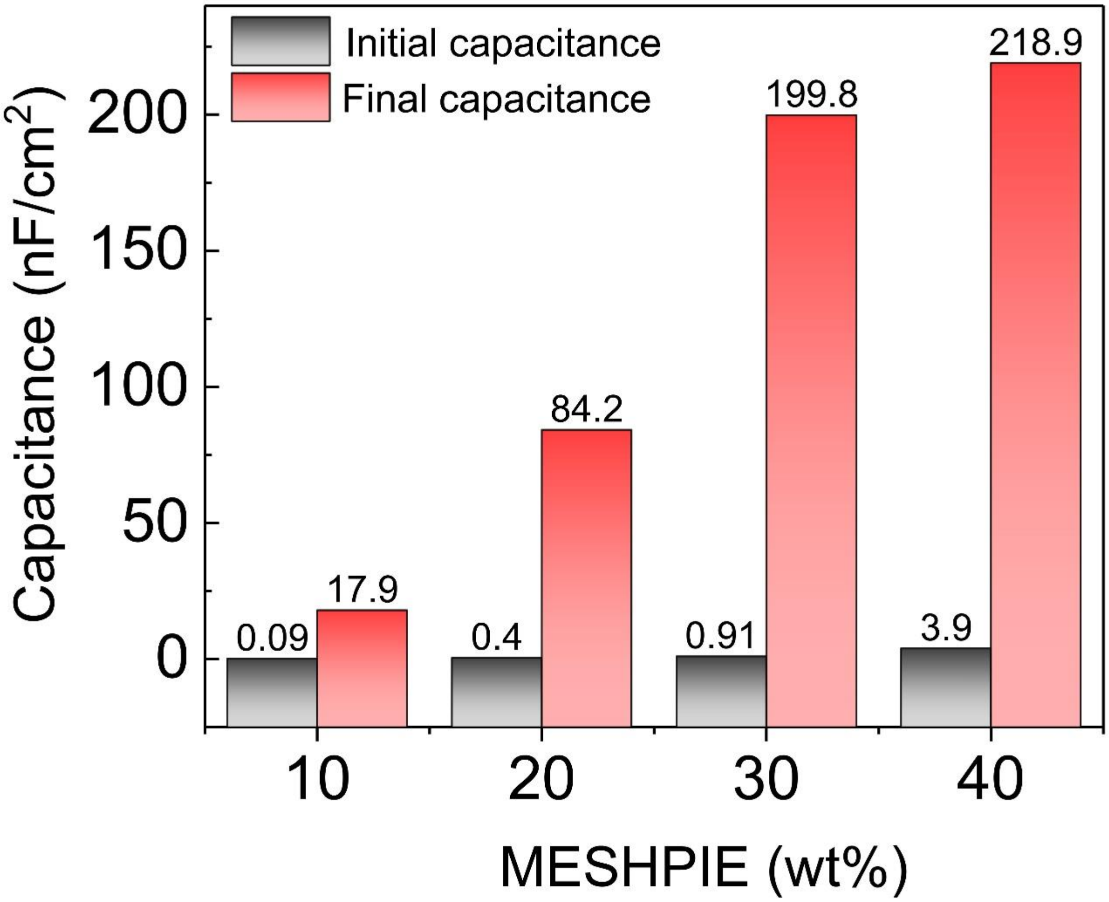


**Supplementary Fig. 12 | Pressure-dependent capacitance changes.** The initial and final capacitance values of various MESHPIE (10-40 wt%)-based devices under applied pressure range of ~95 kPa (100 mV bias voltage at 20 Hz).

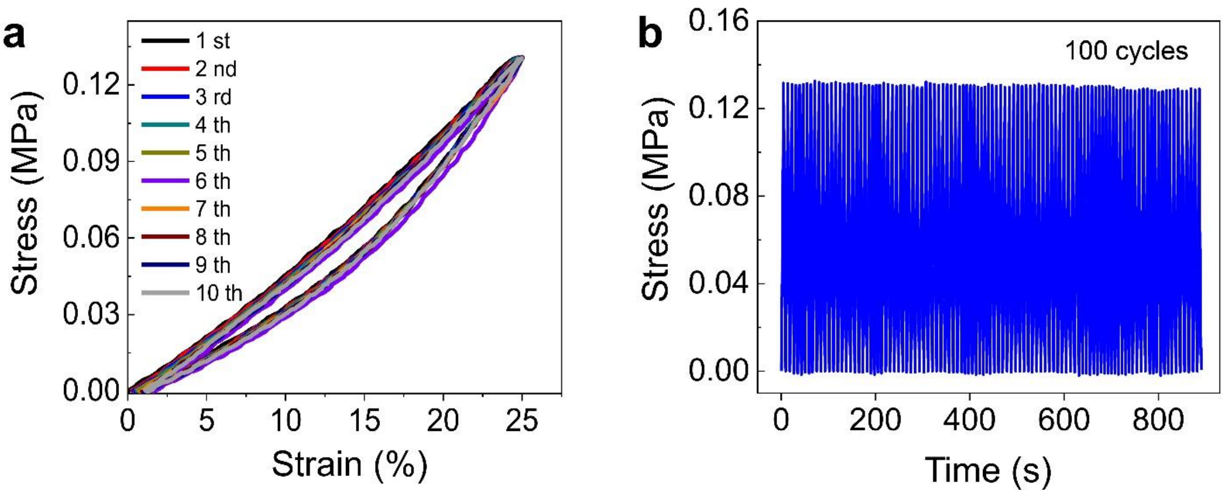


**Supplementary Fig. 13 | a Sequential cyclic compressive stress-strain** curves of MESHPIE at 25% strain. **b** Fatigue resistance test of MESHPIE by sequential cyclic compression test (100 times).

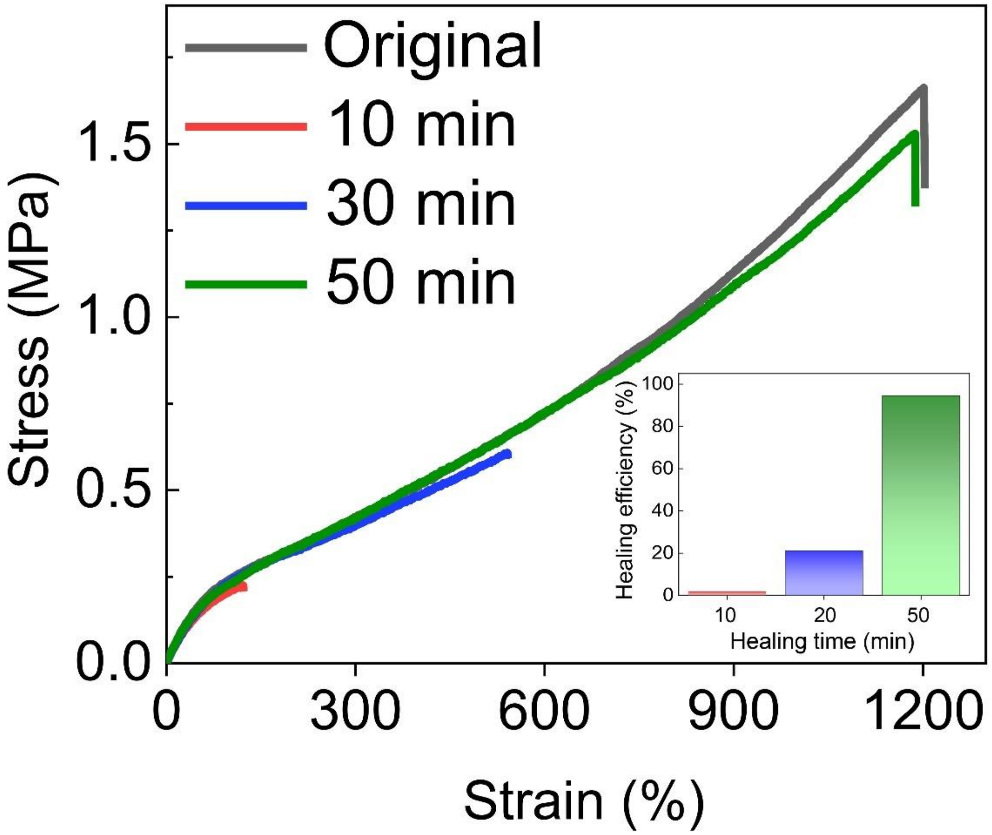


**Supplementary Fig. 14 | Stress–strain curves at various healing times in air**. Recovery of mechanical properties of self-healed MESHPIE at various healing times in air. Insert is a bar graph depicting the self-healing efficiency of MESHPIE in air.

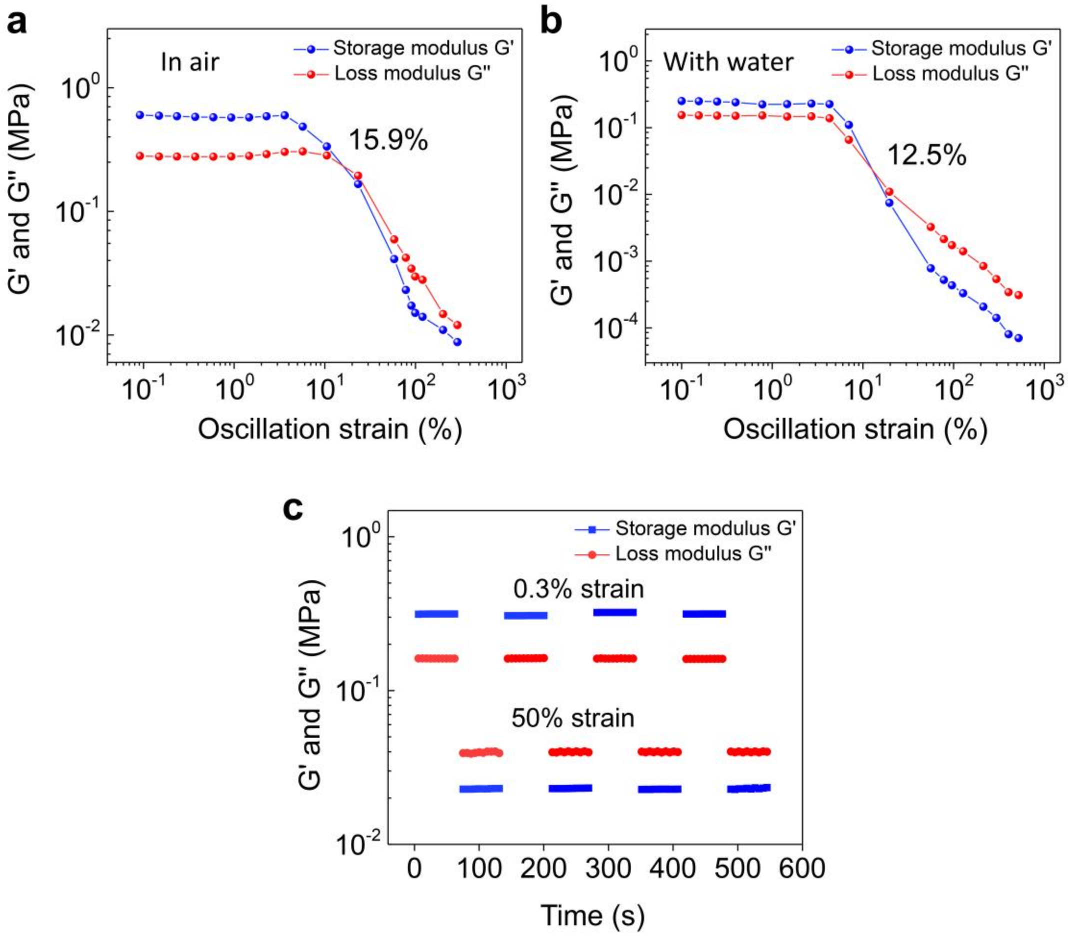


**Supplementary Fig. 15 | Rheological measurements.** Oscillatory strain sweeps of MESHPIE **a,** in air and **b,** with water at 25 ºC. **c,** Oscillatory time sweep of MESHPIE in air.

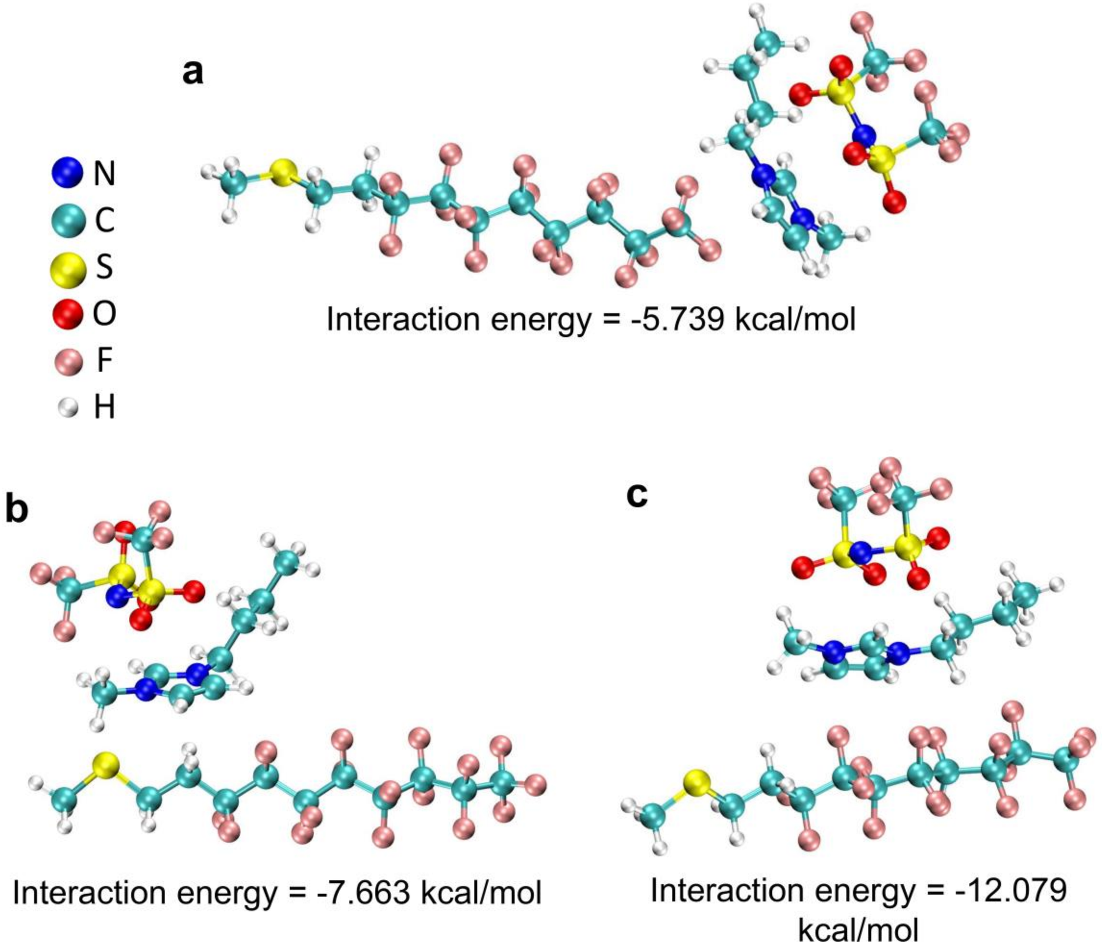


**Supplementary Fig. 16 | a-c**, Complexation energies via quantum chemical computing of imidazolium cation $[BMIM]^+$ in the presence of counter anion $[TFSI]^-$ with C–F groups for three different structures of the complex.

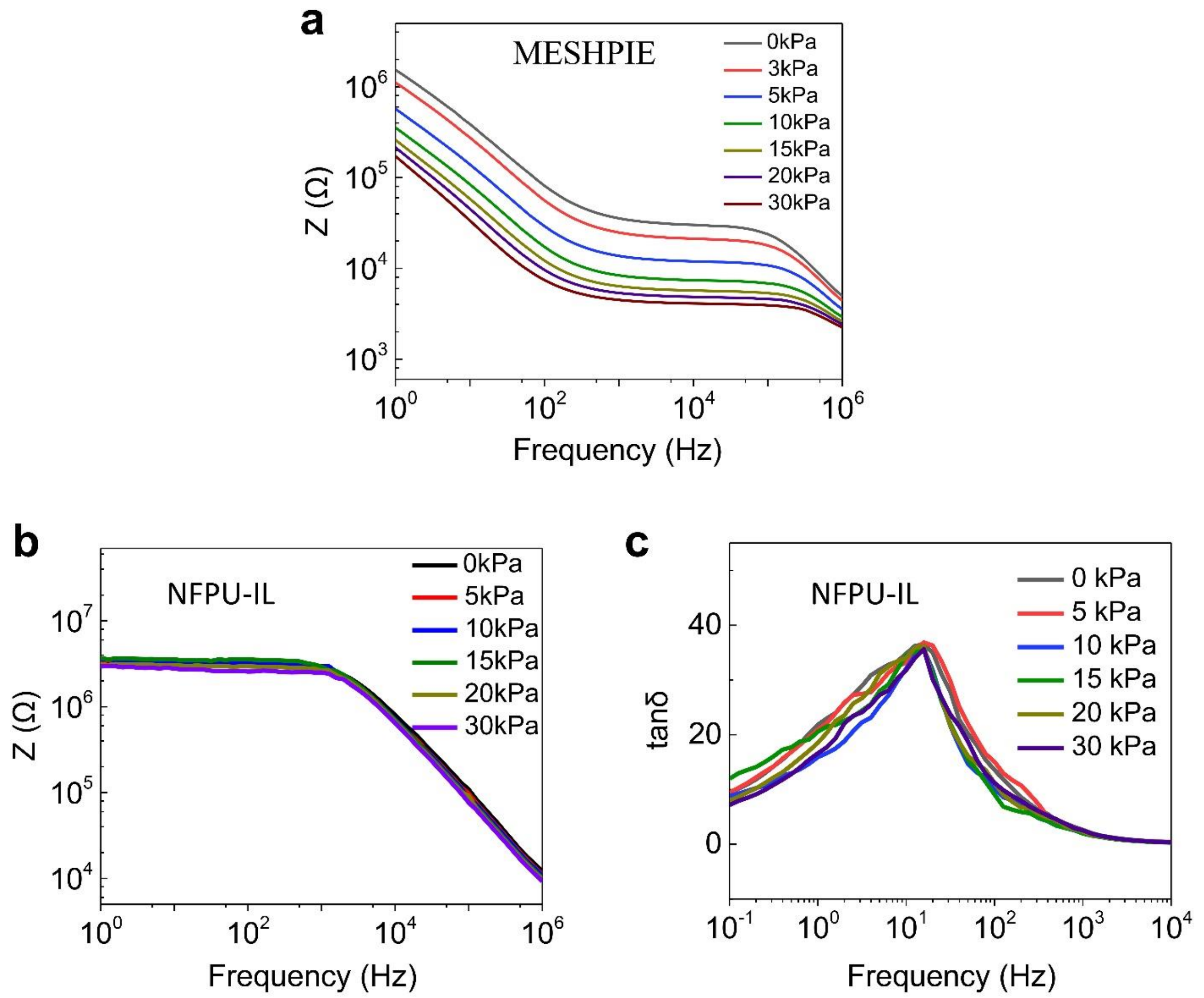


**Supplementary Fig. 17 | Piezo-ionic (trap and release) effect.** Relationship between piezo-ionic dynamics and the changes in complex impedance behavior as a function of applied external pressure. **a,** MESHPIE and **b,** NFPU-IL. **c,** Ion dynamics and free ion concentration of NFPU-IL with a stepwise pressure increase.

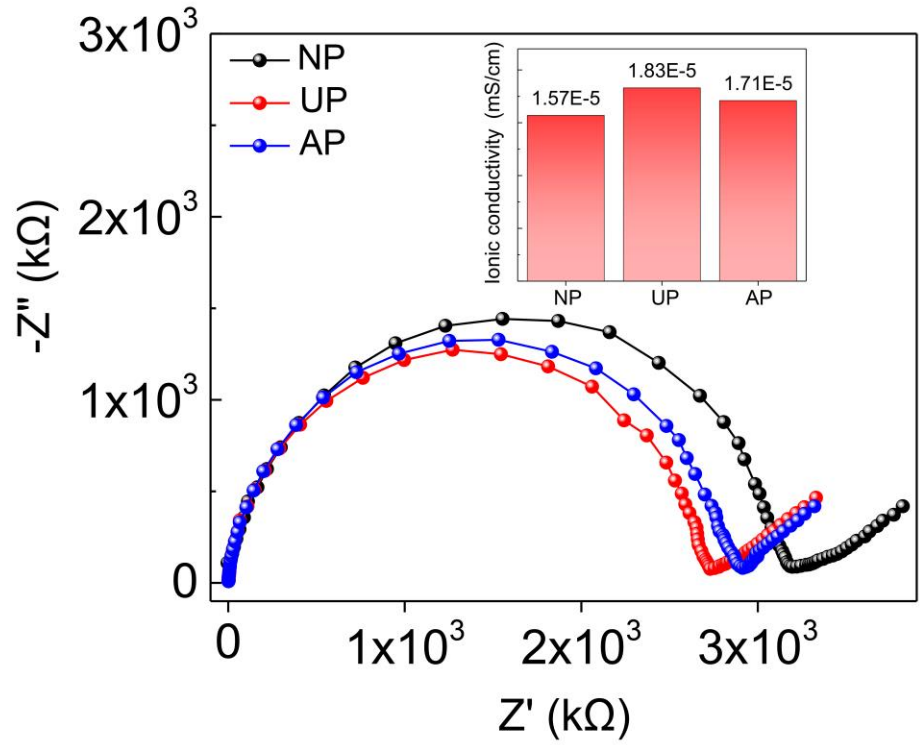


**Supplementary Fig. 18 | Nyquist plot of NFPU-IL.** Ionic conductivity of NFPU-IL under no pressure (NP), under pressure (UP), and after removing pressure (AP) (insert displays ionic conductivity under each condition).

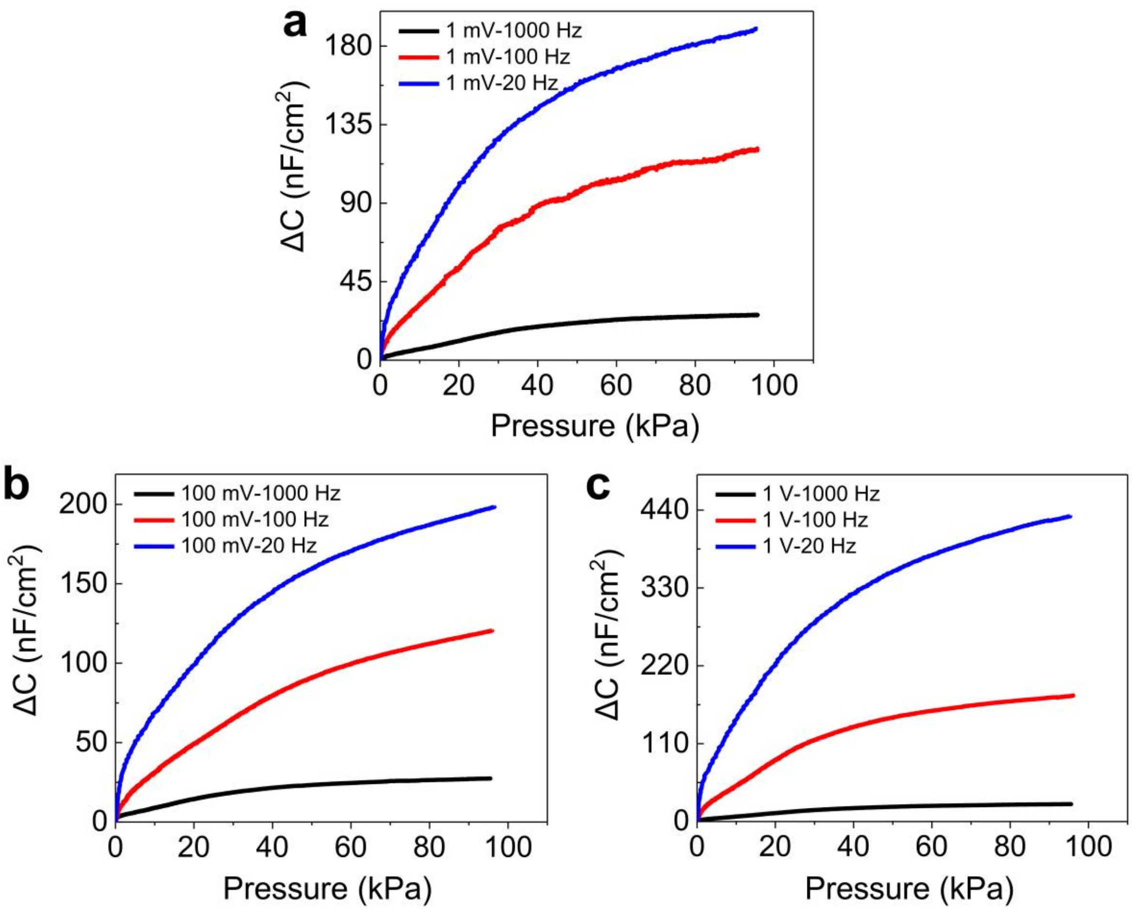


**Supplementary Fig. 19 | Pressure responses of MESHPIE under different conditions**. The pressure-dependent responses of the MESHPIE (30 wt%)-based device at applied bias voltages of **a**, 1 mV, **b**, 100 mV and **c**, 1 V as a function of different frequencies (1 kHz, 100 Hz, and 20 Hz).

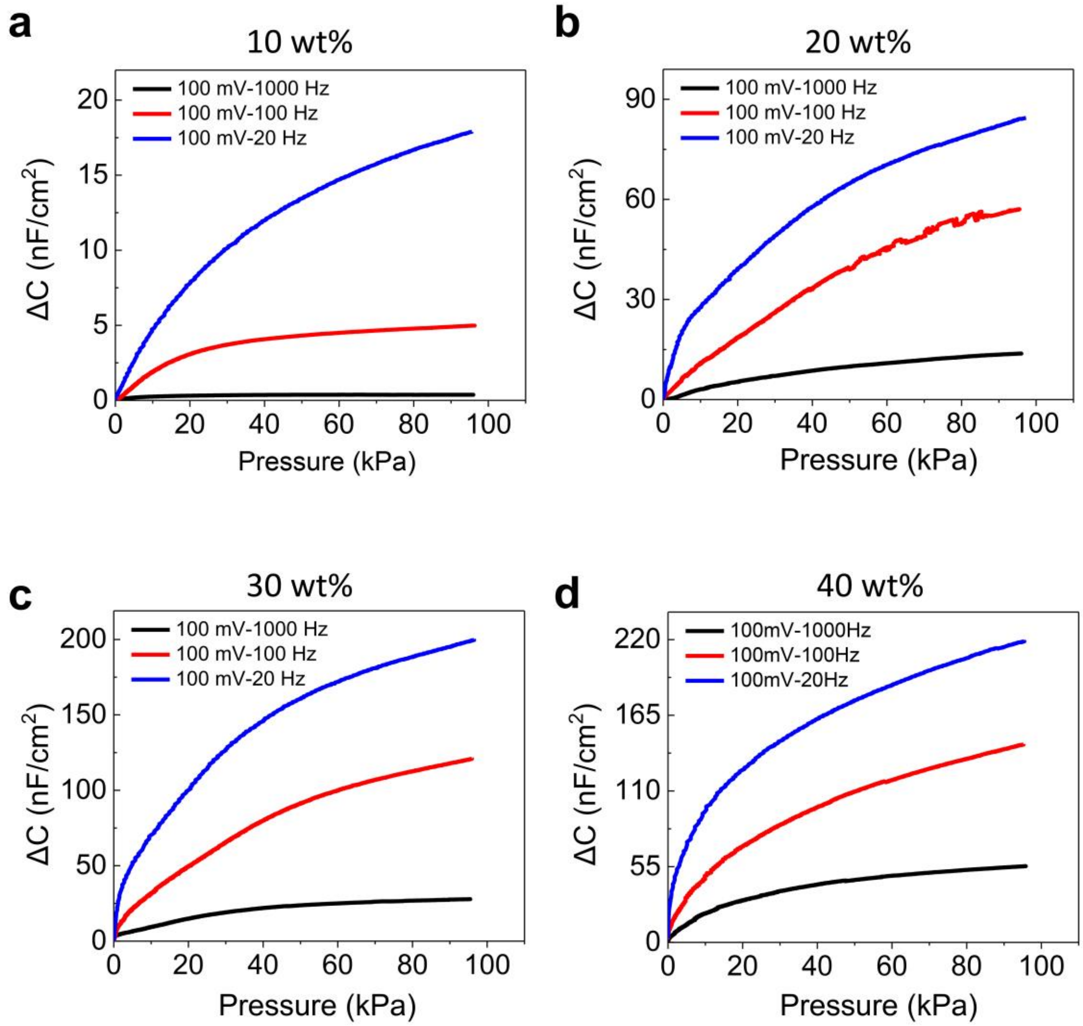


**Supplementary Fig. 20 | Pressure responses of MESHPIEs under different conditions**. The pressure-dependent responses of **a**, MESHPIE@10wt%, **b**, MESHPIE@20wt%, **c**, MESHPIE@30wt%, and **d**, MESHPIE@40wt%-based devices at 100 mV applied bias voltage as a function of different frequencies (1 kHz, 100 Hz, and 20 Hz).

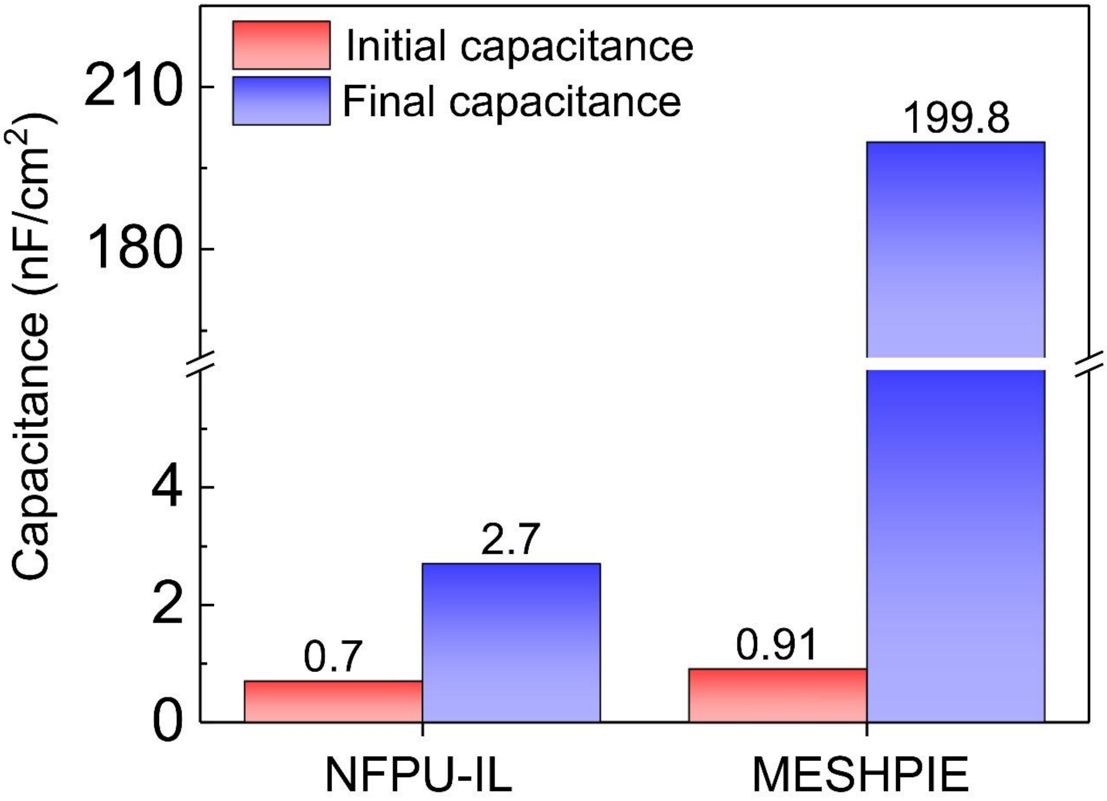


**Supplementary Fig. 21 | Influence of C–F groups on the overall capacitance.** Representation of initial (without pressure) and final capacitance under pressure of ~95 kPa for NFPU-IL and MESHPIE (100 mV bias voltage at 20 Hz).

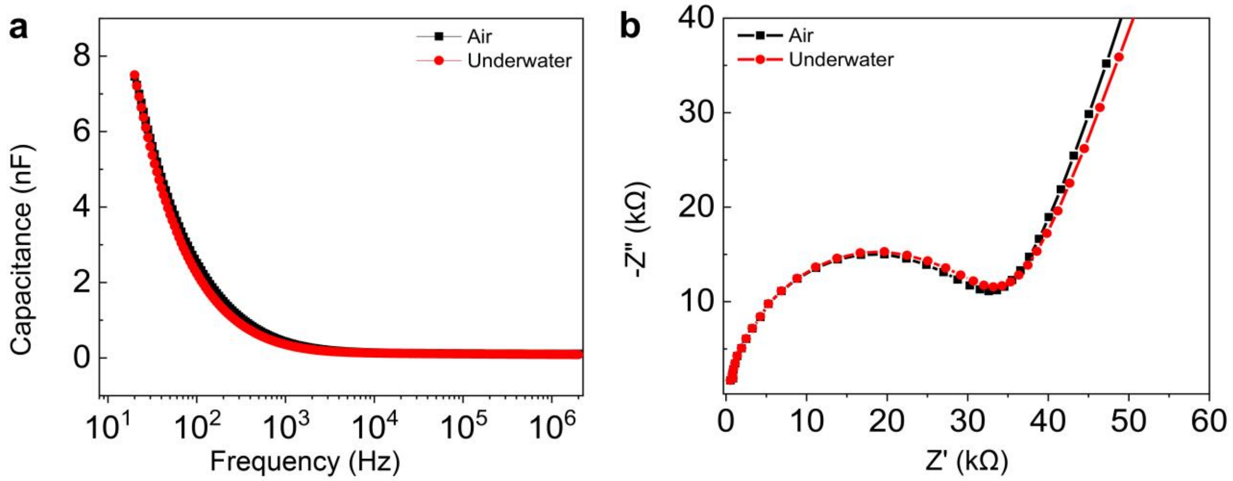


**Supplementary Fig. 22 | | Electrical properties of MESHPIE. a**, Capacitance of MESHPIE in ambient and underwater conditions (applied bias voltage of 1 V at 20 Hz). **b**, Impedance Nyquist plots of MESHPIE in ambient and underwater conditions.

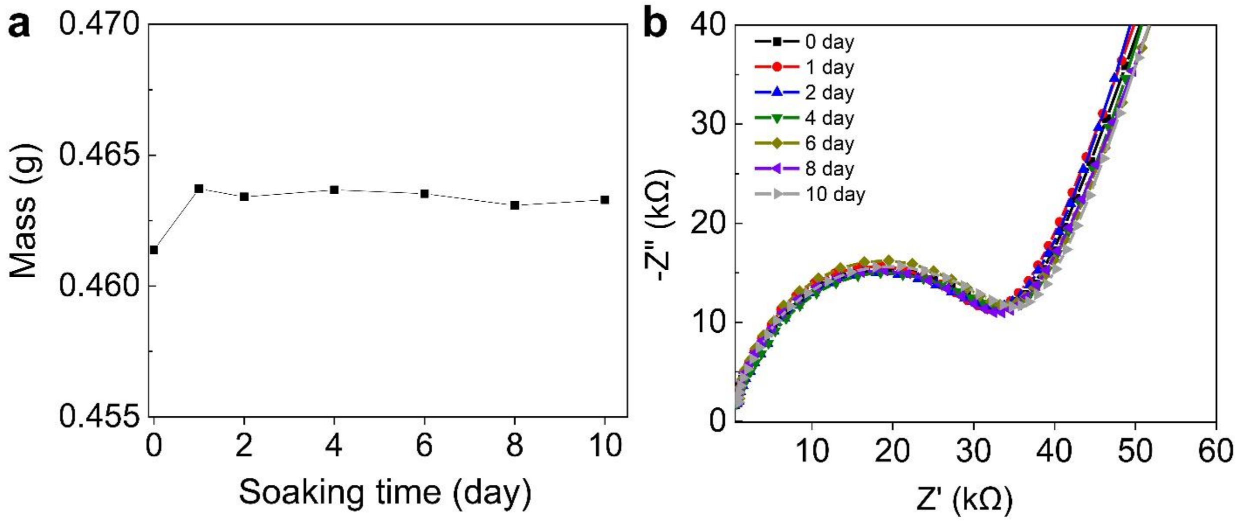


**Supplementary Fig. 23 | a Stability test in underwater conditions.** Changes in the mass of the MESHPIE-based device after being immersed in water for different days. **b** Impedance Nyquist plots of MESHPIE-based device during being immersed in water for ten consecutive days.

# Supplementary Tables

**Supplementary Table 1.** Modification ratio of double bonds in HTPB to PFDT in the synthesis of various MESHEs.

| Sample | -C=C- / -SH (Molar ratio) | Content of fluorine % (atomic concentration.) |
|---|---|---|
| MESHE1 | 1/0.05 | 16.9 |
| MESHE2 | 1/0.10 | 21.8 |
| MESHE3 | 1/0.15 | 28.8 |

**Supplementary Table 2.** Surface atomic concentration calculated from XPS results.

| Sample | Atomic concentration（%） | | | | |
|---|---|---|---|---|---|
| | C | N | O | S | F |
| NFPU | 89.6 | 0.93 | 9.43 | - | - |
| MESHE1 | 70.41 | 3.49 | 8.13 | 1.06 | 16.91 |
| MESHE2 | 68.56 | 1.58 | 6.42 | 1.64 | 21.81 |
| MESHE3 | 62.92 | 1.01 | 5.01 | 2.57 | 28.81 |

**Supplementary Table 3.** A summary of the analysis of the boronic acid content for MESHE1 at various immersion time.

| MESHE1 | Area $cm^2$ ($1340 cm^{-1}$) | Area $cm^2$ ($1360 cm^{-1}$) | Boronic acid content (%) |
|---|---|---|---|
| 0 min | 0 | 1 | 0 |
| 30 min | 0.008 | 0.187 | 4.10 |
| 60 min | 0.010 | 0.230 | 4.17 |
| 90 min | 0.012 | 0.173 | 6.49 |
| 120 min | 0.018 | 0.228 | 7.32 |
| 150 min | 0.020 | 0.250 | 7.41 |

**Supplementary Table 4.** A summary of the analysis of the boronic acid content for MESHE2 at various immersion time.

| MESHE2 | Area $cm^2$ ($1340 cm^{-1}$) | Area $cm^2$ ($1360 cm^{-1}$) | Boronic acid content (%) |
|---|---|---|---|
| 0 min | 0 | 1 | 0 |
| 30 min | 0.005 | 0.292 | 1.68 |
| 60 min | 0.006 | 0.260 | 2.26 |
| 90 min | 0.0068 | 0.258 | 2.57 |
| 120 min | 0.008 | 0.248 | 3.13 |
| 150 min | 0.0082 | 0.253 | 3.14 |

**Supplementary Table 5.** A summary of the analysis of the boronic acid content for MESHE3 at various immersion times.

| MESHE3 | Area $cm^2$ ($1340 cm^{-1}$) | Area $cm^2$ ($1360 cm^{-1}$) | Boronic acid content (%) |
|---|---|---|---|
| 0 min | 0 | 1 | 0 |
| 30 min | 0.0036 | 0.332 | 1.07 |
| 60 min | 0.0040 | 0.322 | 1.23 |
| 90 min | 0.0042 | 0.1473 | 1.32 |
| 120 min | 0.0040 | 0.2627 | 1.50 |
| 150 min | 0.0045 | 0.2809 | 1.58 |

**Supplementary Table 6.** Mechanical properties of various MESHPIEs exhibiting decreased modulus and increased elongation at break as IL concentration increases.

| Sample | Strain (%) | Tensile strength (MPa) | Modulus (MPa) | Toughness ($MJ/m^3$) |
|---|---|---|---|---|
| MESHPIE@10 wt% | 1052±10 | 2.17±0.02 | 0.61±0.01 | 10.82±0.47 |
| MESHPIE@20 wt% | 1105±7 | 1.84±0.08 | 0.50±0.02 | 9.76±0.70 |
| MESHPIE@30 wt% | 1201±13 | 1.66±0.02 | 0.43±0.01 | 9.45±0.71 |
| MESHPIE@40 wt% | 1210±18 | 1.29±009 | 0.40±0.01 | 6.83±0.27 |

**Supplementary Table 7.** Mechanical properties of self-healed MESHPIE at various healing times in air.

| Sample | Strain (%) | Tensile strength (MPa) | Modulus (MPa) | Toughness ($MJ/m^3$) |
|---|---|---|---|---|
| Original | 1201±13 | 1.66±0.02 | 0.43±0.01 | 9.45±0.71 |
| 10 min | 539±5 | 0.61±0.04 | 0.38±0.02 | 0.17±0.37 |
| 20 min | 120±4 | 0.23±0.06 | 0.36±0.02 | 1.99±0.40 |
| 50 min | 1189±15 | 1.53±005 | 0.41±0.01 | 8.93±0.27 |

**Supplementary Table 8.** Mechanical properties of self-healed MESHPIE at various underwater healing times.

| Sample | Strain (%) | Tensile strength (MPa) | Modulus (MPa) | Toughness ($MJ/m^3$) |
|---|---|---|---|---|
| Original | 1201±13 | 1.66±0.02 | 0.43±0.01 | 9.45±0.71 |
| 10 min | 237±5 | 0.29±0.04 | 0.29±0.02 | 0.46±0.21 |
| 30 min | 920±10 | 0.83±0.05 | 0.31±0.01 | 4.08±0.52 |
| 40 min | 1229±12 | 1.33±006 | 0.40±0.01 | 8.47±0.27 |

**Supplementary Table 9.** Literature references comparing self-healing speeds of dielectric elastomers and ionic-based materials with various healing mechanisms.

| No. | Reference | Healing mechanism |
|---|---|---|
| S1 | Khatib, Muhammad, Zohar, Orr, Saliba, Walaa, Srebnik, Simcha and Haick Hossam. "Highly efficient and water-insensitive self-healing elastomer for wet and underwater electronics." Advanced Functional Materials 30.22 (2020): 1910196. | Disulfide bond and hydrogen bond |
| S2 | Zhao, Ye, et al. "Underwater Self-Healing and Recyclable Ionogel Sensor for Physiological Signal Monitoring." ACS Applied Materials & Interfaces (2023). | Ion-dipole interaction, hydrogen bond |
| S3 | Chen, Chao, et al. "A Self-Healing and Ionic Liquid Affiliative Polyurethane toward a Piezo 2 Protein Inspired Ionic Skin." Advanced Functional Materials 32.4 (2022): 2106341. | Electrostatic interaction |
| S4-5 | Xu, Liguo, et al. "A Transparent, Highly Stretchable, Solvent-Resistant, Recyclable Multifunctional Ionogel with Underwater Self-Healing and Adhesion for Reliable Strain Sensors." Advanced Materials 33.51 (2021): 2105306. | Ion-dipole interaction, hydrogen bond |
| S6 | He, Cyuan-Lun, et al. "Super Tough and Spontaneous Water-Assisted Autonomous Self-Healing Elastomer for Underwater Wearable Electronics." Advanced Science 8.21 (2021): 2102275. | Imine bonds and hydrogen bonds |
| S7 | Zhang, Yucheng, et al. "Highly transparent, underwater self-healing, and ionic conductive elastomer based on multivalent ion–dipole interactions." Chemistry of Materials 32.15 (2020): 6310-6317. | Ion-dipole interaction |
| S8 | Li, Mengqing, et al. "A hydrophobic eutectogel with excellent underwater Self-adhesion, Self-healing, Transparency, Stretchability, ionic Conductivity, and fully recyclability." Chemical Engineering Journal 472 (2023): 145177. | Hydrogen bonding, π-π, cation-π, and ion–dipole interactions |
| S9 | He, Cyuan-Lun, et al. "Super Tough and Spontaneous Water-Assisted Autonomous Self-Healing Elastomer for Underwater Wearable Electronics." Advanced Science 8.21 (2021): 2102275. | Imine bonds and hydrogen bonds |
| S10 | Boahen, Elvis K., et al. "Ultrafast, autonomous self-healable iontronic skin exhibiting piezo-ionic dynamics." Nature Communications 13.1 (2022): 7699 | Disulfide bond |
| S11 | Xu, Liguo, et al. "A Transparent, Highly Stretchable, Solvent-Resistant, Recyclable Multifunctional Ionogel with Underwater Self-Healing and Adhesion for Reliable Strain Sensors." Advanced Materials 33.51 (2021): 2105306. | Ion-dipole interaction, hydrogen bond |

| | | |
|---|---|---|
| S12 | Kim, Seon-Mi, et al. "Superior toughness and fast self-healing at room temperature engineered by transparent elastomers." Advanced Materials 30.1 (2018): 1705145. | Disulfide bond |
| S13 | Ying, Wu Bin, et al. "Waterproof, highly tough, and fast self-healing polyurethane for durable electronic skin." ACS applied materials & interfaces 12.9 (2020): 11072-11083. | Disulfide bond |
| S14 | Cao, Yue, et al. "Self-healing electronic skins for aquatic environments." Nature Electronics 2.2 (2019): 75-82. | Ion-dipole interaction |
| S15 | Chen, Shaojun, et al. "Development of zwitterionic polyurethanes with multi-shape memory effects and self-healing properties." Journal of Materials Chemistry A 3.6 (2015): 2924-2933. | Water or moisture induced hydration |
| S16 | Ying, Wu Bin, et al. "Waterproof, highly tough, and fast self-healing polyurethane for durable electronic skin." ACS applied materials & interfaces 12.9 (2020): 11072-11083. | Disulfide bond |
| S17 | Truong, Thuy Thu, et al. "Tailoring the hard–soft interface with dynamic diels–alder linkages in polyurethanes: toward superior mechanical properties and healability at mild temperature." Chemistry of Materials 31.7 (2019): 2347-2357. | Dynamic Diels–Alder bond |
| S18 | Li, Yu-han, et al. "Tuning hard phase towards synergistic improvement of toughness and self-healing ability of poly (urethane urea) by dual chain extenders and coordinative bonds." Chemical Engineering Journal 393 (2020): 124583. | Disulfide bond |
| S19 | Mei, Jin-Feng, et al. "A highly stretchable and autonomous self-healing polymer based on combination of pt··· pt and π–π interactions." Macromolecular rapid communications 37.20 (2016): 1667-1675. | π–π Interactions |
| S20 | Kim, Seon-Mi, et al. "Superior toughness and fast self-healing at room temperature engineered by transparent elastomers." Advanced Materials 30.1 (2018): 1705145. | Disulfide bond |

## Supplementary References


1 Ying, W. B. *et al.* A Biologically Muscle-Inspired Polyurethane with Super-Tough, Thermal Reparable and Self-Healing Capabilities for Stretchable Electronics. *Adv. Funct. Mater.* **31**, 2009869 (2021).

2 Chen, C. *et al.* A Self-Healing and Ionic Liquid Affiliative Polyurethane toward a Piezo 2 Protein Inspired Ionic Skin. *Adv. Funct. Mater.* **32**, 2106341 (2021).

3 Yang, Y. & Urban, M. W. Self-healing polymeric materials. *Chem Soc Rev* **42**, 7446-7467 (2013).

4 Cao, Y. *et al.* Self-healing electronic skins for aquatic environments. *Nat Electron* **2**, 75-82 (2019).

5 Jin, M. L. *et al.* An Ultrasensitive, Visco-Poroelastic Artificial Mechanotransducer Skin Inspired by Piezo2 Protein in Mammalian Merkel Cells. *Adv. Mater.* **29**, 1605973 (2017).

6 Boahen, E. K. *et al.* Ultrafast, autonomous self-healable iontronic skin exhibiting piezo-ionic dynamics. *Nat Commun* **13**, 7699 (2022).

7 Zhang, Y. *et al.* Highly Transparent, Underwater Self-Healing, and Ionic Conductive Elastomer Based on Multivalent Ion–Dipole Interactions. *Chem. Mater.* **32**, 6310-6317 (2020).

8 Cao, Y. *et al.* A Transparent, Self-Healing, Highly Stretchable Ionic Conductor. *Adv. Mater.* **29**, 1605099 (2017).

9 Lassegues, J. C., Grondin, J., Cavagnat, D. & Johansson, P. New interpretation of the CH stretching vibrations in imidazolium-based ionic liquids. *J Phys Chem A* **113**, 6419-6421 (2009).

10 Wu, J. Understanding the Electric Double-Layer Structure, Capacitance, and Charging Dynamics. *Chem Rev* **122**, 10821-10859 (2022).

11 Singh, M. B. & Kant, R. Debye–Falkenhagen dynamics of electric double layer in presence of electrode heterogeneities. *Journal of Electroanalytical Chemistry* **704**, 197-207 (2013).

12 Joshi, J. H., Kanchan, D. K., Joshi, M. J., Jethva, H. O. & Parikh, K. D. Dielectric relaxation, complex impedance and modulus spectroscopic studies of mix phase rod like cobalt sulfide nanoparticles. *Materials Research Bulletin* **93**, 63-73 (2017).

13 You, I. *et al.* Artificial multimodal receptors based on ion relaxation dynamics. *Science* **370**, 961-965 (2020).

14 Liang, J. *et al.* Structure and dynamics of ions in dipolar solvents: a coarse-grained simulation study. *Soft Matter* **17**, 6305-6314 (2021).

15 Hughes, R. W. & Warner, M. LEDs driven by AC without transformers or rectifiers. *Sci Rep* **11**, 963 (2021).

16 Luo, H. *et al.* Shape Memory-Enhanced Electrical Self-Healing of Stretchable Electrodes. *Appl. Sci.* **8**, 393 (2018).

17 Gong, C. *et al.* A healable, semitransparent silver nanowire-polymer composite conductor. *Adv. Mater.* **25**, 4186-4191 (2013).
