# Supplementary material for: Ultrafast underwater self-healing piezo-ionic elastomer via dynamic hydrophobic-hydrolytic domains
Source: Nat Commun. 2024 Mar 8;15:2129. doi: 10.1038/s41467-024-46334-4 (PMC10923942; doi:10.1038/s41467-024-46334-4)
Supplement: Supplementary file 3 — Description of additional supplementary files [file 41467_2024_46334_MOESM3_ESM.pdf]

Description of additional supplementary files

Supplementary Movie 1 | Cut and spliced demonstration of MESHPIE. This movie presents the underwater self-healing demonstration of MESHPIE.

Supplementary Movie 2 | Pressure response before and after self-healing in air. This movie presents the demonstration of pressure-induced tactile response of MESHPIE-based device before and after self-healing in air.

Supplementary Movie 3 | Underwater sensing performance of MESHPIE. This movie presents the underwater sensing performance of MESHPIE when integrated into a toy submarine.

Supplementary Movie 4 | Visualization of LED intensity upon impact with underwater object. This movie presents the demonstration of a MESHPIE-based device to visualize changes in LED intensity upon impact with underwater object.

Supplementary Movie 5 | Visualization of LED intensity after underwater self-healing. This movie presents the underwater self-healing performance of MESHPIE-based device with an LED.

Supplementary Movie 6 | Demonstration of electrical self-healing in underwater condition. This movie presents the demonstration of electrical self-healing of MESHPIE-based device in underwater condition.
