# Supplementary material for: Ultrafast underwater self-healing piezo-ionic elastomer via dynamic hydrophobic-hydrolytic domains
Source: Nat Commun. 2024 Mar 8;15:2129. doi: 10.1038/s41467-024-46334-4 (PMC10923942; doi:10.1038/s41467-024-46334-4)
Supplement: Supplementary file 2 — Peer review file [file 41467_2024_46334_MOESM2_ESM.pdf]

nature portfolio

Peer Review File

Ultrafast underwater self-healing piezo-ionic elastomer via dynamic hydrophobic-hydrolytic domains

Open Access This file is licensed under a Creative Commons Attribution 4.0 International License, which permits use, sharing, adaptation, distribution and reproduction in any medium or format, as long as you give appropriate credit to the original author(s) and the source, provide a link to the Creative Commons license, and indicate if changes were made. In the cases where the authors are anonymous, such as is the case for the reports of anonymous peer reviewers, author attribution should be to 'Anonymous Referee' followed by a clear attribution to the source work. The images or other third party material in this file are included in the article's Creative Commons license, unless indicated otherwise in a credit line to the material. If material is not included in the article's Creative Commons license and your intended use is not permitted by statutory regulation or exceeds the permitted use, you will need to obtain permission directly from the copyright holder. To view a copy of this license, visit http://creativecommons.org/licenses/by/4.0/.

REVIEWER COMMENTS

Reviewer #1 (Remarks to the Author):

This paper reported an ultrafast underwater molecularly engineered self-healing piezo-ionic elastomer with self-healing properties and mechanosensitive ion channels. The elastomer is composed of the hydrophobic C-F groups and hydrolytic boronate ester bonds. Due to the boronate ester bonds involving different mechanisms in the air and underwater, the material shows outstanding self-healing efficiencies. In addition, the elastomer performs exceptionally well in pressure sensitivity. The mechanically sensitive elastomeric sensor has found effective applications in LED modulation and underwater submarine data transmission. It offers an innovative approach to the fields of human-machine interactions and underwater robots. However, the paper needs to be improved even if multiple tests are offered here to demonstrate the effectiveness of this content.

1. In Fig. 2c and 2d, how did the author get the self-healing speed and efficiencies of the MESHPIEs? Should the author consider adding the error bar to each set of data in the figure to ensure scientific and rigorous?

2. In Fig. 4i, does the test involve the same sample changing different conditions over a sustained period of time?

3. In Fig. 5e, the figure shows the changes in LED intensity under touch-sensitive pressure before and after self-healing. This part is placed after the application, whether this order is logical. The author should think again.

4. At the end of the paper, the underwater application test of the sensor is given. It is recommended to supplement the electrical test of the above materials with underwater electrical tests.

5. As shown in Fig.1d and 2a, the hydrophobic structure is located in the outer layer of the gel. Please provide specific examples and proof.

Reviewer #2 (Remarks to the Author):

This work introduced an ultrafast underwater self-healing piezo-ionic elastomer via the synergy of hydrophobicity and B-O hydrolytic reaction. The optimized piezo-ionic elastomer showed impressive self-healing efficiencies and sensing performance. The manuscript was also well organized with dense data for supporting their conclusions. I overall recommend the publication of this work in Nature Communications. However, before acceptance, a major revision is necessary for further polishing their findings.

Comments:

1. The authors adjusted the content of C-F groups to achieve the best synergy for self-healing. However, the changing trends in air and underwater are quite different in Figure 2c. This difference should be more clearly explained.

2. Introducing dynamic bonds like B-O for room-temperature healing generally sacrifice the elastic properties, which are important for durable sensing applications. The reversible compressive experiments on different samples should be provided to study its effect on elasticity.

3. How about the self-healing efficiency at the totally dried condition? This can exclude the effect of water, which help highlight the role of hydrolytic reaction underwater.

4. The vertical labels for Figure 2c and d should be unified for better comparison.

5. The fitted IR peak areas do not directly represent the actual contents, due to the difference of absorption coefficient at different wavenumbers. Corresponding revision should be made in Figure 2b (the authors may refer to: Adv. Mater. 2023, 35, 2209581). Also in Figure 3, Intensity should be "Absorbance" for IR spectra.

6. The formats for all the references should be carefully checked to comply with the journal's requirements.

Reviewer #3 (Remarks to the Author):

The authors synthesized an ultrafast underwater molecularly engineered self-healing piezo-ionic elastomer (MESHPIE) inspired by the cephalopod's suckers, which possessed self-healing properties and mechanosensitive ion channels. They integrated a material-based device into an underwater submarine for signal transmission and LED illumination to demonstrate its potential for underwater robotics. However, the MESHPIE-based device characterizations and demonstrations are insufficient and require further improvements and analysis. Major revision is needed before further consideration. Below are some comments:

1. It is mentioned in the introduction that "However, these materials exhibited relatively low self-healing efficiencies and speeds owing to the limited synergistic effect between the hydrophobic groups and dynamic bonds". Corresponding data need to be provided for the relatively low self-healing efficiencies and speeds here.

2. The labels in Figure 1 need to be improved. For example, the cations in Figure 1c are Ca2+ ions, which need to be labeled in the figure.

3. In Figure 2f, quantitative characterizations should be provided to demonstrate the absence of water leakage when the material is loaded.

4. Figure 2i compares the self-healing speeds of different self-healing materials at different temperatures. The author tested the self-healing speed at room temperature. How about the effect of temperature on its self-healing speed? And how about the self-healing speed at higher temperature?

5. In Figure 4b, the authors state that the ion trap and release before and after inducing pressure generate a low initial capacitance and a high final capacitance, respectively, owing to the efficient pumping of ions. Is there evidence that the material undergoes ion release under pressure stimulation?

6. In Figure 4h, the response time of the device is 281 ms, which is slower than other ionic conductors-based devices. Why is the response time so slow? Is it possible to improve the response time?

7. Although the MESHPIE can be placed in water, other components of the MESHPIE-based devices might fail? Please comment on this.

8. If the devices based on this material are to be used in water or air, their stability is very important. How about the stability of the material in water? How about the shelf-life?

9. The demonstration in Figure 5e does not effectively reflect the advantages of the MESHPIE-based device. Most ionic conductors-based pressure sensor can do.

<u>**Reply to Reviewer #1**</u>

This paper reported an ultrafast underwater molecularly engineered self-healing piezo-ionic elastomer with self-healing properties and mechanosensitive ion channels. The elastomer is composed of the hydrophobic C-F groups and hydrolytic boronate ester bonds. Due to the boronate ester bonds involving different mechanisms in the air and underwater, the material shows outstanding self-healing efficiencies. In addition, the elastomer performs exceptionally well in pressure sensitivity. The mechanically sensitive elastomeric sensor has found effective applications in LED modulation and underwater submarine data transmission. It offers an innovative approach to the fields of human-machine interactions and underwater robots. However, the paper needs to be improved even if multiple tests are offered here to demonstrate the effectiveness of this content.

<u>**Response:**</u> We appreciate the positive and constructive comments from the reviewer. We have revised the manuscript according to the reviewer's comments.

Q1) *In Fig. 2c and 2d, how did the author get the self-healing speed and efficiencies of the MESHPIEs? Should the author consider adding the error bar to each set of data in the figure to ensure scientific and rigorous?*

<u>**Response:**</u> We appreciate the reviewer for the valuable comments. The self-healing speed (μm/min) can be defined as the speed taken by a damaged material to attain maximum healing efficiency with or without external stimuli. Generally, the self-healing speed is calculated based on the ratio of [notch size] (μm) / self-healing time (min). In this work, since the sample was cut completely, the notch size was taken to be the thickness of the sample (*Adv. Funct. Mater.* **31**, 2009869 (2021)*; Adv. Funct. Mater.* **32**, 2106341 (2022)). The self-healing speed in **Fig. 2c** and **2d** were therefore calculated based on the definition of the self-healing speed. The formula is given as below:

$$\text{Self}-\text{healing speed} = \frac{\text{Thickness（μm）}}{\text{Self}-\text{healing time（min）}}$$

The self-healing efficiency can be defined as the ability of a material to recover a particular property after damage relative to the original or the undamaged material (*Chem. Soc. Rev.* ***42****, 7446–7467 (2013)*). This quantifies the evaluation of a healed material to determine the extent of recovery of initial properties after mechanical damage. The self-healing efficiency in this work was calculated from **Fig. 2g and supplementary Fig. 13,** based on the ratio of (the toughness of healed sample after) / (the toughness of original sample) x 100%. The formula is as shown below:

$$\text{Self}-\text{healing efficiency} = \frac{\text{Toughness}_{healed}}{\text{Toughness}_{\text{Original}}} \times 100\%$$

To address the reviewer's concern with regard to error bars, we have replaced **Figs. 2c** and **2d** with **Figs. R1a** and **R1b** respectively in the revised manuscript. To clarify the basis of the self-healing evaluation methods, we have added the following sentences and the equations for both self-healing speed and self-healing efficiency to the revised Supplementary Information on page 3 and the following references to the Supplementary References.

"*The self-healing speed (μm/min) can be defined as the rate at which the damaged sample achieves maximum self-healing efficiency with or without external stimuli. The self-healing speed is often determined from the ratio of [notch size] (μm) / self-healing time (min)*[1,2]*. However, in this study, the sample was cut entirely with the knife penetrating through to the lower part, hence, the notch size was determined by the thickness of the sample. Furthermore, the self-healing efficiency was calculated from the ratio of (the toughness of healed sample / the toughness of original sample)*[3] *x 100%. The formulae are as follows:"*

$$\text{Self}-\text{healing speed} = \frac{\text{Thickness (μm)}}{\text{Self−healing time (min)}} \qquad (1)$$

$$\text{Self}-\text{healing efficiency} = \frac{\text{Toughness}_{healed}}{\text{Toughness}_{\text{Original}}} \times 100\% \qquad (2)$$

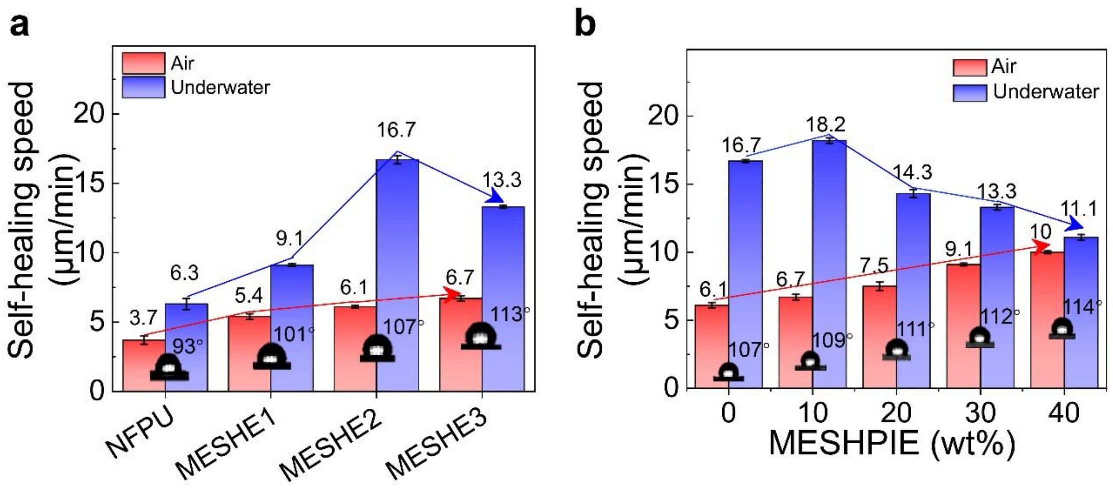

Figure. R1 | **a** Self-healing speeds of the various MESHEs in both ambient and underwater conditions. **b** Self-healing speeds of the various MESHPIEs with different IL concentrations in both ambient and underwater conditions.

[1] Ying, W. Bin *et al.* A Biologically Muscle-Inspired Polyurethane with Super-Tough, Thermal Reparable and Self-Healing Capabilities for Stretchable Electronics. *Adv. Funct. Mater.* **31**, 2009869 (2021)

[2] Chen, C. *et al.* A Self-Healing and Ionic Liquid Affiliative Polyurethane toward a Piezo 2 Protein

Inspired Ionic Skin. *Adv. Funct. Mater.* **32**, 2106341 (2022).
[3] Yang, Y. & Urban, M. W. Self-healing polymeric materials. *Chem. Soc. Rev.* **42**, 7446–7467 (2013).

Q2) *In Fig. 4i, does the test involve the same sample changing different conditions over a sustained period of time?*

Response: We appreciate the reviewer for this comment. The experiment depicted in Fig. 4i is a continuous investigation conducted on the same sample. The specific steps are as follows: (1) cyclic capacitance test was conducted on the original sample, (2) cut and self-healed experiments were performed on the sample in air at ambient conditions, (3) cyclic capacitance test was conducted on the self-healed sample, (4) cut and self-healed experiments were performed on the sample underwater, (5) lastly, cyclic capacitance test was conducted after underwater self-healing. The obtained results support the durability of MESHPIE-based sensor device after self-healing in air and underwater conditions.

Q3) *In Fig. 5e, the figure shows the changes in LED intensity under touch-sensitive pressure before and after self-healing. This part is placed after the application, whether this order is logical. The author should think again.*

Response: We thank the reviewer for pointing this out. We agree that the logical ordering of figures is important for easy comprehension of our work. As suggested by the reviewer, we have reorganized the figures, with Fig. 5e placed before the application in the revised manuscript as shown in **Fig. R2**.

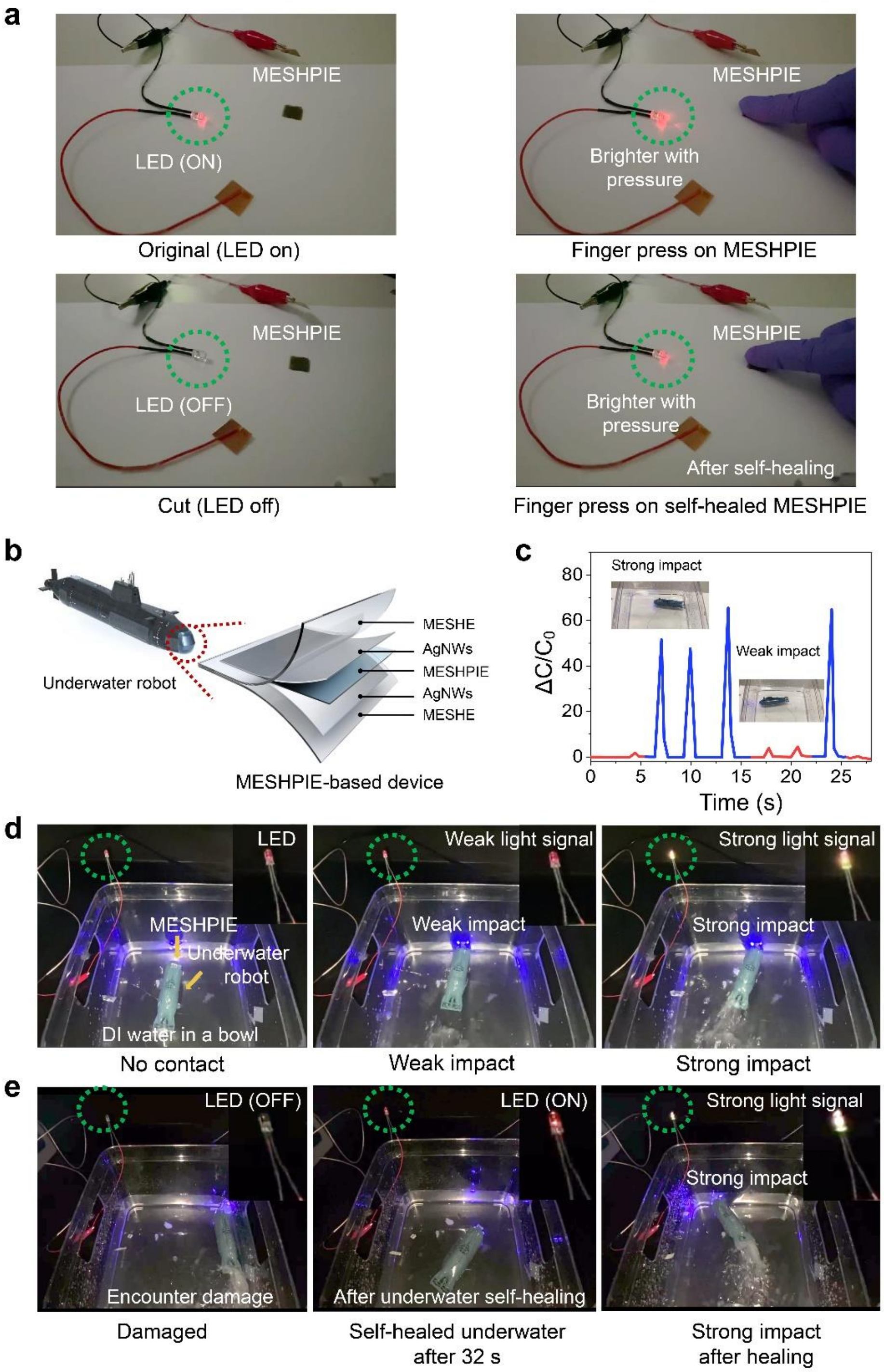

Fig. R2 | Demonstration of practical device performance in ambient and aquatic environments. a Changes in LED intensities under touch-induced pressures, before and after self-healing situations. **b** Illustration of MESHPIE-based device architecture, which is incorporated into an underwater toy submarine for practical demonstration. **c** Sensing responses upon impact with underwater object (wall of the container). **d** Photographs showing the visualization of changes in LED intensity upon impact with underwater object. **e** Pressure response of self-healed device after encountering severe damage underwater.

Q4) *At the end of the paper, the underwater application test of the sensor is given. It is recommended to supplement the electrical test of the above materials with underwater electrical tests.*

Response: We appreciate the reviewer for this valuable comment. In our study the impedance and capacitance are great experiments that characterize the electrical performance of our material. In order to support the electrical performance of our material in underwater conditions, we have conducted impedance (Nyquist plots) and capacitance tests (**Fig. R3a** and **R3b,** respectively) of MESHPIE in both ambient and underwater conditions. The results showcase the exceptional underwater electrical performance and stability of MESHPIE, ensuring the stable underwater application of MESHPIE-based devices. Additionally, **Supplementary Movie 6** (see detail explanation in Supplementary Note 6) not only supports the electrical self-healing of the MESHPIE-based device, but also affirms the electrical performance of the device in underwater conditions.

To supplement the underwater electrical tests, we have added **Fig. R3a** and **R3b** as **Supplementary Fig. 22**, and the following sentences on page 8 of the revised Supplementary Information.

"to support the underwater electrical performance of MESHPIE, capacitance (Supplementary Fig. 22a) and impedance (Supplementary Fig. 22b) tests were conducted in both ambient and underwater conditions. The results showcase the exceptional underwater electrical performance and stability of MESHPIE, ensuring the stable underwater application of MESHPIE-based devices."

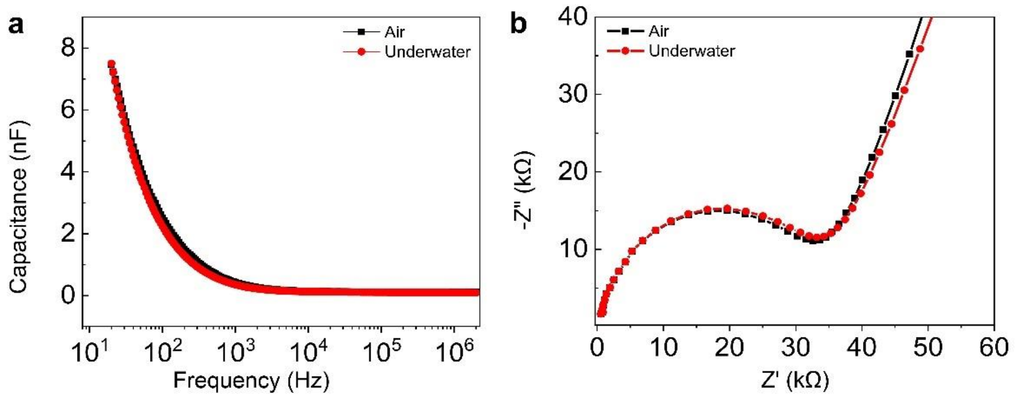

Fig. R3 | a Capacitance of MESHPIE in ambient and underwater conditions (applied bias voltage of 1 V at 20 Hz, no applied pressure). **b** Impedance Nyquist plots of MESHPIE in ambient and underwater conditions.

Q5) *As shown in Fig.1d and 2a, the hydrophobic structure is located in the outer layer of the gel. Please provide specific examples and proof.*

Response: We thank the reviewer for the valuable comments. Actually, the hydrophobic structure permeates the entire polyurethane material owing to the introduction of C–F side chain group in soft segment, as depicted in **Fig. 1g**, original manuscript. Thus, the entire MESHPIE exhibits hydrophobic

properties, as evidenced by the water contact angles depicted in Figs. 2c-d, original manuscript, and **Supplementary Fig. 6**. Please refer to this reference (*Mater. Horiz.*, **8**, 2761-2770 (2021)) for example of this phenomenon.

Nevertheless, in this study, our discussion focuses on the interaction between MESHPIE and water molecules action on its surface. **Fig. 1d** and **Fig. 2a** highlight the hydrophobic effect acting as a barrier to repel the majority of water molecule ingress. As discussed in the original manuscript, increasing the C–F group density in MESHPIE increases the contact angle, indicating excellent hydrophobicity. As proof of hydrophobicity, **Supplementary Fig. 6** is included here as **Fig. R4** for easy reference to the reviewer.

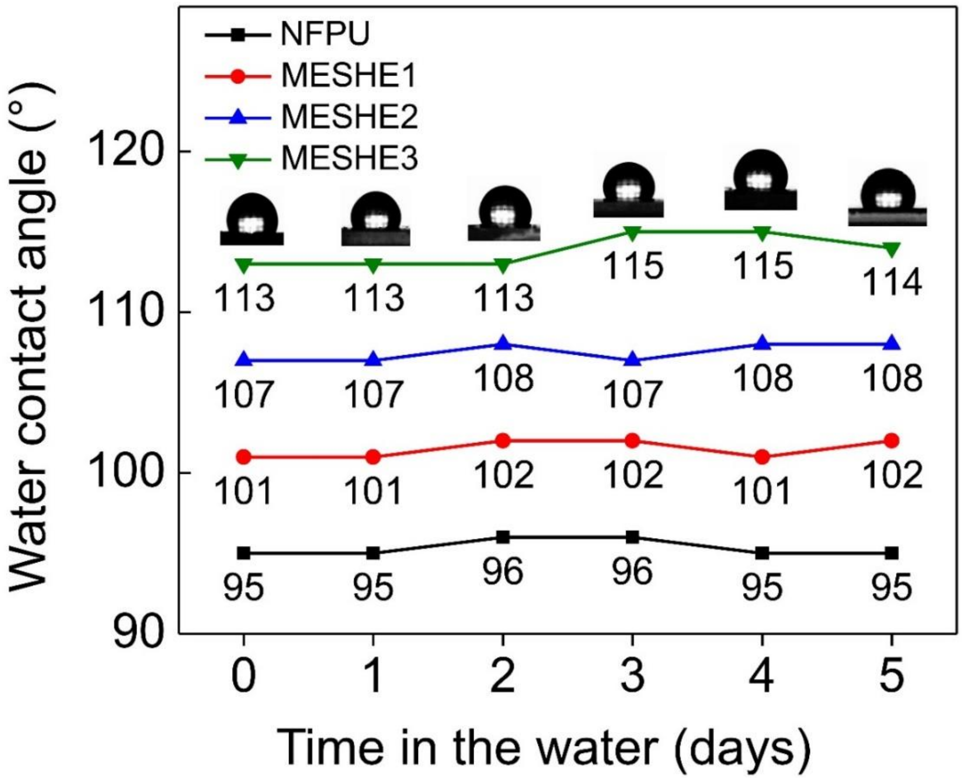

Fig. R4 | Changes of water contact angles. Images of the water contact angles of the various PUs and calculated angles representing the changes in their hydrophobicity after being immersed in water for five consecutive days.

<u>**Reply to Reviewer #2**</u>

This work introduced an ultrafast underwater self-healing piezo-ionic elastomer via the synergy of hydrophobicity and B-O hydrolytic reaction. The optimized piezo-ionic elastomer showed impressive self-healing efficiencies and sensing performance. The manuscript was also well organized with dense data for supporting their conclusions. I overall recommend the publication of this work in Nature Communications. However, before acceptance, a major revision is necessary for further polishing their findings.

<u>**Response:**</u> We appreciate the reviewer for the constructive comments and recommendation of our manuscript. We have revised the manuscript according to the reviewer's comments.

Q1) *The authors adjusted the content of C-F groups to achieve the best synergy for self-healing. However, the changing trends in air and underwater are quite different in Figure 2c. This difference should be more clearly explained.*

<u>**Response:**</u> We thank the reviewer for asking us to clarify the explanation on **Fig. 2c**. As discussed in the original manuscript, the introduction of higher C–F groups not only leads to the formation of dense hydrophobicity, but also enhances dipole-dipole interactions. The changing trend of self-healing in air is attributed to enhanced dipole-dipole interactions with the introduction of higher C–F groups in the MESHEs as depicted in **Fig. 2c**. On the other hand, the underwater self-healing speed of the MESHEs depends largely on the reversible hydrolysis/re-esterification reactions of boronate ester bond caused by the ingress of small quantity of water molecules. Thus, the changing trend of underwater self-healing speed increases with higher C–F group content, however, MESHE3, with the highest C–F group content exhibited a drastic decrease in underwater self-healing speed, owing to denser hydrophobicity which limited the amount of water ingress necessary for effective self-healing through boronate ester bond hydrolysis.

Q2) *Introducing dynamic bonds like B-O for room-temperature healing generally sacrifice the elastic properties, which are important for durable sensing applications. The reversible compressive experiments on different samples should be provided to study its effect on elasticity.*

<u>**Response:**</u> We appreciate the reviewer for this important comment. In our system, the chain extender GBDB containing B-O bonds, in conjunction with IPDI, forms the hard segment of polyurethane, providing physical cross-linking points in the polyurethane network to ensure excellent mechanical performance. In **Fig. R5**, BPU is a polyurethane composed of monomers IPDI, HTPB, and GBDB. The monomer ratios of BPUs are presented in **Table R1**, showing that BPUs have the same soft segment content and a gradually increasing chain extender weight, indicating an augmented dynamic bond content.

Therefore, in our system, the mechanical modulus of polyurethane increases with the dynamic B-O bond content, rather than being compromised, as depicted in **Fig. R5** and **Table R2**.

Furthermore, to address the reviewer's concern, we conducted compression experiments on MESHPIE to investigate its elasticity, as shown in **Fig. R6**. The MESHPIE exhibited excellent elastic recovery without obvious plastic deformation. The nearly similar compressive stress-strain loading/unloading curves emphasize the stability and outstanding fatigue resistance of MESHPIE, an indicative of its remarkable elastic recovery capabilities (**Fig. R6a**). Additionally, we subjected the material to 100 consecutive compression cycles to further evaluate its fatigue resistance (**Fig. R6b**). Following 100 cycles of compression tests, the stress attenuation registered a mere 1.93%, attesting to the material's outstanding fatigue resistance.

To support the elastic behavior of our material, we have included the following sentences on page 4 and **Fig. R6** as **Supplementary Fig. 13** in the revised Supplementary Information.

"To examine the reversible compressibility of MESHPIE for effective device performance, compressive stress-strain tests were performed with cyclic loading/unloading strain (Supplementary Fig. 13). The compressive test exhibited similar loading/unloading curves emphasizing the stability and outstanding fatigue resistance of MESHPIE, indicative of its remarkable elastic recovery capabilities (Supplementary Fig. 13a). Additionally, we subjected the material to 100 consecutive compression cycles to further evaluate its fatigue resistance (Supplementary Fig. 13b). Following 100 cycles of compression tests, the stress attenuation registered a mere 1.93%, attesting to the material's outstanding fatigue resistance."

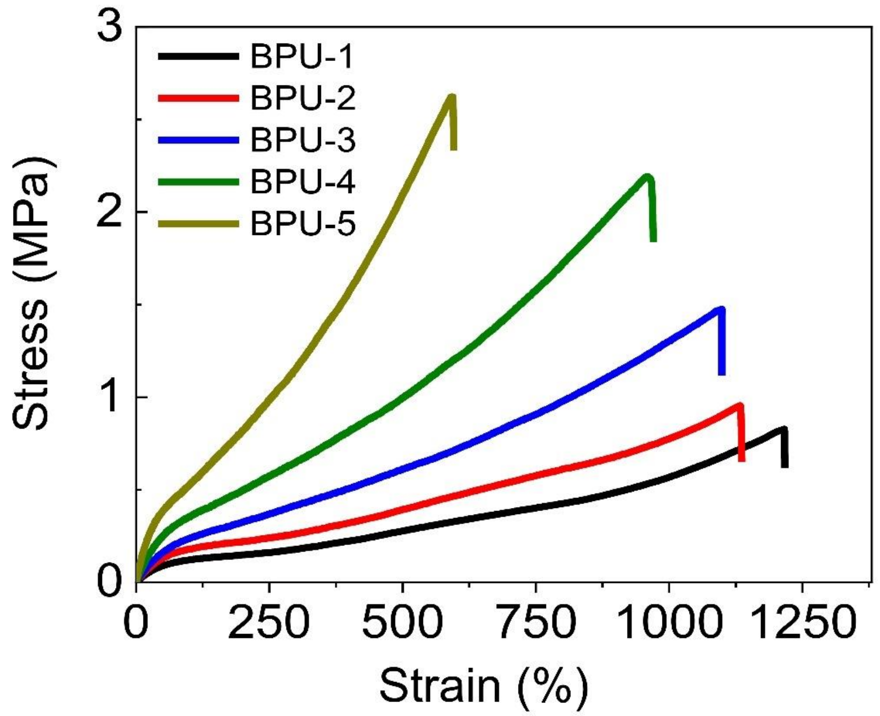

Fig. R5 | a Stress-strain curves of BPUs containing different boronate ester bond contents.

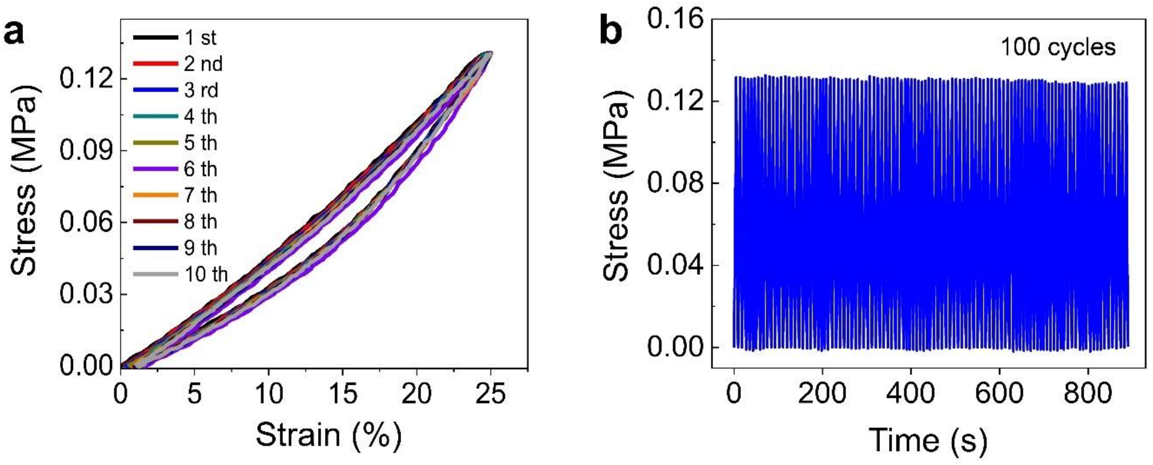

Fig. R6 | a Sequential cyclic compressive stress-strain curves of MESHPIE at 25% strain. **b** Fatigue resistance test of MESHPIE by sequential cyclic compression test (100 times).

Table R1. The componential mole ratios of various BPUs.

Sample	Components GB (g)	Components HTPB (g)	Components IPDI (ml)	Catalyst DBTDL (g)	Hard Segments (%)	Mw (g/mol)	PDI	Content of GBDB (wt%)
BPU-1	1.0	10	1.7	0.25	21.3	3.5	2.3	7.9
BPU-2	1.5		2.2		27.0	3.9	2.4	10.9
BPU-3	2.0		2.6		31.5	3.7	2.2	13.7
BPU-4	2.5		3.1		35.9	3.5	2.1	16.0
BPU-5	3.0		3.5		39.4	3.6	2.3	18.2

Table R2. The Mechanical properties of various BPUs.

Sample	Tensile strength (MPa)	Elongation at break (%)	Modulus (MPa)	Toughness (MJ/m^3)
BPU-1	0.83±0.04	1213±9	0.26±0.01	4.36±0.34
BPU-2	0.95±0.03	1132±6	0.30±0.01	5.25±0.65
BPU-3	1.47±0.03	1098±9	0.42±0.01	7.81±0.52
BPU-4	2.20±0.01	961±11	1.30±0.02	10.21±0.19
BPU-5	2.61±005	594±5	0.87±0.01	7.45±0.23

Q3) *How about the self-healing efficiency at the totally dried condition? This can exclude the effect of water, which help highlight the role of hydrolytic reaction underwater.*

Response: We thank the reviewer for the valuable comment. We totally agree that to highlight the role of hydrolytic reaction underwater, it is important to exclude the effect of water in a control experiment. The hydrolysis/re-esterification reactions of borate ester bonds occur under high humidity conditions (relative humidity, RH 85%) or with minor addition of water molecules (*J. Mater. Chem. A*, **9**, 14630-14655 (2021); *Macromolecules* **48**, 7, 2098–2106 (2015)). As discussed in this study, the self-healing efficiency in air (94.5% after 50 min) was also conducted at ambient conditions (RH 20–40%), as depicted in **Supplementary Fig. 14**, revised Supplementary Information. The RH 20–40% is not enough to initiate hydrolysis of boronate ester bonds, therefore, the self-healing test in air is sufficient as a control experiment to study the importance of hydrolysis on self-healing.

Q4) *The vertical labels for Figure 2c and d should be unified for better comparison.*

Response: We appreciate the reviewer for this sincere comment. As recommended by the reviewer, we have unified the vertical labels for **Figs. 2c** and **2d** for better comparison.

We have replaced **Figs. 2c** and **2d** with **Fig. R7a** and **R7b**, respectively, in the revised Manuscript.

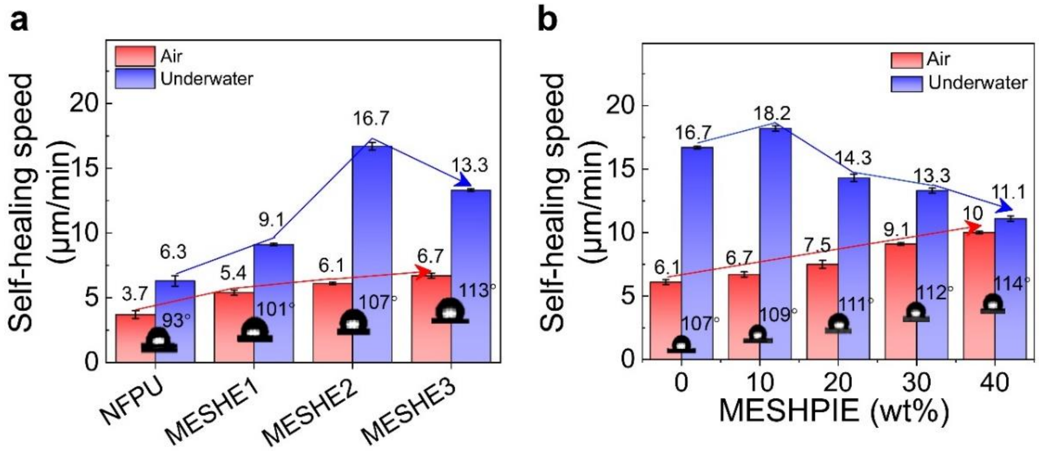

Fig. R7 | a Self-healing speeds of the various MESHEs in both ambient and underwater conditions. **b** Self-healing speeds of the various MESHPIEs with different IL concentrations in both ambient and underwater conditions.

Q5) *The fitted IR peak areas do not directly represent the actual contents, due to the difference of absorption coefficient at different wavenumbers. Corresponding revision should be made in Figure 2b (the authors may refer to: Adv. Mater. 2023, 35, 2209581). Also in Figure 3, Intensity should be "Absorbance" for IR spectra.*

Response: We thank the reviewer for the valuable comments. We totally agree with the proposed modification to the description of **Fig. 2b** and the specification for IR spectra in **Fig. 3**. The characteristic

peak at 1360 cm^{-1} represents the boron oxygen bond (B–O) of boronate esters before immersing in water. After immersion in deionized water for 30 minutes or more, a new characteristic peak attributed to boronic acid groups (-B(OH)$_2$) appeared at 1341 cm^{-1}, indicating hydrolysis of the boronate ester bonds. Therefore, the fractional area of the boronic acid group peaks, obtained through deconvolution calculations of the characteristic peaks at different immersion times, reflects the degree of hydrolysis at different immersion times.

To address the reviewer's concern, we have replaced **Fig. 2b**, **Fig. 3a, b, d, e**, **Supplementary Fig. 7**, and **Supplementary Fig. 8** with **Fig. R8**, **Fig. R9 a, b, c, d**, **Fig. R10**, and **Fig. R11**, respectively, in the revised Manuscript and revised Supplementary Information.

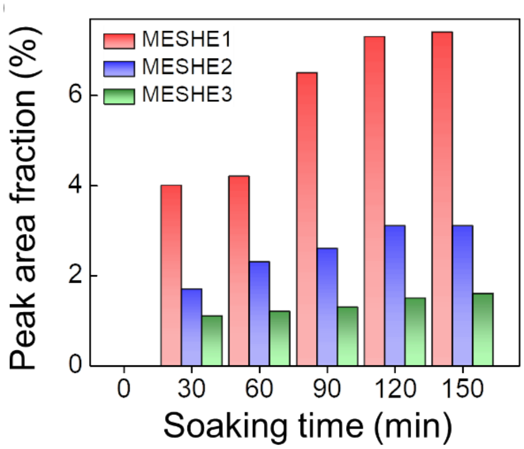

Fig. R8 | Analysis of the hydrolytic behavior of the various MESHEs at different immersion times. This graph represents the calculated peak area fraction of boronic acid group as a function of the soaking times.

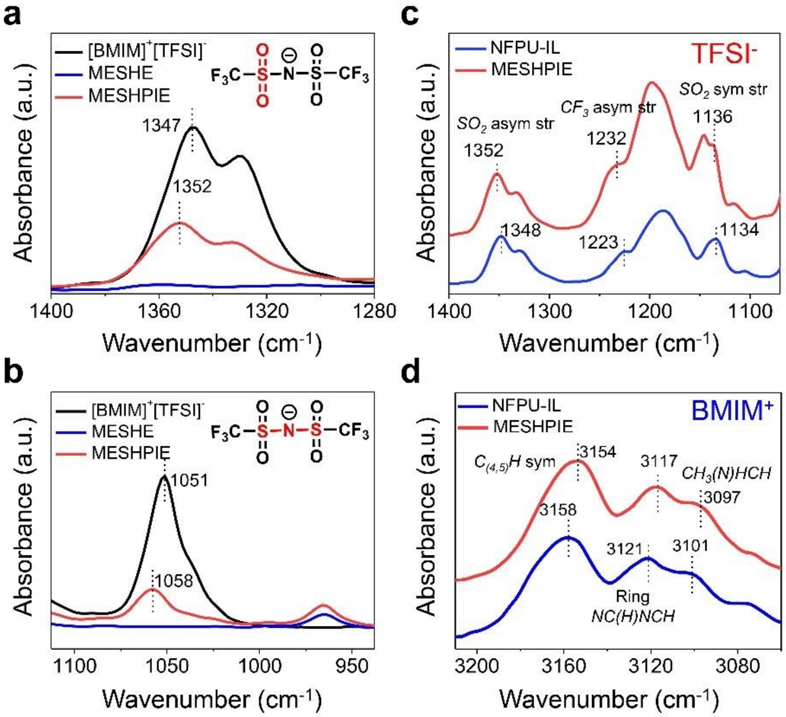

Fig. R9 | Molecular characterization of MESHPIE. ATR-FTIR characterization of $[BMIM]^{+}[TFSI]^{-}$, MESHE, and MESHPIE to verify the ion-dipole interaction. **a** The spectral range of 1400–1280 cm^{-1} and **b**, 1120–920 cm^{-1} corresponds to the stretching of $TFSI^{-}$ vibrational bands in MESHPIE owing to the weakened Coulomb forces generated by the ion-dipole interaction effect on the cations. ATR-FTIR spectra in the spectral regions of **c**, 1400–1000 cm^{-1} (pertaining to $TFSI^{-}$ stretching) and **d**, 3210-3060 cm^{-1} (pertaining to $BMIM^{+}$ stretching) of MESHPIE and NFPU-IL (used as reference).

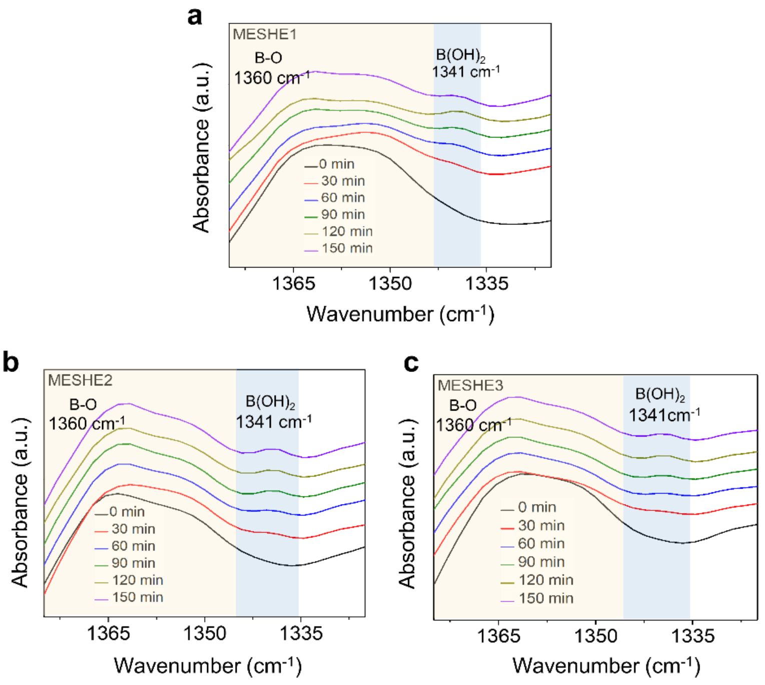

Fig. R10 | ATR–FTIR spectra of **a**, MESHE1, **b**, MESHE2 and **c**, MESHE3 in the spectral regions of 1380–1335 cm^{-1} attributed to B-O group and $B(OH)_2$ group at different water immersion times.

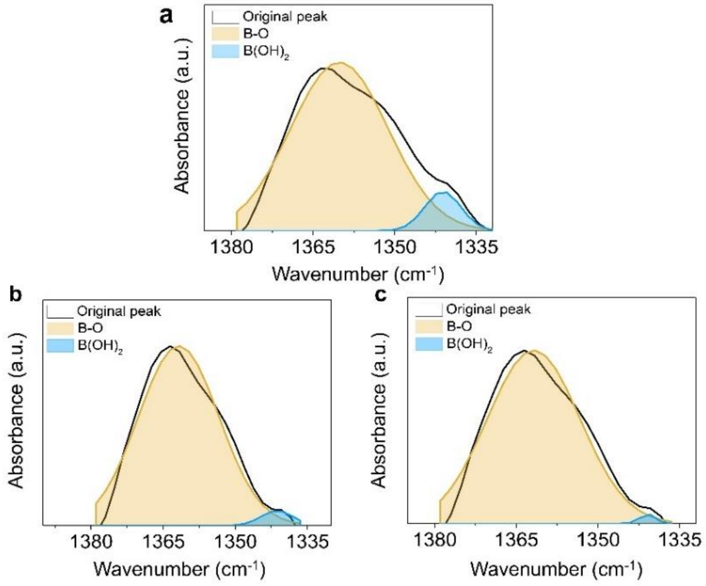

Fig. R11 | Deconvoluted ATR–FTIR spectra peaks in the spectral regions 1380–1335 cm^{-1} (B-O and $B(OH)_2$ stretching) of **a**, MESHE1, **b**, MESHE2 and **c**, MESHE3 showing the water intake of the MESHEs after 150 min.

Q6) *The formats for all the references should be carefully checked to comply with the journal's requirements.*

<u>Response:</u> We appreciate the reviewer for pointing this out. We have revised the format of the references to comply with Nature Communications' requirements.

Reply to Reviewer #3

The authors synthesized an ultrafast underwater molecularly engineered self-healing piezo-ionic elastomer (MESHPIE) inspired by the cephalopod's suckers, which possessed self-healing properties and mechanosensitive ion channels. They integrated a material-based device into an underwater submarine for signal transmission and LED illumination to demonstrate its potential for underwater robotics. However, the MESHPIE-based device characterizations and demonstrations are insufficient and require further improvements and analysis. Major revision is needed before further consideration. Below are some comments:

Response: We appreciate the comments from the reviewer. We have revised the manuscript according to the reviewer's comments.

Q1) *It is mentioned in the introduction that "However, these materials exhibited relatively low self-healing efficiencies and speeds owing to the limited synergistic effect between the hydrophobic groups and dynamic bonds". Corresponding data need to be provided for the relatively low self-healing efficiencies and speeds here.*

Response: We thank the reviewer for this important comment. In the introduction, we mentioned the lower underwater self-healing efficiency and speed of existing literature, which were depicted in **Fig. 2i** of the original manuscript. In addition, **Fig. 2i** also contains the following data of underwater self-healing speed. The corresponding data are shown in **Table R3** below for clear reference.

Table R3. Literature references of self-healing speeds and efficiencies of underwater self-healing materials.

No	Reference	Underwater self-healing speed	Underwater self-healing efficiency
1	Xu, Liguo, et al. "A Transparent, Highly Stretchable, Solvent-Resistant, Recyclable Multifunctional Ionogel with Underwater Self-Healing and Adhesion for Reliable Strain Sensors." Advanced Materials 33.51 (2021): 2105306.	1.0 µm/min	85% in 24 hours
2	Khatib, Muhammad, Zohar, Orr, Saliba, Walaa, Srebnik, Simcha and Haick Hossam. "Highly efficient and water-insensitive self-healing elastomer for wet and underwater electronics." Advanced Functional Materials 30.22 (2020): 1910196.	0.02 µm/min	80-90% in 24 hours

3	Zhang, Yucheng, et al. "Highly transparent, underwater self-healing, and ionic conductive elastomer based on multivalent ion–dipole interactions." Chemistry of Materials 32.15 (2020): 6310-6317.	2.78 μm/min	34% in 12 hours
4	Li, Mengqing, et al. "A hydrophobic eutectogel with excellent underwater Self-adhesion, Self-healing, Transparency, Stretchability, ionic Conductivity, and fully recyclability." Chemical Engineering Journal 472 (2023): 145177.	3.33 μm/min	62.5% in 24 hours
5	He, Cyuan-Lun, et al. "Super Tough and Spontaneous Water-Assisted Autonomous Self-Healing Elastomer for Underwater Wearable Electronics." Advanced Science 8.21 (2021): 2102275.	4.2 μm/min	66.6% in 40 mins

To address the reviewer's concern, the following references have been cited in the introduction of the revised manuscript.

[10] Xu, L. et al. A Transparent, Highly Stretchable, Solvent-Resistant, Recyclable Multifunctional Ionogel with Underwater Self-Healing and Adhesion for Reliable Strain Sensors. Adv Mater **33**, e2105306 (2021)
[11] Khatib, Muhammad, Zohar, Orr, Saliba, Walaa, Srebnik, Simcha and Haick Hossam. "Highly efficient and water-insensitive self-healing elastomer for wet and underwater electronics." Adv. Funct. Mater. **30**, 1910196, (2020)
[12] Zhang, Y. et al. Highly Transparent, Underwater Self-Healing, and Ionic Conductive Elastomer Based on Multivalent Ion–Dipole Interactions. Chemistry of Materials **32**, 6310-6317 (2020)
[13] Li, Mengqing, et al. "A hydrophobic eutectogel with excellent underwater Self-adhesion, Self-healing, Transparency, Stretchability, ionic Conductivity, and fully recyclability." Chemical Engineering Journal, **472**, 145177 (2023).
[14] He, Cyuan-Lun, et al. "Super Tough and Spontaneous Water-Assisted Autonomous Self-Healing Elastomer for Underwater Wearable Electronics." Adv. Sci. **8**, 2102275 (2021).

Q2) *The labels in Figure 1 need to be improved. For example, the cations in Figure 1c are Ca2+ ions, which need to be labeled in the figure.*

Response: We appreciate the reviewer for the valuable comments. We have revised **Fig.1** in the revised Manuscript to include all necessary labels as shown in **Fig. R12**.

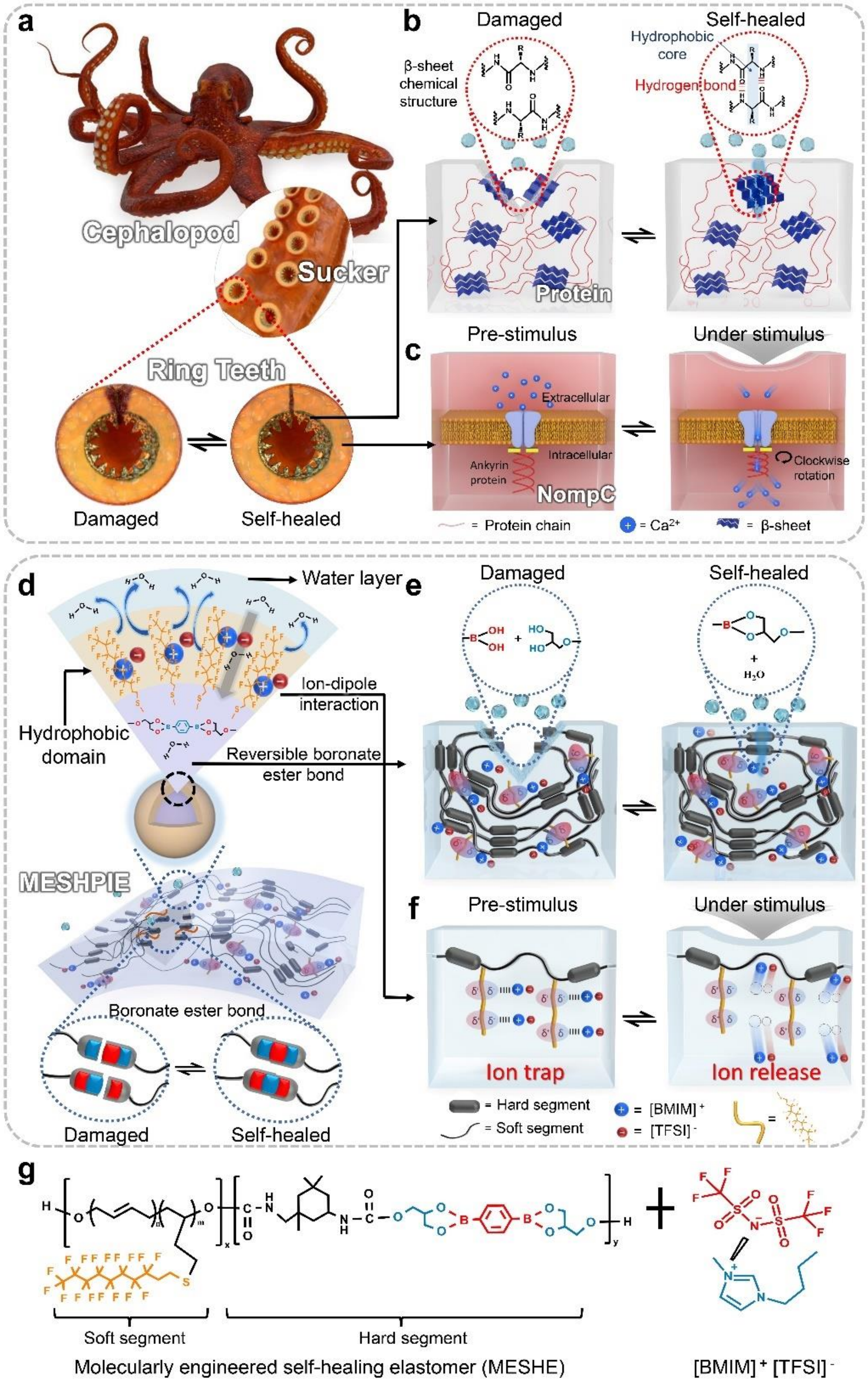

Fig. R12 | Molecular chemistry of the cephalopod and conceptual design principle of MESHPIE. a Schematics of cephalopod and ring teeth (RT) structures. **b** The RT proteins within the cephalopod's suckers consist of numerous hydrogen bonds and hydrophobic core, which enable self-healing in both ambient and aquatic conditions The hydrogen bonds self-assemble into segmented semicrystalline morphology (amorphous and β-sheets). **c** Unique mechanoreceptor NompC composed of tethered ion channels observed in cephalopod's suckers. **d** Chemical structure representation of MESHPIE consisting of hydrophobic domain and reversible boronate ester bonds for self-healing in both ambient and aquatic environments. **e** Schematic illustration depicting the structure of MESHPIE, consisting of hard and soft segments, emulating the RT protein structure of the cephalopod. **f** Piezo-ionic

dynamics (trap and release mechanism) emulating the ion dynamics of the cephalopod. g Representation of the design chemistry of MESHE and ionic liquid.

Q3) *In Figure 2f, quantitative characterizations should be provided to demonstrate the absence of water leakage when the material is loaded.*

Response: We thank the reviewer for this comment. The experiment in **Fig. 2f** original Manuscript, was conducted to demonstrate that not only is our self-healing material capable of autonomous self-healing of scratch and microscale damages but also can self-heal macroscale damages (*Nat Rev Mater* **5**, 562–583 (2020); *European Polymer Journal* **53**, 118–125 (2014)) in underwater conditions. As can be vividly seen in the original Supplementary Movie 1, no breaking or water leakage was observed even under pressure.

Nonetheless, to clarify the absence of water leakage, we have reproduced this demonstration over an electronic scale to quantitatively characterize the absence of water leakage even under applied pressure. As clearly shown in **Fig. R13** and revised Supplementary Movie 1, no water drop was recorded by the electronic scale when pressure was applied, as evidenced by the stable readings. This demonstrates and confirms the absence of water leakage owing to the excellent underwater self-healing capabilities of our material.

To address the reviewer's concern, we have replaced **Fig. 2** with **Fig. R13** in the revised Manuscript and Supplementary Movie 1 with the revised Supplementary Movie 1.

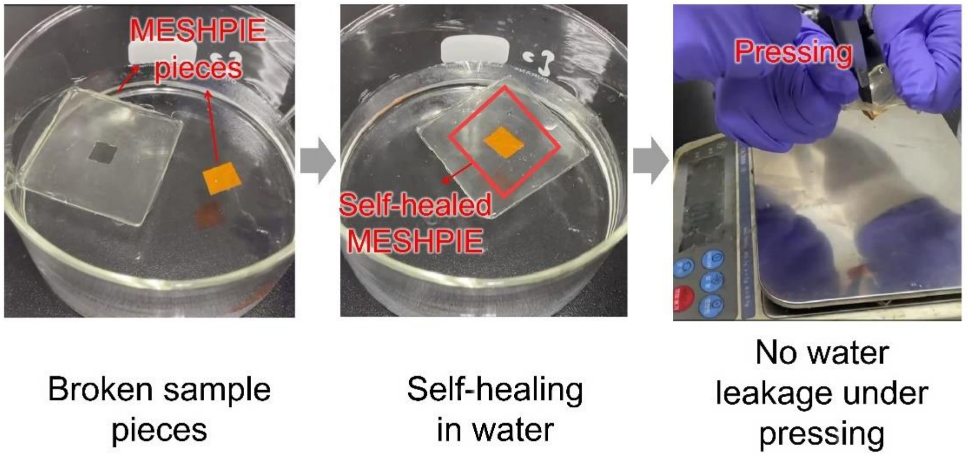

Fig. R13 | Cut and spliced scenario of two different MESHPIE pieces, healed together underwater to withstand breaking under pressure.

Q4) *Figure 2i compares the self-healing speeds of different self-healing materials at different temperatures. The author tested the self-healing speed at room temperature. How about the effect of temperature on its self-healing speed? And how about the self-healing speed at higher temperature?*

Response: We appreciate the reviewer for raising this important point. It is well known that elevated

temperatures cause the generation of high segmental chain mobility to facilitate faster healing of the damage area (*Nat Rev Mater* **5**, 562–583 (2020)). As expected, at higher temperatures, our material exhibited faster healing speeds owing to enhanced segmental chain mobility (**Fig. R14**).

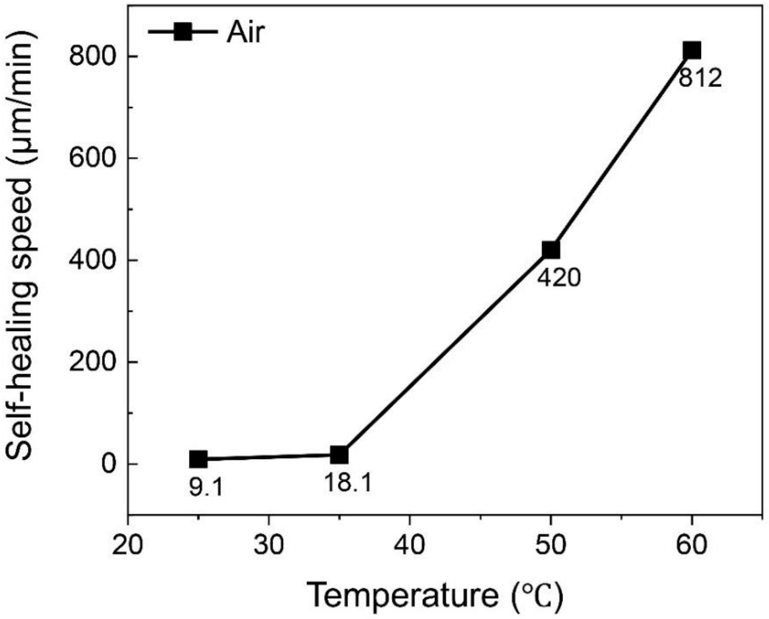

Fig. R14 | Self-healing speeds of MESHPIE at different temperatures in air.

Q5) *In Figure 4b, the authors state that the ion trap and release before and after inducing pressure generate a low initial capacitance and a high final capacitance, respectively, owing to the efficient pumping of ions. Is there evidence that the material undergoes ion release under pressure stimulation?*

Response: We thank the reviewer for the valuable comments. As discussed in the original manuscript under the section "Characterization of piezo-ionic dynamics" and Supplementary Note 4, the relationship between piezo-ionic dynamics and their corresponding changes in complex impedance behavior were analyzed to provide clear evidence to support the ion release under pressure stimulation. Please see these sections for detailed explanations. Nonetheless, the decrease in impedance with increasing pressure (**Supplementary Fig. 16a**, original Supplementary Information), owing to release of more free ions that were initially trapped, and the shift of charge relaxation frequency (τ^{-1}) in the Bode plot towards higher frequencies (**Fig. 4c**, original Manuscript) owing to faster ionic atmosphere relaxation indicating increased free ion concentration owing to ion release under pressure in MESHPIE, which was not an observed tendency in NFPU-IL (without C–F groups)-based device (**Supplementary Fig. 16b** and 16c, original Supplementary Information), are clear evidence to support that the material undergoes ion release under pressure stimulation.

Q6) *In Figure 4h, the response time of the device is 281 ms, which is slower than other ionic conductors-based devices. Why is the response time so slow? Is it possible to improve the response time?*

Response: We appreciate the comment from the reviewer. The slow response time recorded in **Fig. 4h** original Manuscript, can be attributed to the lower frequency (100 Hz) utilized to achieve the most efficient electric double layer (EDL) formation (*Science* **370**, 961–965 (2020)), which resulted in slower response time due to the gradual changes in capacitance at lower frequency. Increasing the frequency to 1000 Hz results in a faster response time of 78 ms (**Fig. R15**).

To address the reviewer's concern, we have replaced **Fig. 4h** with **Fig. R15** in the revised manuscript.

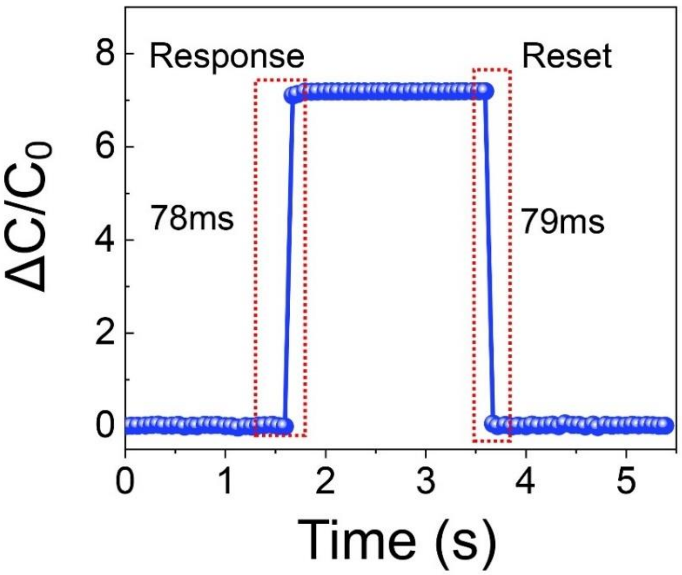

Fig. R15 | Transient response time at a loading pressure of 7.2 kPa (1 mV applied bias at 1000 Hz).

Q7) *Although the MESHPIE can be placed in water, other components of the MESHPIE-based devices might fail? Please comment on this.*

Response: We thank the reviewer for this comment. As discussed on page 9 of the original manuscript, the MESHPIE-based device consists of MESHPIE sandwiched between two deformable Ag nanowire (AgNW)/MESHE2 electrodes. The electrodes were developed using superb hydrophobic MESHE2 (the base material for developing MESHPIE) as substrate to protect the AgNW from direct interference with water molecules (see "Method" section subheading "Fabrication of self-healing electrodes" for details). Moreover, to establish connection with measuring instruments, encapsulated Ag wires were used to prevent any interference against water molecules. Therefore, all components of the MESHPIE-based device were hydrophobically insulated from water molecules, thus no failure was detected.

Q8) *If the devices based on this material are to be used in water or air, their stability is very important. How about the stability of the material in water? How about the shelf-life?*

Response: We appreciate the reviewer for the valuable comments. In order to facilitate the application of our device in both underwater and air, we engineered a robust hydrophobic material by introducing C–F side chains to mitigate the potential interference of substantial influx of water molecules. Simultaneously, to demonstrate the sustained hydrophobicity of the material, we subjected our material with different C–F group content to a continuous five-day water immersion to investigate their stability in water. As shown in **Supplementary Fig. 6**, the water contact angles of the various samples affirm the excellent stability in underwater conditions owing to their stable hydrophobicity. As proof of hydrophobicity, **Supplementary Fig. 6** is included here as **Fig. R16** for easy reference to the reviewer.

Additionally, the MESHPIE-based device was submerged in water for a period of 10 days to support its stability in underwater condition over a longer period. As illustrated in **Fig. R17**, the device exhibited a slight increase in mass after 1 day of immersion due to the ingress of small quantities of water. The presence of excellent hydrophobic interactions effectively repelled the majority of interfacial water molecules, resulting in minimal water absorption. Subsequently, over the remaining 9 days, the device's mass remained virtually unchanged, indicating the outstanding stability and possible longer shelf-life of our device in underwater conditions. Moreover, the nearly overlapping impedance curves of the device immersed in water for the different days (**Fig. R18**), also emphasize the stable underwater electrical performance of our device, reaffirming its significant potential for underwater applications.

To emphasize the device stability in underwater conditions, we have added the following sentences on page 8 and **Fig. R17** and **Fig. R18** as **Supplementary Figs. 23a** and **23b**, respectively, to the revised Supplementary Information.

"Additionally, the MESHPIE-based device was submerged in water for a period of 10 days to support its stability in underwater condition over a longer period. As illustrated in Supplementary Fig. 23a, the device exhibited a slight increase in mass after 1 day of immersion owing to the ingress of small quantities of water. The presence of excellent hydrophobic interactions effectively repelled the majority of interfacial water molecules, resulting in minimal water absorption. Subsequently, over the remaining 9 days, the device's mass remained virtually unchanged, indicating outstanding stability. Moreover, the nearly overlapping impedance curves of the device immersed in water for the different days (Supplementary Fig. 23b), also emphasize the stable underwater electrical performance of our device, reaffirming its significant potential for underwater applications."

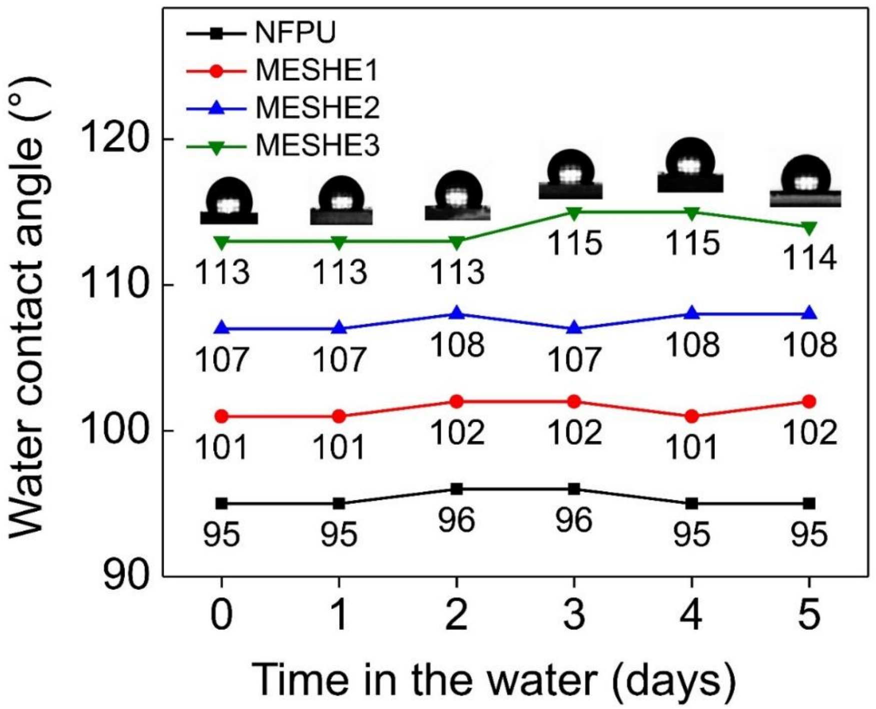

Fig. R16 | Changes of water contact angles. Images of the water contact angles of the various PUs and calculated angles representing the changes in their hydrophobicity after being immersed in water for five consecutive days.

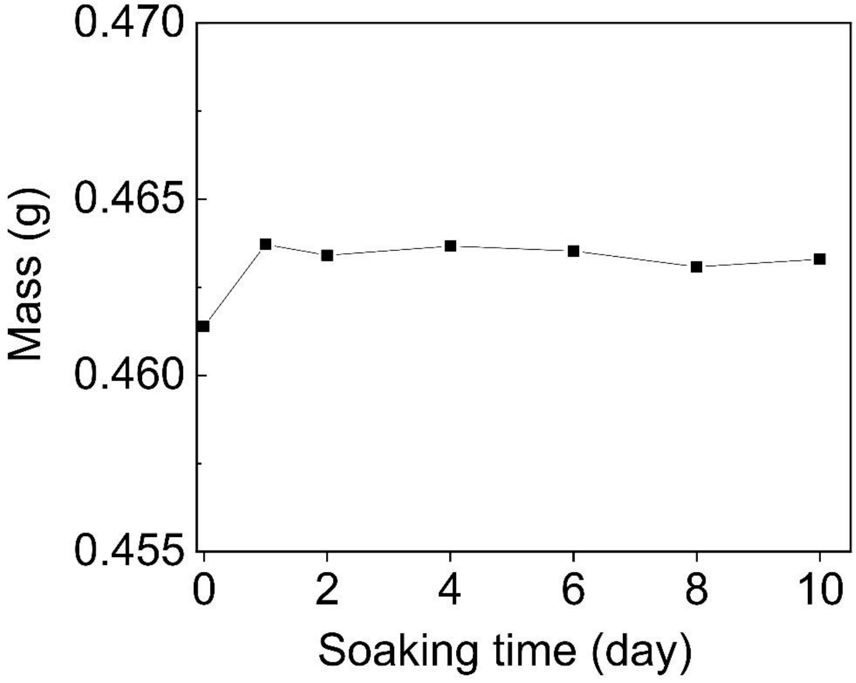

Fig. R17 | Mass change rate. Changes in the mass of the MESHPIE-based device after being immersed in water for different days.

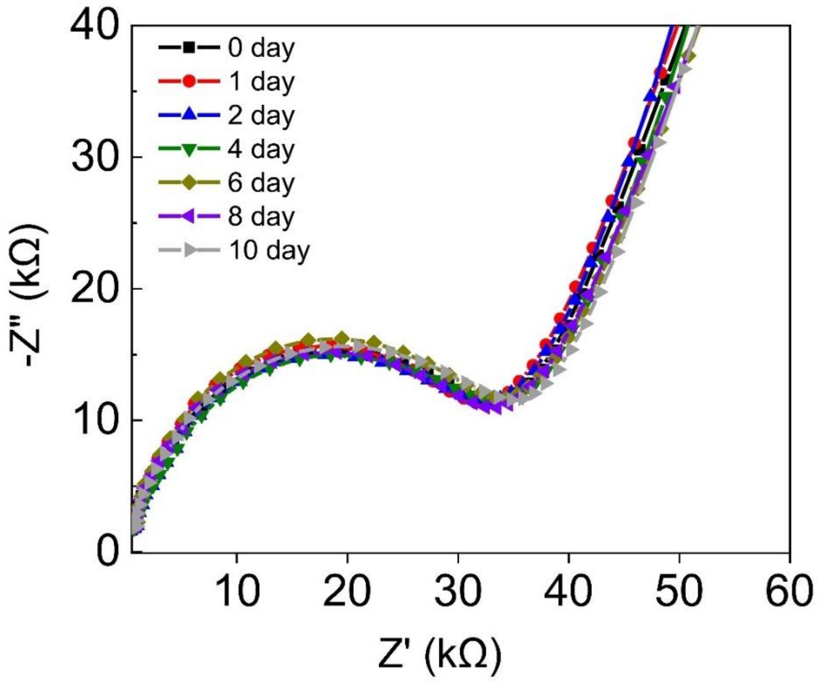

Fig. R18 | Changes in impedance (electrical properties) after soaking in water. Impedance Nyquist plots of MESHPIE-based device after being immersed in water for different days.

Q9) *The demonstration in Figure 5e does not effectively reflect the advantages of the MESHPIE-based device. Most ionic conductors-based pressure sensor can do.*

Response: We thank the reviewer for this comment. We totally agree that some ionic conductor-based pressure sensors can perform this demonstration. Nevertheless, we performed this demonstration in this study to affirm that our material is also touch-sensitive showcasing its capability to detect human interactive activities through touch, owing to its remarkable mechanosensitivity.

REVIEWERS' COMMENTS

Reviewer #1 (Remarks to the Author):

The manuscript in the current version is well organized with sufficient data and in-depth discussion, except for some minor issues to be revised before publication:

1. A figure depicting the “Healing efficiency” of MESHPIE in air should be added in Supplementary Fig. 14.

2. Figure numbers should be double-checked, for example, in Supplementary Information, figure numbers mentioned in the sentence “Supplementary Figs. 14a and 14b present the rheological oscillation strain sweep tests of MESHPIE in air and with water, respectively.” should be Fig.15.

Reviewer #2 (Remarks to the Author):

I would like to support the acceptanceof this manuscript

Reviewer #3 (Remarks to the Author):

The authors have carefully addressed the review comments. The revised manuscript is suitable for publication.

Reviewers' Comments

Reviewer #1 (Remarks to the Author):

The manuscript in the current version is well organized with sufficient data and in-depth discussion, except for some minor issues to be revised before publication:

Response: We thank the reviewer for the positive assessment of our work.

Q1) *A figure depicting the "Healing efficiency" of MESHPIE in air should be added in Supplementary Fig. 14.*

Response: We thank the reviewer for this valuable comment. We have inserted a figure (graph) depicting the healing efficiency of MESHPIE in air (Supplementary Fig. 14) in the revised Supplementary Information as shown in **Fig. R1**.

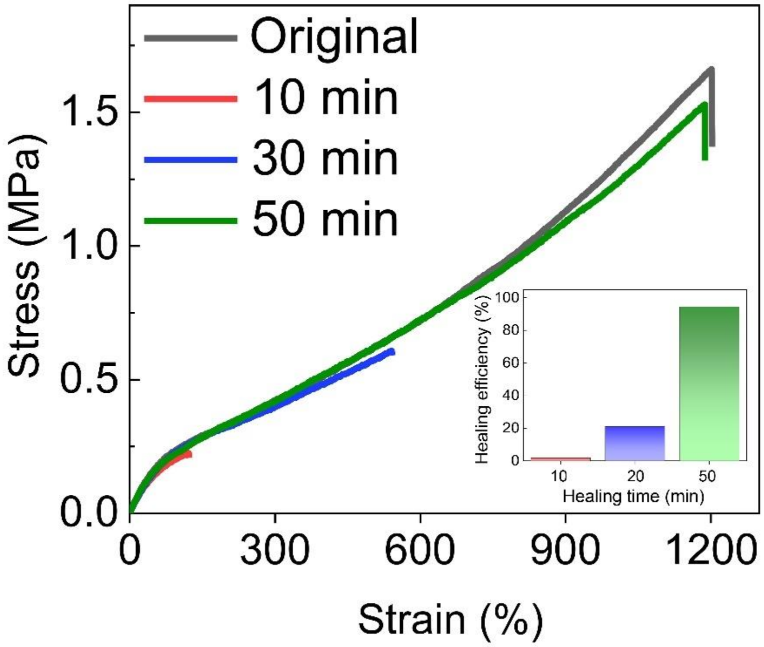

Fig. R1 | Stress–strain curves at various healing times in air. Recovery of mechanical properties of self-healed MESHPIE at various healing times in air. Insert is a bar graph depicting the self-healing efficiency of MESHPIE in air.

Q2) *Figure numbers should be double-checked, for example, in Supplementary Information, figure numbers mentioned in the sentence "Supplementary Figs. 14a and 14b present the rheological oscillation strain sweep tests of MESHPIE in air and with water, respectively." should be Fig.15.*

Response: We thank the reviewer for pointing this out. We have double-checked and rectified the figure numbers.

Reviewer #2 (Remarks to the Author):

I would like to support the acceptance of this manuscript.

Response: We appreciate the reviewer for the positive assessment of our work.

Reviewer #3 (Remarks to the Author):

The authors have carefully addressed the review comments. The revised manuscript is suitable for publication.

Response: We thank the reviewer for recommending our work for publication.
